# *Dmrt1* is the only male pathway gene tested indispensable for sex determination and functional testis development in tilapia

**DOI:** 10.1371/journal.pgen.1011210

**Published:** 2024-03-27

**Authors:** Shuangshuang Qi, Shengfei Dai, Xin Zhou, Xueyan Wei, Ping Chen, Yuanyuan He, Thomas D. Kocher, Deshou Wang, Minghui Li

**Affiliations:** 1 Integrative Science Center of Germplasm Creation in Western China (CHONGQING) Science City, Key Laboratory of Freshwater Fish Reproduction and Development (Ministry of Education), Key Laboratory of Aquatic Science of Chongqing, School of Life Sciences, Southwest University, Chongqing, China; 2 Department of Biology, University of Maryland, College Park, Maryland, United States of America; University of Wuerzburg, GERMANY

## Abstract

Sex is determined by multiple factors derived from somatic and germ cells in vertebrates. We have identified *amhy*, *dmrt1*, *gsdf* as male and *foxl2*, *foxl3*, *cyp19a1a* as female sex determination pathway genes in Nile tilapia. However, the relationship among these genes is largely unclear. Here, we found that the gonads of *dmrt1*;*cyp19a1a* double mutants developed as ovaries or underdeveloped testes with no germ cells irrespective of their genetic sex. In addition, the gonads of *dmrt1*;*cyp19a1a*;*cyp19a1b* triple mutants still developed as ovaries. The gonads of *foxl3*;*cyp19a1a* double mutants developed as testes, while the gonads of *dmrt1*;*cyp19a1a*;*foxl3* triple mutants eventually developed as ovaries. In contrast, the gonads of *amhy*;*cyp19a1a*, *gsdf*;*cyp19a1a*, *amhy*;*foxl2*, *gsdf*;*foxl2* double and *amhy*;*cyp19a1a*;*cyp19a1b*, *gsdf*;*cyp19a1a*;*cyp19a1b* triple mutants developed as testes with spermatogenesis via up-regulation of *dmrt1* in both somatic and germ cells. The gonads of *amhy*;*foxl3* and *gsdf*;*foxl3* double mutants developed as ovaries but with germ cells in spermatogenesis due to up-regulation of *dmrt1*. Taking the respective ovary and underdeveloped testis of *dmrt1*;*foxl3* and *dmrt1*;*foxl2* double mutants reported previously into consideration, we demonstrated that once *dmrt1* mutated, the gonad could not be rescued to functional testis by mutating any female pathway gene. The sex reversal caused by mutation of male pathway genes other than *dmrt1*, including its upstream *amhy* and downstream *gsdf*, could be rescued by mutating female pathway gene. Overall, our data suggested that *dmrt1* is the only male pathway gene tested indispensable for sex determination and functional testis development in tilapia.

## Author summary

In vertebrates, the antagonistic effect between male and female pathway genes determines the development of gonads towards ovaries or testes and maintains the final gonadal phenotype. Using tilapia as animal model, we found that the *dmrt1* mutant gonad could not be rescued to a functional testis by mutation of female pathway genes *foxl2*, *foxl3* and *cyp19a1a*. In contrast, the *amhy* and *gsdf* mutant gonad can be rescued to functional testis by mutating female pathway genes via up-regulation of *dmrt1*. Our data strongly highlight the indispensable role of *dmrt1* in male sex determination and testicular development. The possible reason is that it is the only sex determining pathway gene identified so far expressing in both germ cells and somatic cells, while others were exclusively expressed in somatic cells or germ cells. This might be why *dmrt1* is commonly used as the key male pathway gene in vertebrates and even in some invertebrates.

## Introduction

Sex determination (SD), which is controlled by genetic or environmental factors, or both in vertebrates [1,2], is a hot topic in developmental and reproductive biology. Since the discovery of the first fish master sex determining (MSD) gene *dmy*/*dmrt1by* in medaka (*Oryzias latipes*) in 2002 [3, 4], with the development of genome sequencing and genome editing technologies, a number of MSD genes have been identified in fish species in the past two decades [2,5]. In contrast to most mammals that sex is genetically determined by *SRY*/*Sry* on the Y chromosome [6, 7], the MSD genes in fish exhibit diversity and rapid turnover even in closely related species [5]. It is worth noting that most of the fish MSD genes identified belong to the TGF-β signaling pathway, including *amhy*, *amhr2y*, *gdf6y*, *bmpr1bby* and *gsdfy* [5,8]. In Nile tilapia (*Oreochromis niloticus*), a gonochoristic teleost with an XX/XY SD system, a tandem duplicate of *amh* on the Y chromosome, named as *amhy*, is identified as the MSD gene by our group [9]. Although the MSD genes vary extensively among fish species, the downstream factors are more or less conserved [10,11]. The *dmrt1*, *gsdf* as male pathway genes and *foxl2*, *foxl3* and *cyp19a1a* as female pathway genes have been identified in Nile tilapia [12–14] and some other fishes [10, 11]. It is well accepted that antagonistic actions between female and male pathway genes determine and maintain the gonadal sex in vertebrates [1]. However, to date, the genetic interactions between the identified male and female pathway genes in fish species are largely unknown.

Estrogen plays an important role in female ovarian differentiation in non-mammalian vertebrates [15]. It is produced through the conversion of androgens by steroidogenic enzyme CYP19A1 (aromatase) [16]. Administration of aromatase inhibitor (AI) or exogenous estrogen (E2) induces sex reversal (SR) in a large number of fish species, including tilapia [15,17,18]. Consistently, mutation of *cyp19a1a*, which is only expressed in somatic cells, leads to testicular development in zebrafish (*Danio rerio*), tilapia and medaka [13,19,20]. In addition, mutation of gonadal somatic cell expressed transcription factor *foxl2*, an evolutionarily conserved female pathway gene that directly regulating *cyp19a1a* expression and E2 synthesis [21], results in testicular development in zebrafish, tilapia and gibel carp (*Carassius gibelio*) [13,22,23]. Furthermore, studies have shown that mutation of germline specific expressed *foxl3*, a paralog of *foxl2* found in most vertebrates except placental mammals [24–26], results in germ cell SR in ovary of XX tilapia and medaka [14,27]. In zebrafish, mutation of *foxl3* (also named as *foxl2l*) results in all male development [28]. However, the critical role of *foxl3* in sperm-egg fate decision has not been demonstrated outside of the fish clade. So far, in tilapia, our transcriptional regulation and loss-of-function analyses reveal a possible female pathway *foxl2*-*cyp19a1a*-*foxl3* in controlling and maintaining ovary fate.

Transcription factor *dmrt1* is a highly conserved regulator involved in male SD and sex differentiation across vertebrates [2]. In mammals, although Dmrt1 is not required for SD because the gonads of XY *Dmrt1* mutants still develop as testes in mouse (*Mus musculus*) [29], ectopic expression of Dmrt1 in XX gonads results in female-to-male SR, suggesting that *Dmrt1* retains the capacity of SD [30]. A recent study in rabbit (*Oryctolagus cuniculus*) has shown that *dmrt1* is the sex determining gene as the gonads of the XY *Dmrt1* mutants differentiate into ovaries [31], indicating that Dmrt1 has a SD role in certain mammals. In bird, loss of one *dmrt1* copy in ZZ chicken (*Gallus gallus*) leads to ovary in place of testis development [32]. In reptile, *dmrt1* knockdown in ZZ or overexpression in ZW red-eared slider turtle (*Trachemys scripta*) embryos reverse their gonadal phenotypes [33]. In fish, the gonads of the zebrafish and tilapia *dmrt1* mutants directly develop into ovaries [14, 34], while in medaka, the gonads of the *dmrt1* mutants first develop into testes and then transdifferentiate into ovaries [35], probably due to the compensation of *dmy*/*dmrt1by*. Anyway, the gonads of the *dmrt1* mutants finally develop as ovaries. Additionally, *dmrt1* and its duplicates are even identified as the MSD genes in several fish species [3,4,36–38]. However, it is still unknown why *dmrt1* is so important for males in vertebrates.

The establishment of animal models with disruption of male and female pathway genes simultaneously is crucial for elucidating their genetic interaction in sex differentiation. It is particularly noteworthy that the ovary of *dmrt1* mutants cannot be rescued to a functional testis by mutation of *cyp19a1a* or AI treatment in fish and bird. For example, 1) In zebrafish, mutations of *cyp19a1a* and/or estrogen receptors (*esr1*/*esr2a*/*esr2b*) fails to rescue the ovaries of *dmrt1* mutants to testes [39,40]. 2) In tilapia, the ovaries of *dmrt1* mutants cannot be rescued to functional testes by AI treatment or mutation of *foxl2* or *foxl3* [14]. 3) In chicken, administration of AI is unable to rescue the SR of ZZ *Dmrt1* mutants [32]. Most importantly, these studies were conducted in different species, and up to now, it is necessary to study how the gonads will develop when *dmrt1* and key genes of the female pathway, including *foxl2*, *foxl3* and *cyp19a1a*, are mutated simultaneously in one species. Additionally, teleost fish possess two aromatase genes, *cyp19a1a* (encoding ovarian aromatase) and *cyp19a1b* (encoding brain aromatase) [41]. There is no doubt that brain aromatase can catalyze the conversion of testosterone to estrogen [42]. In zebrafish, the follicles of *dmrt1*;*cyp19a1a* double mutants could develop into previtellogenic stage [39,40], which cannot rule out the role of Cyp19a1b that can produce E2. Therefore, it is unknown whether this is caused by the indispensable role of *dmrt1* in male SD, or the compensation of *cyp19a1b* for the insufficient estrogen caused by *cyp19a1a* deficiency.

Besides Dmrt1, the members of TGF-β signaling pathway, especially *amh/amhr2* and *gsdf*, play critical role in male SD and sex differentiation in a number of fish species as well [8]. In XY tilapia, mutation of *amhy* or *gsdf* results in up-regulation of Cyp19a1a and male-to-female SR [9,12]. This sex reversed ovary of XY *amhy* and *gsdf* mutants can be rescued to functional testis by AI treatment [12,43]. Consistently, mutation of *cyp19a1a* rescues the SR in the XY *amhy* mutant tilapia at 90 days post fertilization (dpf) [43]. Similarly, AI treatment rescue the male-to-female SR caused by *gsdf* mutation in gibel carp and *amhr2* mutation in Japanese flounder (*Paralichthys olivaceus*) [44,45]. Therefore, induction of Cyp19a1a expression is responsible for driving male-to-female SR in *amhy*/*amhr2* and *gsdf* mutants. Nevertheless, it is unknown whether mutation of other female pathway genes, such as *foxl2* and *foxl3*, can rescue the male-to-female SR caused by mutation of *amhy* and *gsdf*, both of which were exclusively expressed in somatic cells [9,12].

It is well known that the vertebrate sex is highly plastic. Females with differentiated or even mature ovary are masculinized to functional males by long-term AI treatment in tilapia, medaka and zebrafish, indicating that estrogen is also critical for female sex maintenance [46–48]. Recent study in our group demonstrated that AI treatment fails to induce testis development in XX *dmrt1* mutants and E2 treatment fails to induce oocyte differentiation in XY *foxl3* mutants in tilapia [14]. Therefore, sex maintenance is suggested to be regulated by transcription factors (*dmrt1* and *foxl3*) and estrogen, which are required to be expressed continuously in the gonads. Like the other MSD genes [1–5], *amhy* expression is transiently up-regulated during the period of SD (from 3 to 7 days after hatching, dah) [43]. How these male and female pathway genes interact and work together to maintain the gonadal fate is unknown.

The roles of male pathway genes *amhy*, *dmrt1* and *gsdf* and female pathway genes *foxl2*, *cyp19a1a* and *foxl3* in SD and sex differentiation have been demonstrated by generation of single gene mutation line in tilapia [9,12–14]. However, the knowledge about interactions of these genes, especially between genes expressed in somatic cells and germ cells, in gonadal fate decision and maintenance is still limited. The Nile tilapia is a good animal model for studying SD and sex differentiation because genetic all-females and genetic all-males are available [49]. A number of genes show sexual dimorphic expression in gonads at 5 dah, the critical time for SD [50, 51]. For instance, *amhy* and *gsdf* are expressed exclusively in somatic cells of the XY gonads, whereas *foxl2* and *cyp19a1a* are expressed exclusively in somatic cells of the XX gonads [9,12,21,43]. *dmrt1* is expressed in both somatic cells and germ cells in XY testis, while *foxl3* is expressed exclusively in germ cells in XX ovary [14]. The first morphological differentiation of gonad occurs in tilapia between 20 and 25 dah with appearance of the ovarian cavity in the XX ovary. The efferent duct in the XY testis is observed at around 40 dah. Additionally, difference in germ cell number between sexes is observed during early gonad differentiation. The germ cells continue to proliferate in XX ovary from 9 dah, while germ cell proliferation is not observed in XY testis until 15 dah [52]. The germ cells undergo meiosis to generate oocytes in XX gonads from 25 dah onwards, but meiosis does not initiate to generate spermatocytes in XY gonads until 60 dah (S1 Fig).

In the present study, we established 9 double (*dmrt1*;*cyp19a1a*, *amhy*;*cyp19a1a*, *gsdf*;*cyp19a1a*, *cyp19a1a*;*cyp19a1b*, *amhy*;*foxl3*, *gsdf*;*foxl3*, *cyp19a1a*;*foxl3*, *amhy*;*foxl2*, *gsdf*;*foxl2*) and 4 triple (*dmrt1*;*cyp19a1a*;*cyp19a1b*, *amhy*;*cyp19a1a*;*cyp19a1b*, *gsdf*;*cyp19a1a*;*cyp19a1b*, *dmrt1*;*cyp19a1a*;*foxl3*) mutants in Nile tilapia to answer the above questions. Through gonadal phenotype and gene expression analyses of these mutants, we discovered that when the *dmrt1* is mutated, the gonads could not develop as functional testes even if any female pathway gene was disrupted. In contrast, once *dmrt1* is present, SR caused by mutation of any other male pathway genes identified so far could be rescued. Together with the gonadal phenotypes of *dmrt1*;*foxl3*, *dmrt1*;*foxl2* and *foxl3*;*foxl2* double mutants reported previously [14], we concluded that *dmrt1* is the only male pathway gene tested essential for SD and functional testis development in tilapia.

## Results

### Gonads of *dmrt1*^-/-^;*cyp19a1a*^-/-^ double mutants developed as ovaries or underdeveloped testes

**Fig 1:**
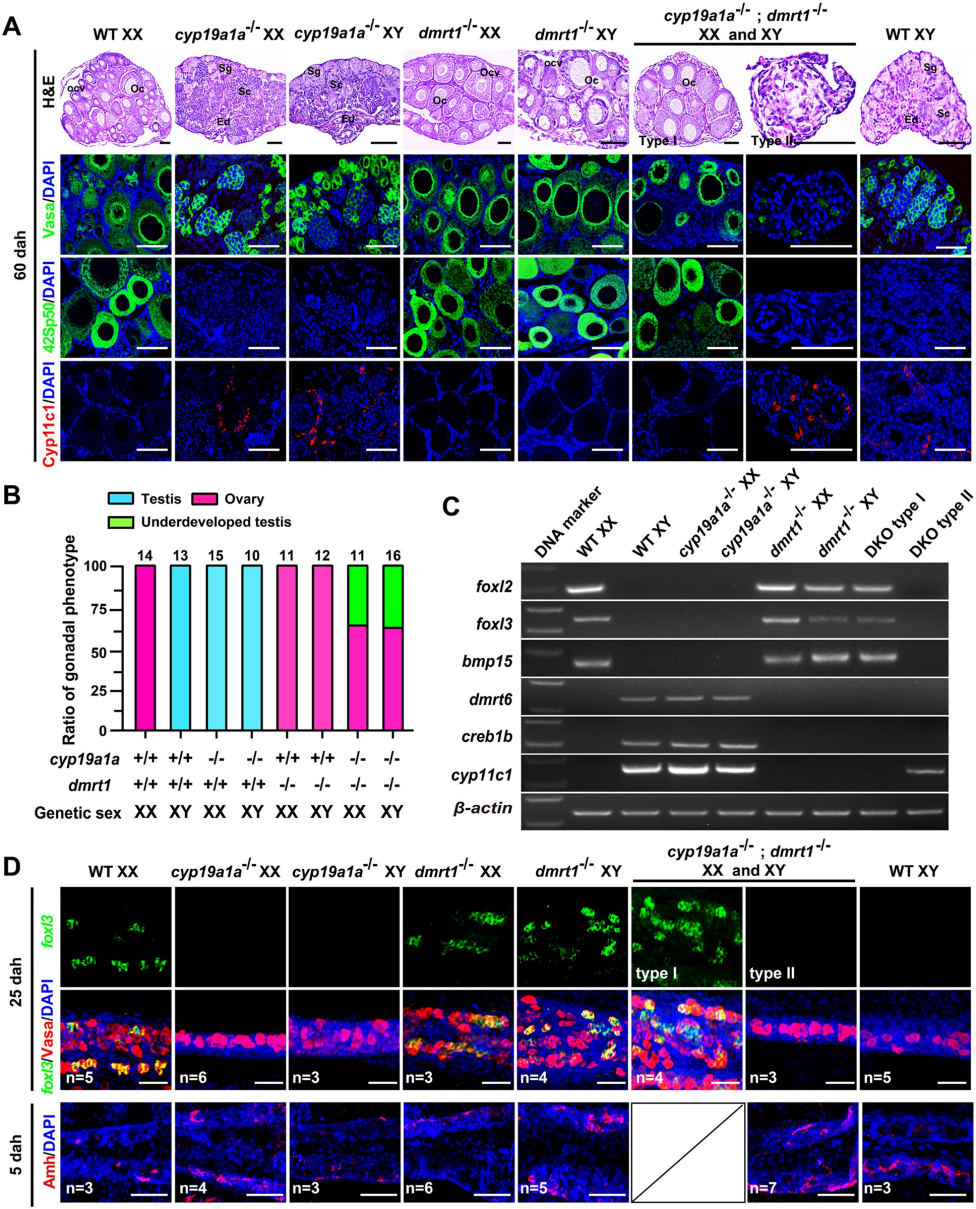
Double mutation of *dmrt1*;*cyp19a1a* resulted in ovary or underdeveloped testis. **(A)** Histological examination of gonads from WT XX, WT XY, XX/XY *dmrt1*^-/-^, XX/XY *cyp19a1a*^-/-^ and XX/XY *dmrt1*^-/-^;*cyp19a1a*^-/-^ tilapia at 60 dah by Hematoxylin and Eosin (H&E) staining. Two gonadal phenotypes, named as type I and type II, were observed in the *dmrt1*^-/-^;*cyp19a1a*^-/-^ double mutants. Gene expressions were analyzed by immunofluorescence (IF) using germ cell marker Vasa, oocyte marker 42Sp50 and Leydig cell marker Cyp11c1. Nuclei were counterstained with DAPI. Sg, spermatogonia. Sc, spermatocyte. Oc, oocyte. Ocv, ovarian cavity. Ed, efferent duct. Scale bars = 40 μm. **(B)** Sex ratios in WT XX, WT XY, XX/XY *dmrt1*^-/-^, XX/XY *cyp19a1a*^-/-^ and XX/XY *dmrt1*^-/-^;*cyp19a1a*^-/-^ tilapia at 60 dah. **(C)** RT-PCR analysis of several female and male specific markers expression in the gonads of WT XX, WT XY, XX/XY *dmrt1*^-/-^, XX/XY *cyp19a1a*^-/-^, and XX/XY *dmrt1*^-/-^;*cyp19a1a*^-/-^ tilapia at 60 dah. *foxl2*, an ovarian somatic cell marker. *foxl3*, an oogonia marker. *bmp15*, an oocyte marker. *dmrt6* and *creb1b*, two spermatocyte markers. *cyp11c1*, a Leydig cell marker. *β-actin* was used as an internal control. WT, wild-type; dah, days after hatching. **(D)** Detection of female specific *foxl3* mRNA (green) expression in the gonads of WT XX, WT XY, XX/XY *dmrt1*^-/-^, XX/XY *cyp19a1a*^-/-^ and XX/XY *dmrt1*^-/-^;*cyp19a1a*^-/-^ tilapia at 25 dah by whole-mount fluorescence *in situ* hybridization and Amh protein expression in these mutants at 5 dah by whole-mount immunofluorescence. Clear differences in the gonadal morphology and *foxl3* expression were observed between the type I and type II gonadal phenotype in the double mutants at 25 dah, while no obvious differences in morphology and Amh expression were observed between the type I and type II gonadal phenotype of the double mutants at 5 dah. Therefore, a panel is missing in Fig 1D. Germ cells (red) were labeled using Vasa antibody. Yellow color indicates the co-expression signals of *foxl3* and Vasa. Scale bars = 40 μm.

Our previous studies showed that Dmrt1 directly binds to the promoter of *cyp19a1a* to repress its transcription [53] and loss of *dmrt1* results in ovary development and up-regulation of *cyp19a1a* expression in XY tilapia when checked at 15 dah [14]. But it is unclear when this up-regulation occurs. In this study, the gonads of the XY *dmrt1*^-/-^ mutants developed as ovaries with previtellogenic follicles at 60 dah (n = 12), same as those of XX *dmrt1*^-/-^ mutants (n = 11) and wild-type (WT) XX (n = 14) (Fig 1A). Whole-mount immunofluorescence (IF) analysis showed that expression of Cyp19a1a was not observed in XY *dmrt1*^-/-^ gonads at 5 dah (S2A Fig), indicating that *dmrt1* is important for male sex differentiation and maintenance. In contrast, loss of *cyp19a1a* results in up-regulation of *dmrt1* expression and testis development in XX tilapia at 90 dah [13]. Consistently, all the gonads of the XX *cyp19a1a*^-/-^ mutants developed as testes at 60 dah (n = 15), same as the XY *cyp19a1a*^-/-^ mutants (n = 10) and WT XY (n = 13) (Fig 1A). We found that the majority of the XX *cyp19a1a*^-/-^ mutants (6/8, 75%) displayed testis development, while a minority of the mutants (2/8, 25%) exhibited testis but with a few dispersed oocytes at 45 dah as demonstrated by IF of Leydig cell (Cyp11c1) and oocyte (42Sp50) markers. All the gonads of the XX *cyp19a1b*^-/-^ mutants (n = 11) developed as ovaries at 45 dah (S2B and S2C Fig), indicating that *cyp19a1b* is not implicated in female fate decision. However, all the gonads of the XX *cyp19a1a*^-/-^;*cyp19a1b*^-/-^ double mutants (n = 10) and AI treated-XX *cyp19a1a*^-/-^ mutants (n = 7) developed as complete testes at 45 dah. Whole-mount fluorescence *in situ* hybridization (FISH) result showed that expression of *dmrt1* was detected in the gonads of the XX *cyp19a1a*^-/-^;*cyp19a1b*^-/-^ double mutants at 5 dah as the WT XY (S2D Fig), suggesting that estrogen is involved in female SD.

How the gonad will develop if both *dmrt1* and *cyp19a1a* are mutated is unknown. The *dmrt1*^-/-^;*cyp19a1a*^-/-^ double mutants were established by crossing *dmrt1*^+/-^;*cyp19a1a*^+/-^ double heterozygous males and females, and their gonadal development were analyzed by histological examination. Interestingly, the gonads of the *dmrt1*^-/-^;*cyp19a1a*^-/-^ double mutants developed as either typical ovaries with ovarian cavity and previtellogenic oocytes (17/27, 63%) (named as type I, as demonstrated by 42Sp50 staining) or underdeveloped testes with no germ cells (10/27, 37%) (named as type II, as reflected by Cyp11c1 and a germ cell marker Vasa staining) irrespective of the genetic sex at 60 dah (Fig 1A and 1B). In agreement with the ovary phenotype (type I), the female specific markers *foxl2* (somatic cell), *foxl3* (oogonia), and *bmp15* (oocyte) were detected, while male specific markers *dmrt6*, *creb1b* (spermatocyte) and *cyp11c1* were not detected by RT-PCR, similar to those in the WT XX tilapia at 60 dah (Fig 1C). In addition, we examined the gonad development of the *dmrt1*^-/-^;*cyp19a1a*^-/-^ double mutants at 45 and 120 dah. The ratios of the type I double mutants were 62.5% and 61% at 45 and 120 dah, respectively (S3A–S3C Fig). Furthermore, whole-mount FISH result showed that *foxl3* mRNA was expressed in the gonads of over half (4/7, 57%) of the double mutants at 25 dah. Similar to the WT XY, Amh was expressed in the gonads of the XX/XY *dmrt1*^-/-^;*cyp19a1a*^-/-^ double mutants at 5 dah, indicating the masculinization of somatic cells. Unlike the clear differences in gonadal morphology and *foxl3* expression observed between the type I and type II phenotype in the double mutants at 25 dah, no obvious differences in gonadal morphology and Amh expression were observed between the type I and type II phenotype of the double mutants (n = 7) at 5 dah (Fig 1D). These results excluded the possibility that the underdeveloped testis was transformed from ovary. To our surprise, the Sertoli cell marker Amh and Leydig cell markers 3β-HSD-I and Cyp11c1 were still expressed in the underdeveloped testes of type II double mutants at 120 dah (S3D Fig). These results demonstrate that the gonads of the *dmrt1*^-/-^;*cyp19a1a*^-/-^ double mutants developed as ovaries or underdeveloped testes.

Histological examination showed that the follicles of type I double mutants (n = 7) developed into vitellogenic stage at 150 dah, same as the WT XX ovary (S4A Fig) and XY *dmrt1*^-/-^ ovary [14]. RT-PCR analysis showed that *vtg1*, *vtg2* and *vtg3* mRNAs were expressed in the livers of the type I *dmrt1*^-/-^;*cyp19a1a*^-/-^ double mutants as the XY *dmrt1*^-/-^ and WT XX tilapia at 150 dah (S4B Fig). EIA and UPLC-MS/MS analyses showed that the serum estradiol-17β (E2) level in the double mutants was significantly lower than that of the WT XX fish, but was significantly higher than that of the WT XY fish (S4C Fig). By IF, the Cyp19a1a was expressed in the granulosa and theca cells of the WT XX, XX *cyp19a1b*^-/-^ and XY *dmrt1*^-/-^ mutants and it was disappeared in the *dmrt1*^-/-^;*cyp19a1a*^-/-^ double mutants, whereas Cyp19a1b was only expressed in the theca cells of the WT ovary and it was disappeared in the *cyp19a1b*^-/-^ mutants. It is interesting to note that, in the *dmrt1*^-/-^ single and *dmrt1*^-/-^;*cyp19a1a*^-/-^ double mutants, besides being observed in theca cells, Cyp19a1b was found to be ectopically expressed in the granulosa cells of the ovary at 150 dah (S4D Fig). These studies reveal that Cyp19a1b was up-regulated and ectopically expressed in the context of *dmrt1* loss.

### Gonads of *dmrt1*^-/-^;*cyp19a1a*^-/-^;*cyp19a1b*^-/-^ triple mutants developed as ovaries

**Fig 2:**
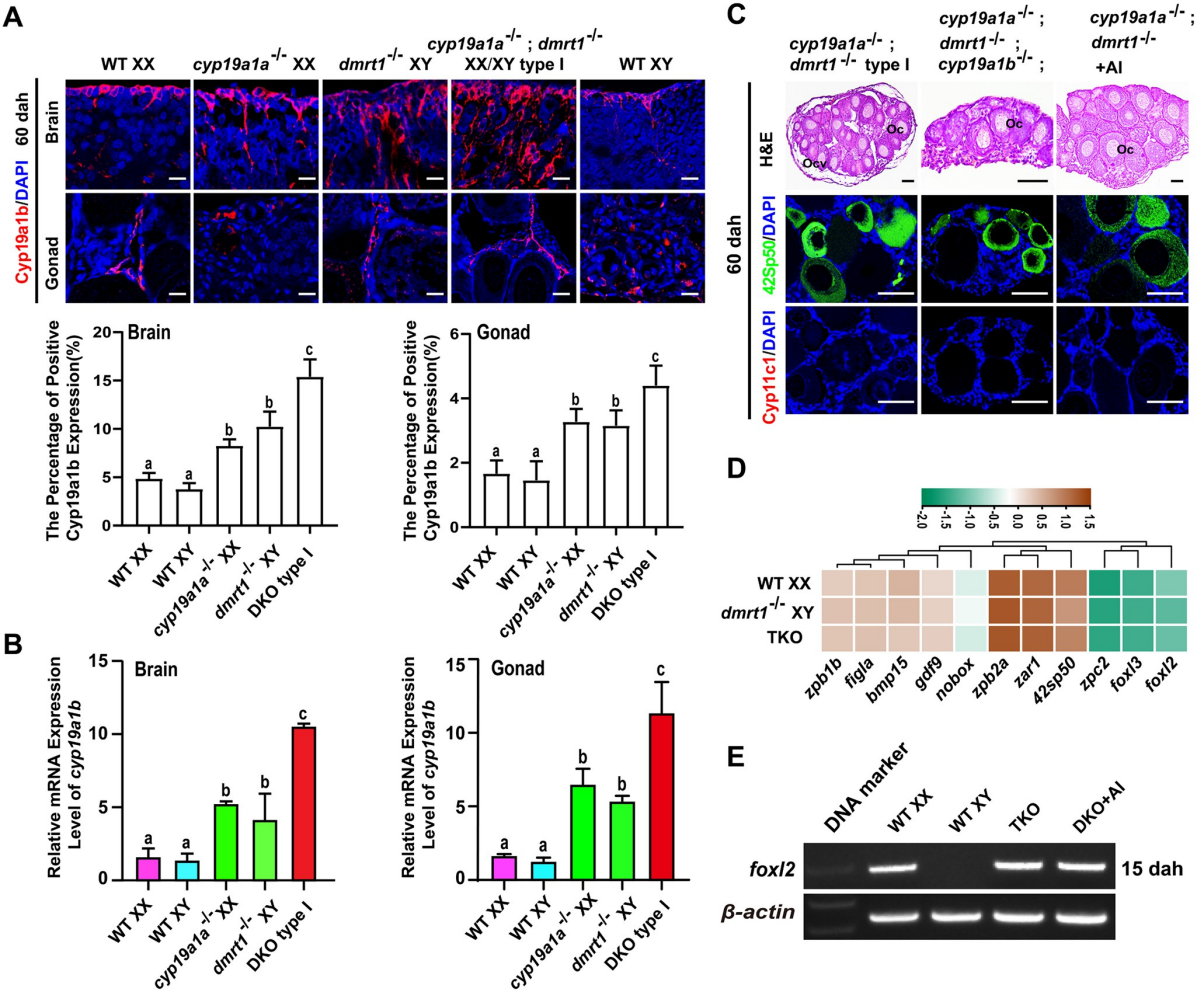
Triple mutation of *dmrt1*;*cyp19a1a*;*cyp19a1b* resulted in ovary development. **(A)** Expression of Cyp19a1b in the brains and gonads of WT XX, WT XY, XY *dmrt1*^-/-^, XX *cyp19a1a*^-/-^ and XX/XY *dmrt1*^-/-^;*cyp19a1a*^-/-^ type I tilapia analyzed by IF at 60 dah. Nuclei were counterstained with DAPI. Quantification of the fluorescence intensity was performed using Image J software. Scale bars = 10 μm. **(B)** Real-time PCR analysis of *cyp19a1b* mRNA expression level in the brains and gonads of WT XX, WT XY, XY *dmrt1*^-/-^, XX *cyp19a1a*^-/-^, and XX/XY *dmrt1*^-/-^;*cyp19a1a*^-/-^ type I tilapia at 60 dah. *β-actin* was used as an internal control. Data were expressed as the mean ± SD of triplicates. Different letters above the error bars indicate statistical differences at *P*<0.05 as determined by one-way ANOVA followed by Tukey test. **(C)** Histological examination of gonads from XX/XY *dmrt1*^-/-^;*cyp19a1a*^-/-^ type I, XX/XY *dmrt1*^-/-^;*cyp19a1a*^-/-^;*cyp19a1b*^-/-^ triple mutants and AI treated-*dmrt1*^-/-^;*cyp19a1a*^-/-^ XX/XY double mutants using H&E staining at 60 dah. Expression of oocyte maker 42Sp50 and Leydig cell marker Cyp11c1 in the gonads of *dmrt1*^-/-^;*cyp19a1a*^-/-^ XX/XY type I, *dmrt1*^-/-^;*cyp19a1a*^-/-^;*cyp19a1b*^-/-^ XX/XY triple mutants and AI treated-*dmrt1*^-/-^;*cyp19a1a*^-/-^ XX/XY double mutants. Nuclei were counterstained with DAPI. Oc, oocyte; Ocv, ovarian cavity. AI, aromatase inhibitor, Letrozole. Scale bars = 40 μm. **(D)** Transcriptome analysis of oogonia and oocyte related genes expression in WT XX, XY *dmrt1*^-/-^ and XX/XY *dmrt1*^-/-^;*cyp19a1a*^-/-^;*cyp19a1b*^-/-^ triple mutants at 60 dah. **(E)** RT-PCR analysis of *foxl2* mRNA expression in the gonads of *dmrt1*^-/-^;*cyp19a1a*^-/-^;*cyp19a1b*^-/-^ triple mutants and AI treated-*dmrt1*^-/-^;*cyp19a1a*^-/-^ double mutants at 15 dah. *β-actin* was used as an internal control. WT, wild-type; dah, days after hatching.

Studies have reported that *cyp19a1b* is expressed in both brain and gonad tissues in fish species [17]. By IF and real-time PCR analyses, up-regulation of Cyp19a1b/*cyp19a1b* expression was detected in the brains and ovaries of the type I *dmrt1*^-/-^;*cyp19a1a*^-/-^ double mutants compared with WT XX, WT XY, *dmrt1*^-/-^ and *cyp19a1a*^-/-^ single mutants at 60 dah (Fig 2A and 2B). To examine the possible contribution of *cyp19a1b* to ovary development in the *dmrt1*^-/-^;*cyp19a1a*^-/-^ double mutants, we established the *dmrt1*^-/-^;*cyp19a1a*^-/-^;*cyp19a1b*^-/-^ triple mutants. The gonads of the triple mutants exhibited ovary phenotype with previtellogenic oocytes as demonstrated by 42Sp50 staining at 60 dah (n = 6). The disappearance of ovarian cavity, which formation depends on estrogen [54], indicated the absence of estrogen in the triple mutants. Similar phenotype was also observed in the AI treated-XX/XY *dmrt1*^-/-^;*cyp19a1a*^-/-^ double mutants. The somatic cells in the gonads of the triple mutants and AI treated-*dmrt1*^-/-^;*cyp19a1a*^-/-^ double mutants were feminized as demonstrated by absence of Cyp11c1 expression (Fig 2C). Transcriptome analysis showed that the expression levels of oogonia and oocyte related genes *foxl3*, *bmp15*, *zp*, *42sp50* and *figla*, *etc*, in the ovaries of triple mutants were similar to those of WT XX ovaries at 60 dah (Fig 2D), which was further validated by real-time PCR (S5A Fig). Interestingly, genes related to gonadal steroidogenesis, including *cyp17a1/2*, *StAR1/2*, and *3β*-*hsd*-*I/II*, showed higher expression levels in the ovaries of the triple mutants compared with those of the WT XX and XY *dmrt1*^-/-^ mutants (S5A Fig). In addition, the number of germ cells in the XX/XY *dmrt1*^-/-^;*cyp19a1a*^-/-^;*cyp19a1b*^-/-^ triple mutants was similar to that of the WT XX, XX/XY *dmrt1*^-/-^ mutants, but different from WT XY and XX *cyp19a1a*^-/-^;*cyp19a1b*^-/-^ double mutants at 15 and 25 dah as revealed by whole-mount IF with Vasa antibody, suggesting a female germ cell number in *dmrt1*^-/-^;*cyp19a1a*^-/-^;*cyp19a1b*^-/-^ triple mutants (S5B Fig). RT-PCR analysis showed that *foxl2* was expressed in the gonads of XX/XY *dmrt1*^-/-^;*cyp19a1a*^-/-^;*cyp19a1b*^-/-^ triple mutants and AI treated-XX/XY *dmrt1*^-/-^;*cyp19a1a*^-/-^ double mutants, but not in the gonads of WT XY at 15 dah (Fig 2E). Hence, the gonads of the *dmrt1*^-/-^;*cyp19a1a*^-/-^;*cyp19a1b*^-/-^ triple mutants developed as ovaries, suggesting that the ovary development of the *dmrt1*^-/-^;*cyp19a1a*^-/-^ double mutants is caused by the absence of *dmrt1*, rather than compensation of estrogen from *cyp19a1b*.

The gonads of the *dmrt1*^-/-^;*cyp19a1a*^-/-^;*cyp19a1b*^-/-^ triple mutants developed into ovaries but with only previtellogenic follicles at 150 dah, different from the type I *dmrt1*^-/-^;*cyp19a1a*^-/-^ double mutants that have vitellogenic follicles in the ovary (S5C Fig). RT-PCR analysis showed the absence of *vtg1*, *vtg2* and *vtg3* mRNAs in the livers of *dmrt1*^-/-^;*cyp19a1a*^-/-^;*cyp19a1b*^-/-^ triple mutants at 150 dah (S5D Fig). The follicles of both type I *dmrt1*^-/-^;*cyp19a1a*^-/-^ double and *dmrt1*^-/-^;*cyp19a1a*^-/-^;*cyp19a1b*^-/-^ triple mutants were degenerated with apoptosis of granulosa and theca cells at 240 dah, as revealed by TUNEL analysis. The gonads of type I *dmrt1*^-/-^;*cyp19a1a*^-/-^ double and *dmrt1*^-/-^;*cyp19a1a*^-/-^;*cyp19a1b*^-/-^ triple mutants developed as underdeveloped testis with no apparent germ cells at 360 dah (S5E Fig). These results suggested that estrogen is required for ovary maintenance even though *dmrt1* is mutated.

### Gonads of *dmrt1*^-/-^;*cyp19a1a*^-/-^;*foxl3*^-/-^ triple mutants eventually developed as ovaries

**Fig 3:**
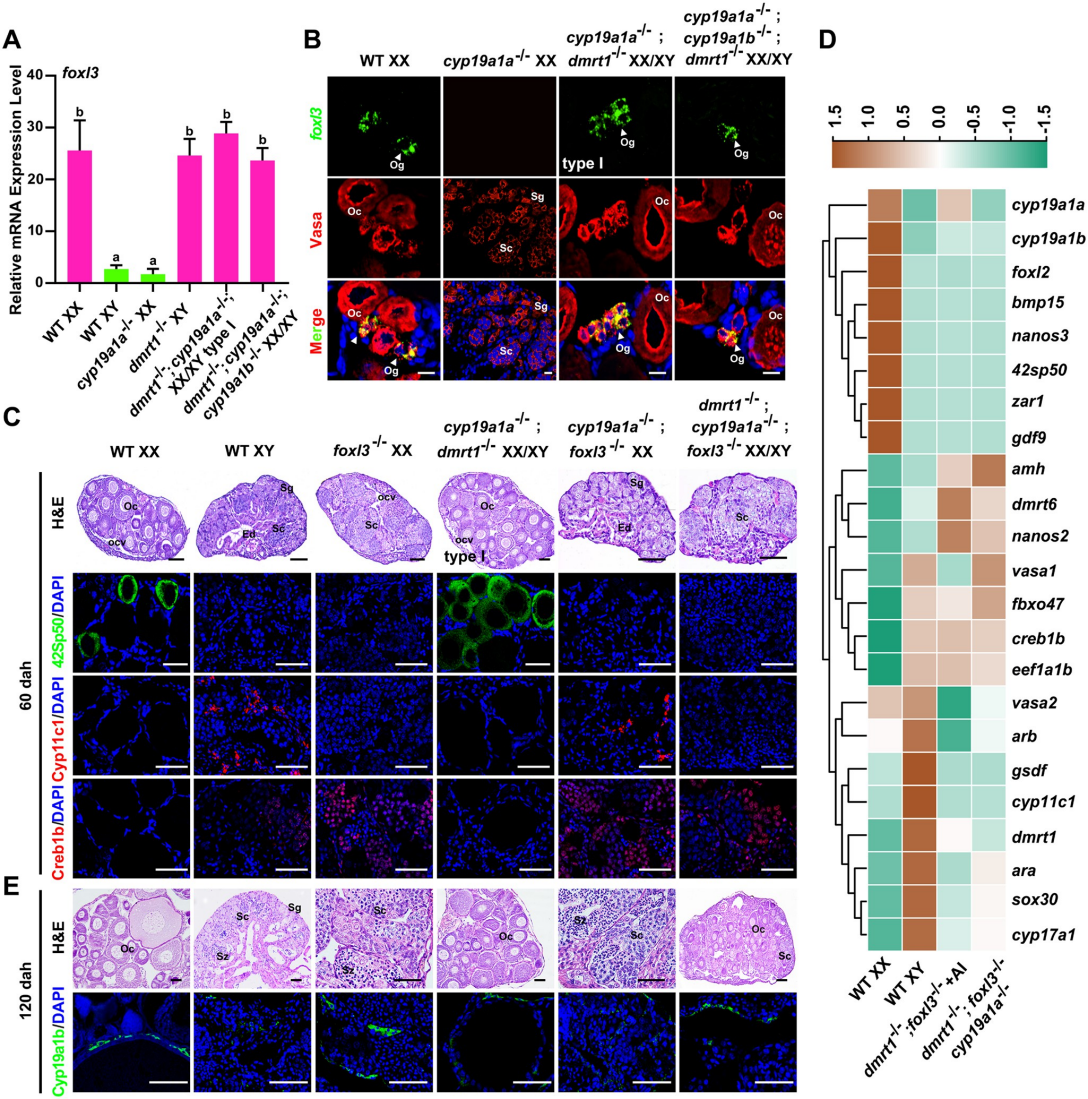
Triple mutation of *dmrt1*;*cyp19a1a*;*foxl3* leads to ovary development eventually. **(A)** Real-time PCR analysis of *foxl3* mRNA expression in the gonads of WT XX, WT XY, XY *dmrt1*^-/-^, XX *cyp19a1a*^-/-^, XX/XY *dmrt1*^-/-^;*cyp19a1a*^-/-^ type I and XX/XY *dmrt1*^-/-^;*cyp19a1a*^-/-^;*cyp19a1b*^-/-^ tilapia at 60 dah. *β-actin* was used as an internal control. Data were expressed as the mean ± SD. Different letters above the error bars indicate statistical differences at *P*<0.05 as determined by one-way ANOVA followed by Tukey test. **(B)** Expression of *foxl3* mRNA (green) in the gonads of WT XX, XX *cyp19a1a*^-/-^ and XX/XY *dmrt1*^-/-^;*cyp19a1a*^-/-^ type I and XX/XY *dmrt1*^-/-^;*cyp19a1a*^-/-^;*cyp19a1b*^-/-^ tilapia by FISH at 60 dah. Germ cells (red) were stained by Vasa antibody staining. Nuclei were counterstained with DAPI. Scale bars = 10 μm. **(C)** Histological examination of gonads from WT XX, WT XY, XX *foxl3*^-/-^, XX/XY *dmrt1*^-/-^;*cyp19a1a*^-/-^ type I, XX *cyp19a1a*^-/-^;*foxl3*^-/-^ and XX/XY *dmrt1*^-/-^;*cyp19a1a*^-/-^;*foxl3*^-/-^ tilapia at 60 dah by H&E staining. Expressions of somatic cell and germ cell markers were analyzed by IF. Nuclei were counterstained with DAPI. Scale bars = 40 μm. **(D)** Transcriptome analysis of germ cell and somatic cell marker genes expression in WT XX, WT XY, AI treated-XX/XY *dmrt1*^-/-^*;foxl3*^-/-^ and XX/XY *dmrt1*^-/-^;*cyp19a1a*^-/-^;*foxl3*^-/-^ tilapia. AI, aromatase inhibitor, Letrozole. **(E)** Histological examination of gonads from WT XX, WT XY, XX *foxl3*^-/-^, XX/XY *dmrt1*^-/-^;*cyp19a1a*^-/-^ type I, XX *cyp19a1a*^-/-^;*foxl3*^-/-^ and XX/XY *dmrt1*^-/-^;*cyp19a1a*^-/-^;*foxl3*^-/-^ tilapia at 120 dah by H&E staining. Expression of Cyp19a1b in gonads of these mutants was analyzed by IF. Nuclei were counterstained with DAPI. Scale bars = 40 μm. Og, oogonia. Oc, oocyte. Ocv, ovarian cavity. Sg, spermatogonia. Sc, spermatocyte. Sz, spermatozoa. Ed, efferent duct. WT, wild-type. dah, days after hatching.

Given that germline expressed *foxl3* is important for female germ cell fate decision [14, 27], we focused on the role of *foxl3* in the ovary development type I *dmrt1*^-/-^;*cyp19a1a*^-/-^ double and *dmrt1*^-/-^;*cyp19a1a*^-/-^;*cyp19a1b*^-/-^ triple mutants. Real-time PCR and FISH analyses showed that *foxl3* mRNA was expressed in the type I *dmrt1*^-/-^;*cyp19a1a*^-/-^ double and *dmrt1*^-/-^;*cyp19a1a*^-/-^;*cyp19a1b*^-/-^ triple mutants, similar to the XY *dmrt1*^-/-^ mutants and WT XX at 60 dah, but different from the XX *cyp19a1a*^-/-^ mutants and WT XY tilapia (Fig 3A and 3B). Double mutation of *cyp19a1a* and *foxl3* in XX tilapia resulted in testis development with normal spermatogenesis as demonstrated by IF using male markers Gsdf, Cyp11c1 and Creb1b, and female markers Cyp19a1a and 42Sp50 (n = 8) at 120 dah (S6 Fig). In addition, in the XX/XY *dmrt1*^-/-^;*cyp19a1a*^-/-^;*foxl3*^-/-^ triple mutants, the gonads displayed testicular morphology with spermatocyte-like cells as demonstrated by presence of Creb1b expression and absence of 42Sp50 expression at 60 dah (n = 4) (Fig 3C). Transcriptome analysis showed that oocyte markers *zar1*, *gdf9*, *nanos3* and *bmp15* were not expressed in the gonads of the *dmrt1*^-/-^;*cyp19a1a*^-/-^;*foxl3*^-/-^ triple mutants at 60 dah, similar to the WT XY fish. In contrast, spermatogenic cell markers *eef1a1b*, *dmrt6*, *fbxo47*, *creb1b* and *sox30* were expressed in the gonads of the *dmrt1*^-/-^;*cyp19a1a*^-/-^;*foxl3*^-/-^ triple mutants compared with the WT XX. Although *foxl2* was not up-regulated in the triple mutants, the somatic cells were overall feminized as reflected by the low expression of male markers *cyp11c1*, *cyp17a1* and *gsdf* compared with the WT XY (Fig 3D), indicating a misexpression of sex specific genes in somatic cells. However, the gonads of the *dmrt1*^-/-^;*cyp19a1a*^-/-^;*foxl3*^-/-^ triple mutants reversed to ovaries with most of the areas occupied by oocytes and small areas occupied by spermatocytes-like cells at 120 dah (n = 3) (Fig 3E). Previously, we reported that the gonads of the *dmrt1*^-/-^;*foxl3*^-/-^ double mutants developed as ovaries [14], but reversed to testicular morphology with spermatocyte-like cells by AI treatment for 60 [14] or 120 days in this study. However, the gonads will spontaneously revert to ovaries with vitellogenic follicles after withdrawal of AI treatment (S7 Fig). These data indicate that even if *cyp19a1a* and *foxl3*, both of which are absolutely essential for female fate, were disrupted, the gonads still developed as ovaries when *dmrt1* was mutated.

### Gonads of *amhy*^−^;*foxl3*^-/-^ and *gsdf*^-/-^;*foxl3*^-/-^ double mutants developed as ovaries but with germ cells in spermatogenesis

**Fig 4:**
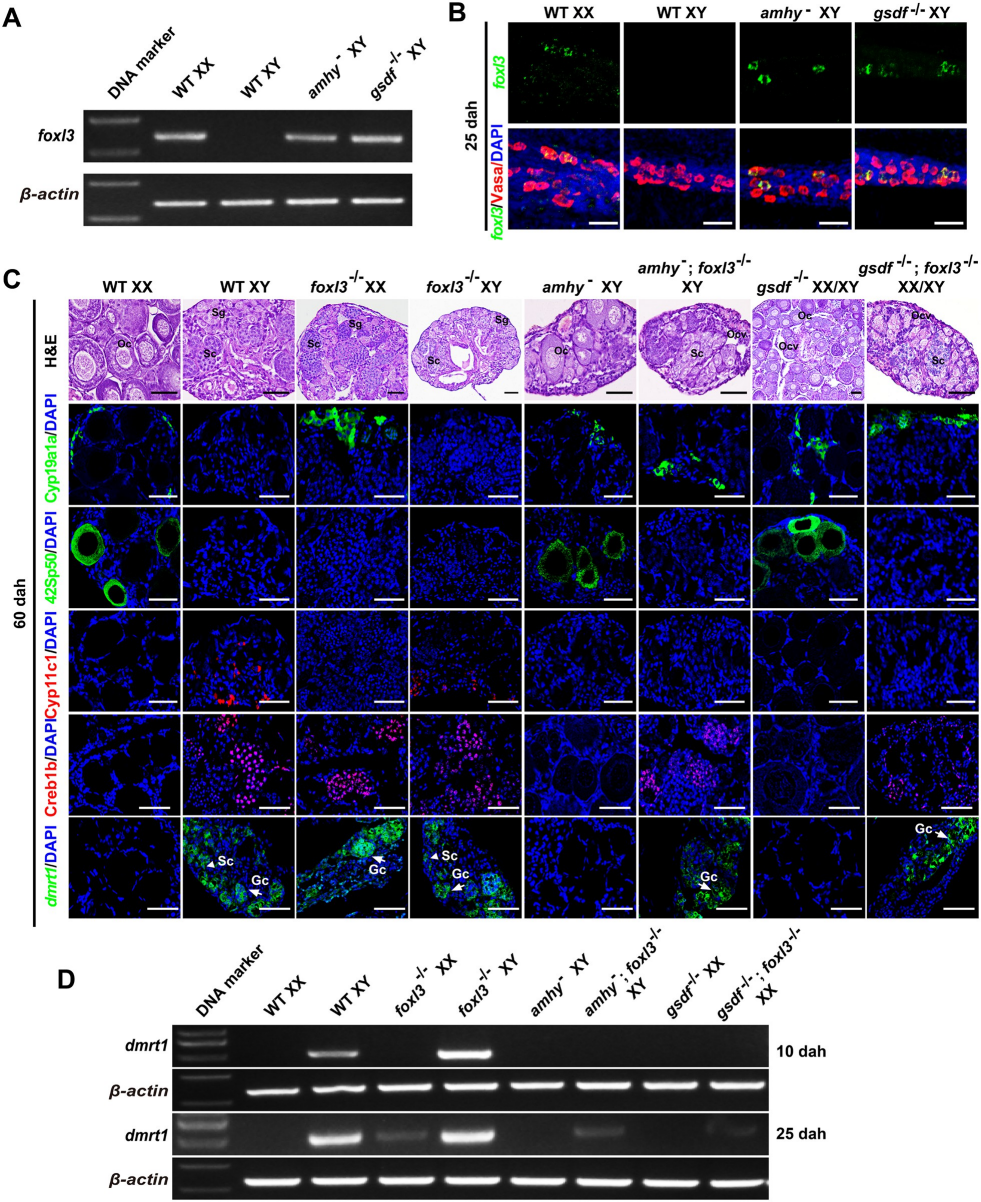
Double mutation of *amhy*;*foxl3* and *gsdf*;*foxl3* leads to ovary development but with germ cells in spermatogenesis. (**A**) RT-PCR analysis of *foxl3* mRNA expression in the gonads of WT XX, WT XY, XY *amhy*^−^ and *gsdf*^-/-^ mutants at 25 dah. *β-actin* was used as an internal control. (**B**) Whole-mount FISH analysis of *foxl3* mRNA (green) expression in the gonads of WT XX, WT XY, XY *amhy*^−^ and *gsdf*^-/-^ at 25 dah. Germ cells (red) were labeled using Vasa antibody. Yellow color indicates the co-expression signals of *foxl3* and Vasa. Scale bars = 40 μm. (**C**) Histological examination of gonads from WT XX, WT XY, XX/XY *foxl3*^-/-^, XY *amhy*^−^, XY *amhy*^−^;*foxl3*^-/-^, XX/XY *gsdf*^-/-^, XX/XY *gsdf*^-/-^;*foxl3*^-/-^ tilapia at 60 dah using H&E staining. Expressions of somatic cell (Cyp19a1a, Cyp11c1) and germ cell (42Sp50, Creb1b) markers were analyzed by IF at 60 dah. FISH was performed to analyze the *dmrt1* mRNA expression in WT XX, WT XY, XX/XY *foxl3*^-/-^, XY *amhy*^−^, XY *amhy*^−^;*foxl3*^-/-^, XX/XY *gsdf*^-/-^, XX/XY *gsdf*^-/-^;*foxl3*^-/-^ tilapia at 60 dah. Nuclei were counterstained with DAPI. Arrow and arrowhead indicates germ cell (Gc) and somatic cell (Sc), respectively. Oc, oocyte. Ocv, ovarian cavity. Sg, spermatogonia. Sc, spermatocyte. Scale bars = 40 μm. (**D**) RT-PCR analysis of *dmrt1* mRNA expression in the gonads of WT XX, WT XY, XX *foxl3*^-/-^, XY *foxl3*^-/-^, XY *amhy*^−^, XX *gsdf*^-/-^, XY *amhy*^−^;*foxl3*^-/-^ and XX *gsdf*^-/-^;*foxl3*^-/-^ tilapia at 10 and 25 dah. Dmrt1 was found to be expressed in the gonads of the XY *gsdf*^-/-^ mutants but not in the XX *gsdf*^-/-^ mutants at 10 dah [12]. Therefore, *dmrt1* expression at 10 dah was examined to investigate whether germ cells were masculinized in the gonads of the XX *gsdf*^-/-^;*foxl3*^-/-^ double mutants at this stage using the XX *gsdf*^-/-^ mutants as control. *β-actin* was used as an internal control. WT, wild-type. dah, days after hatching.

Previous studies showed that mutation of *amhy* or *gsdf* resulted in both somatic and germ cell SR in XY tilapia [9,12]. RT-PCR and whole-mount FISH analyses showed that *foxl3* mRNA was specifically expressed in the germ cells of XY *amhy*^−^ and *gsdf*^-/-^ mutants as the WT XX at 25 dah (Fig 4A and 4B). It is unclear how the gonad will be developed if both *amhy* or *gsdf* and *foxl3* are lost in tilapia. To answer this question, we established the XY *amhy*^−^;*foxl3*^-/-^ double mutants by crossing XY *amhy*^−^;*foxl3*^+/-^ phenotypic females with XY *foxl3*^-/-^ phenotypic males and XX/XY *gsdf*^-/-^;*foxl3*^-/-^ double mutants by crossing the *gsdf*^+/-^;*foxl3*^+/-^ double heterozygous males and females. Consistent with our previous studies [9,12,14], all the gonads of the XY *amhy*^−^ and XX/XY *gsdf*^-/-^ tilapia developed as ovaries and mutation of *foxl3* in XX tilapia resulted in masculinization of germ cells in ovary at 60 dah. The gonads of the XY *foxl3*^-/-^ mutants developed as testes as those of the WT XY fish. However, only somatic cells but not germ cells were feminized in the XY *amhy*^−^;*foxl3*^-/-^ (n = 6) and XX/XY *gsdf*^-/-^;*foxl3*^-/-^ (n = 8) double mutants at 60 dah as demonstrated by IF using female Cyp19a1a and 42Sp50, male Cyp11c1 and Creb1b markers. Importantly, whole-mount FISH analysis showed that *dmrt1* was mainly expressed in the germ cells of the *amhy*^−^;*foxl3*^-/-^ and *gsdf*^-/-^;*foxl3*^-/-^ double mutants at 60 dah (Fig 4C), as reported in the *foxl3*^-/-^ single mutant [14]. Similarly, only somatic cells but not germ cells were feminized in the XY *amhy*^−^;*foxl3*^-/-^ (n = 4) and XX/XY *gsdf*^-/-^;*foxl3*^-/-^ (n = 5) double mutants at 120 dah as demonstrated by IF using female Cyp19a1a and 42Sp50, male Cyp11c1, Gsdf and Creb1b markers. Sox30, which is mainly detected in spermatozoa in testis [55], was detected in the gonads of the *amhy*^−^;*foxl3*^-/-^ and *gsdf*^-/-^;*foxl3*^-/-^ double mutants at 120 dah (S8A Fig). These data suggested that in the presence of *dmrt1*, even if *amhy* or *gsdf* was mutated, germ cells still entered into spermatogenesis in the absence of *foxl3*.

To examine the early gonad development in *amhy*^−^;*foxl3*^-/-^ and *gsdf*^-/-^;*foxl3*^-/-^ double mutants, we performed whole-mount IF with Vasa antibody to observe the germ cells. The results showed that the number of germ cells in XX *foxl3*^-/-^ single, XY *amhy*^−^;*foxl3*^-/-^ and XX/XY *gsdf*^-/-^;*foxl3*^-/-^ double mutants was similar to that of the WT XX, XY *amhy*^−^ and XX/XY *gsdf*^-/-^ tilapia, but different from that of the WT XY tilapia at 15 dah (S8B Fig). In our previous study, Dmrt1 was found to be expressed in the gonads of the XY *gsdf*^-/-^ mutants but not in the XX *gsdf*^-/-^ mutants at 10 dah [12]. Therefore, *dmrt1* expression at 10 dah was examined to investigate whether germ cells were masculinized in the gonads of the XX *gsdf*^-/-^;*foxl3*^-/-^ double mutants at this stage using the XX *gsdf*^-/-^ mutants as control. RT-PCR analysis showed that *dmrt1* was not expressed in the gonads of XX *foxl3*^-/-^, XX *gsdf*^-/-^, XY *amhy*^−^;*foxl3*^-/-^ and XX *gsdf*^-/-^;*foxl3*^-/-^ double mutants at 10 dah as the WT XX tilapia. However, expression of *dmrt1* was observed in the gonads of XX *foxl3*^-/-^ single, XY *amhy*^−^;*foxl3*^-/-^ and XX *gsdf*^-/-^;*foxl3*^-/-^ double mutants at 25 dah (Fig 4D). These results indicated that the female fate of germ cells in *foxl3* single, *amhy*;*foxl3* and *gsdf*;*foxl3* double mutants could not be maintained due to up-regulation of *dmrt1*.

### Gonads of *amhy*^−^;*cyp19a1a*^-/-^;*cyp19a1b*^-/-^ and *gsdf*^-/-^;*cyp19a1a*^-/-^;*cyp19a1b*^-/-^ triple mutants developed as functional testes

**Fig 5:**
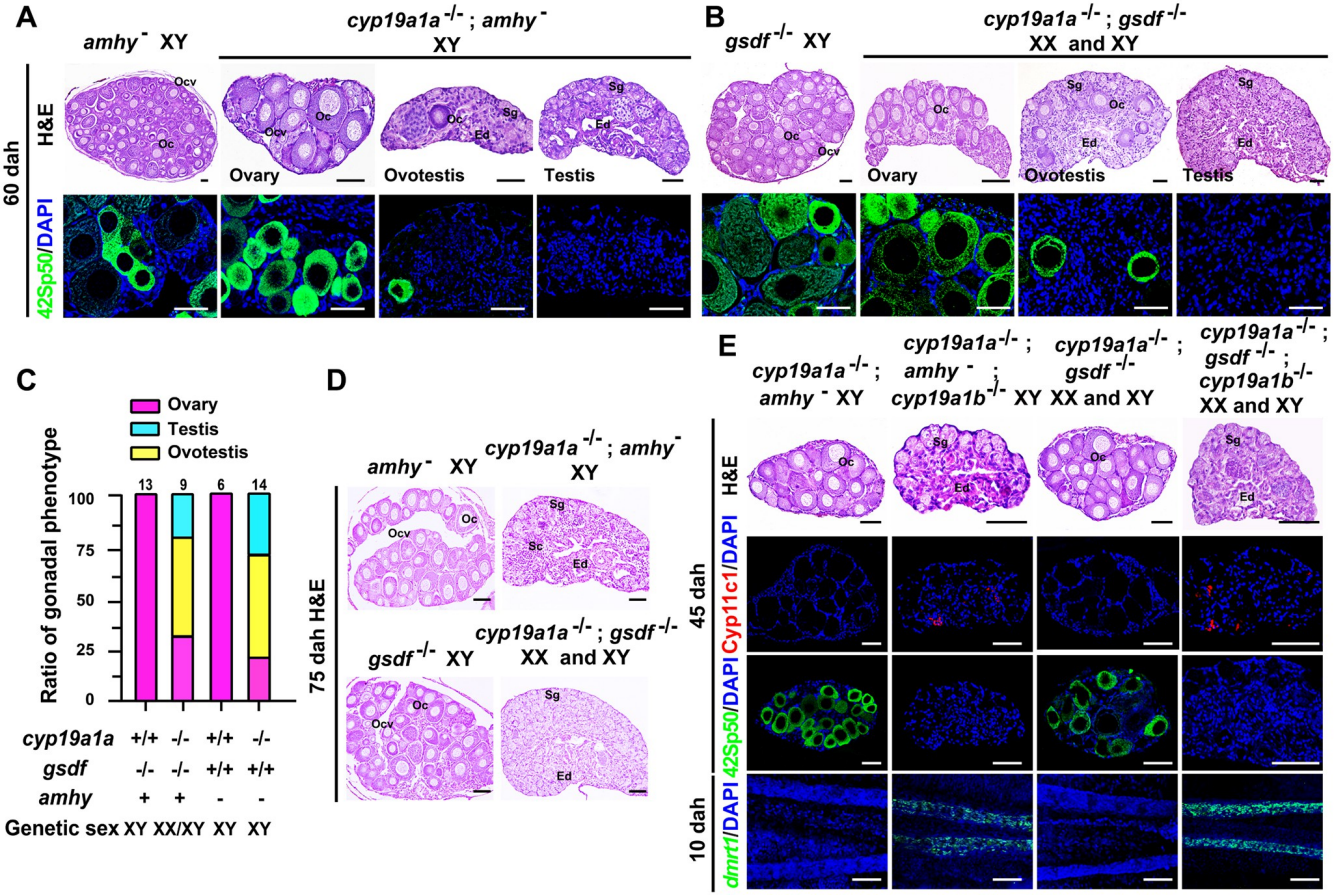
Double mutation of *amhy*;*cyp19a1a*, *gsdf*;*cyp19a1a* and triple mutation of *amhy*;*cyp19a1a*;*cyp19a1b* and *gsdf*;*cyp19a1a*;*cyp19a1b* resulted in functional testis development. **(A, B)** Histological examination of gonads from XY *amhy*^−^, XY *amhy*^−^;*cyp19a1a*^-/-^, XY *gsdf*^-/-^, XX/XY *gsdf*^-/-^;*cyp19a1a*^-/-^ tilapia at 60 dah using H&E staining. Expression of oocyte marker 42Sp50 was analyzed by IF at 60 dah. Nuclei were counterstained with DAPI. Scale bars = 40 μm. **(C)** Sex ratios in XY *amhy*^−^, XY *amhy*^−^;*cyp19a1a*^-/-^, XY *gsdf*^-/-^, XX/XY *gsdf*^-/-^;*cyp19a1a*^-/-^ tilapia at 60 dah. **(D)** Histological examination of gonads from XY *amhy*^−^, XY *amhy*^−^;*cyp19a1a*^-/-^, XY *gsdf*^-/-^, XX/XY *gsdf*^-/-^;*cyp19a1a*^-/-^ tilapia at 75 dah by H&E staining. Scale bars = 50 μm. **(E)** Histological examination of gonads from XY *amhy*^−^;*cyp19a1a*^-/-^, XY *amhy*^−^;*cyp19a1a*^-/-^;*cyp19a1b*^-/-^, XX/XY *gsdf*^-/-^;*cyp19a1a*^-/-^ and XX/XY *gsdf*^-/-^;*cyp19a1a*^-/-^;*cyp19a1b*^-/-^ tilapia at 45 dah by H&E staining. Expressions of 42Sp50 and Leydig cell marker Cyp11c1 were analyzed by IF at 45 dah. Whole-mount FISH analysis of the *dmrt1* mRNA expression in the gonads of these mutants at 10 dah. Nuclei were counterstained with DAPI. Scale bars = 50 μm. Oc, oocyte. Ocv, ovarian cavity. Sg, spermatogonia. Ed, efferent duct. dah, days after hatching.

Over half of *dmrt1*^-/-^;*cyp19a1a*^-/-^ double mutants developed as ovaries in this study, while double mutation of *amhy* and *cyp19a1a* resulted in testicular development at 90 dpf [43]. It is interesting to know whether the gonads of the XY *amhy*^−^;*cyp19a1a*^-/-^ and XX/XY *gsdf*^-/-^;*cyp19a1a*^-/-^ double mutants will also develop into ovaries at early stage. It was found that all the gonads of the XY *amhy*^−^;*cyp19a1a*^-/-^ (n = 5) and XX/XY *gsdf*^-/-^;*cyp19a1a*^-/-^ (n = 7) double mutants developed as ovaries indistinguishable from the XY *amhy*^−^ and *gsdf*^-/-^ single mutants ovaries at 45 dah (S9A Fig). Subsequently, the gonads of the XY *amhy*^−^;*cyp19a1a*^-/-^ (10/14, 71%) and XX/XY *gsdf*^-/-^;*cyp19a1a*^-/-^ (7/9, 78%) double mutants developed as ovaries or ovotestes with dispersed oocytes marked by 42Sp50 expression at 60 dah. The gonads of the remaining XY *amhy*^−^;*cyp19a1a*^-/-^ (4/14, 29%) and XX/XY *gsdf*^-/-^;*cyp19a1a*^-/-^ (2/9, 22%) double mutants developed as testes at 60 dah (Fig 5A–5C). Further, all the gonads of the XY *amhy*^−^;*cyp19a1a*^-/-^ (n = 6) and XX/XY *gsdf*^-/-^;*cyp19a1a*^-/-^ (n = 9) double mutants developed as testes at 75 dah (Fig 5D). These observations demonstrated that the gonads of the *amhy*^−^;*cyp19a1a*^-/-^ and *gsdf*^-/-^;*cyp19a1a*^-/-^ double mutants developed as ovaries at early stage, but reverted to testes in the later stages.

As expected, all the gonads of the AI treated-XY *amhy*^−^;*cyp19a1a*^-/-^ (n = 9) and -XX/XY *gsdf*^-/-^;*cyp19a1a*^-/-^ (n = 8) double mutants developed as testes at 45 dah as demonstrated by Cyp11c1 and 42Sp50 staining (S9B Fig). Higher expression of Cyp19a1b/*cyp19a1b* was observed in these two double mutants compared with the *amhy*^−^ and *gsdf*^-/-^ single mutants at 45 dah by IF and real-time PCR analyses (S9C and S9D Fig). To further assess the compensation of *cyp19a1b* in early ovary development in the two double mutants, we established the XY *amhy*^−^;*cyp19a1a*^-/-^;*cyp19a1b*^-/-^ and XX/XY *gsdf*^-/-^;*cyp19a1a*^-/-^;*cyp19a1b*^-/-^ triple mutants. All the gonads of these two triple mutants developed as testes at 45 dah as demonstrated by presence of Cyp11c1 expression, but absence of 42Sp50 expression. In addition, whole-mount FISH analysis revealed that *dmrt1* mRNA was detected in the gonads of these two triple mutants at 10 dah (Fig 5E). These two triple mutants exhibited testicular development with all types of spermatogenic cells at 120 dah and finally were fertile at 180 dah in fertility assay (S9E and S9F Fig). These data suggested that loss of both *cyp19a1a* and *cyp19a1b* rescued the male-to-female SR of *amhy* and *gsdf* mutants.

### Gonads of *amhy*^−^;*foxl2*^KD^ and *gsdf*^-/-^;*foxl2*^KD^ double mutants developed as functional testes

**Fig 6:**
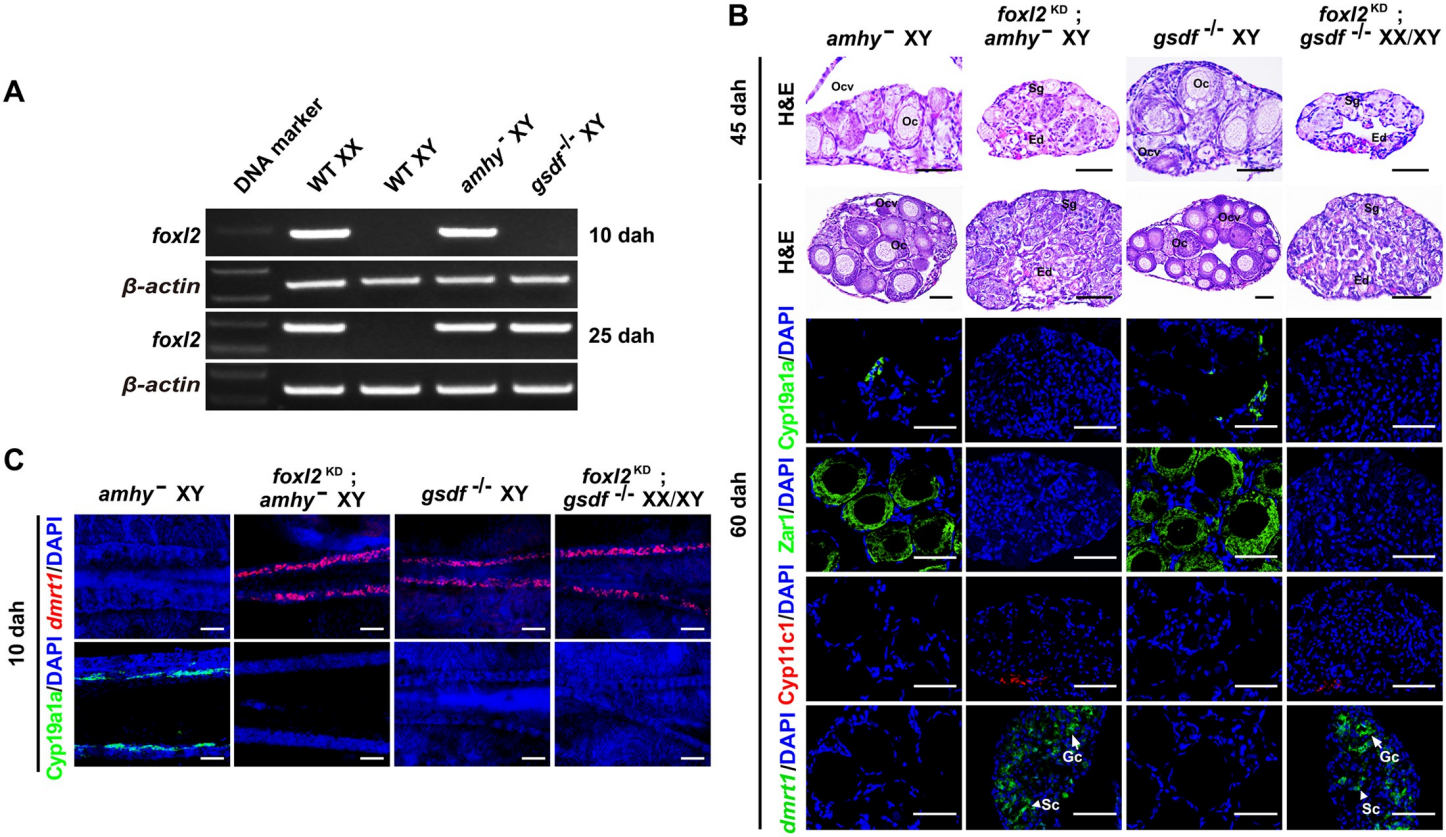
Double mutation of *amhy*;*foxl2* and *gsdf*;*foxl2* leads to functional testis development. (A) RT-PCR analysis of *foxl2* mRNA expression in the gonads of WT XX, WT XY, XY *amhy*^−^ and *gsdf*^-/-^ at 10 and 25 dah. *β-actin* was used as an internal control. (**B**) Histological examination of gonads from XY *amhy*^−^, XY *amhy*^−^;*foxl2*^KD^, XY *gsdf*^-/-^, XX/XY *gsdf*^-/-^;*foxl2*^KD^ tilapia at 45 and 60 dah by H&E staining. Expressions of somatic cell (Cyp19a1a, Cyp11c1) and oocyte (Zar1) markers were analyzed by IF at 60 dah. Expression of *dmrt1* mRNA was analyzed by FISH at 60 dah. Arrow and arrowhead indicates germ cell (Gc) and somatic cell (Sc), respectively. Scale bars = 40 μm. (**C**) Detection of *dmrt1* and Cyp19a1a expressions in the gonads of XY *amhy*^−^, XY *amhy*^−^;*foxl2*^KD^, XY *gsdf*^-/-^, XX/XY *gsdf*^-/-^;*foxl2*^KD^ tilapia at 10 dah by whole-mount FISH and IF, respectively. Scale bars = 40 μm. Nuclei were counterstained with DAPI. Oc, oocyte. Ocv, ovarian cavity. Sg, spermatogonia. Sc, spermatocyte. Sz, spermatozoa. Ed, efferent duct. KD, knockdown. WT, wild-type. dah, days after hatching.

RT-PCR analysis showed that *foxl2* mRNA was highly expressed in the gonads of XY *amhy*^−^ but not XY *gsdf*^-/-^ mutants at 10 dah. Its expression was detected in the gonads of both XY *amhy*^−^ and *gsdf*^-/-^ mutants at 25 dah (Fig 6A). To study whether disruption of *foxl2* can rescue the SR in XY *amhy*^−^ and *gsdf*^-/-^ mutants, we injected *foxl2* TALEN mRNA in the embryos of *amhy*^−^ and *gsdf*^-/-^ mutants to knockdown (KD) its expression (S10A Fig). Consistent with our previous studies [14,56], the gonads of the F0 *foxl2* XX mutants with high mutation rate, named as *foxl2*^KD^, developed as testes at 60 dah (S10B Fig). Different from the ovary phenotype of XY *amhy*^-^ and *gsdf*^-/-^ single mutants, testis development was observed in the XY *amhy*^−^;*foxl2*^KD^ (n = 14 for 60 dah, n = 5 for 45 dah) and XX/XY *gsdf*^-/-^;*foxl2*^KD^ (n = 10 for 60 dah, n = 7 for 45 dah) double mutants, which was demonstrated by the presence of Cyp11c1 and *dmrt1* expressions, and absence of female specific Zar1 (oocyte) and Cyp19a1a expressions (Figs 6B, S10C and S10D). In addition, the testes of these two double mutants were filled with all types of spermatogenic cells at 120 dah (S10E Fig). Furthermore, whole-mount FISH analysis showed that *dmrt1* mRNA was expressed in the gonads of XY *amhy*^−^;*foxl2*^KD^ double mutants, different from the XY *amhy*^−^ mutants at 10 dah. *dmrt1* mRNA was expressed in the gonads of XY *gsdf*^-/-^;*foxl2*^KD^ double mutants, same as the XY *gsdf*^-/-^ mutants at 10 dah. Consistent with our previous studies [12,43], whole-mount IF analysis showed that Cyp19a1a expression was detected in the gonads of XY *amhy*^−^ but not XY *gsdf*^-/-^ fish at 10 dah. However, Cyp19a1a was not expressed in the gonads of both XY *amhy*^−^;*foxl2*^KD^ and XX/XY *gsdf*^-/-^;*foxl2*^KD^ double mutants at 10 dah (Fig 6C). Thus, disruption of female pathway gene *foxl2* can completely rescue the male-to-female SR of *amhy* and *gsdf* mutants.

## Discussion

The gonadal fate is controlled and maintained by the antagonistic roles between male and female pathway genes. In recent years, the *amhy*, *dmrt1*, *gsdf* as male pathway genes and *foxl2*, *cyp19a1a*, *foxl3* as female pathway genes have been identified in tilapia, which provide a good model to elucidate genetic interactions between male and female pathway genes in controlling gonadal fate. Previous work from our group demonstrated that the gonads of the *dmrt1*;*foxl3* double mutants develop as ovaries and the gonads of the *dmrt1*;*foxl2* double mutants develop as dysgenesis testes with no germ cells [14]. In this study, we found that the gonads of 4 double mutants, including *amhy*;*cyp19a1a*, *gsdf*;*cyp19a1a*, *amhy*;*foxl2*, *gsdf*;*foxl2* and 2 triple mutants, *amhy*;*cyp19a1a*;*cyp19a1b* and *gsdf*;*cyp19a1a*;*cyp19a1b*, develop as functional testes, and the gonads of 2 double mutants, *amhy*;*foxl3* and *gsdf*;*foxl3*, develop as ovaries but with spermatogenesis. In contrast, the gonads of the *dmrt1*;*cyp19a1a* double mutants (type I), *dmrt1*;*cyp19a1a*;*cyp19a1b* and *dmrt1*;*cyp19a1a*;*foxl3* triple mutants develop as ovaries. An intriguing finding of this study is that mutation of the female pathway genes *foxl3*, *foxl2*, *cyp19a1a* partially or completely rescues the SR in *amhy* and *gsdf* but not *dmrt1* mutants. In line with this, disruption of estrogen synthesis by AI treatment rescues the SR in *amhy* and *gsdf* but not *dmrt1* mutants. Collectively, our results comprehensively reveal epistatic interactions of male and female pathway genes identified in controlling gonadal fate, and suggest that *dmrt1* is the only male pathway gene tested indispensable for SD and functional testis development in tilapia.

### Once *dmrt1* was mutated, the gonad could not be rescued to a functional testis by mutation of any female pathway gene

It is well accepted that estrogen is essential for female sexual fate decision and maintenance in non-mammals [57–59]. However, this view is challenged by recent studies in zebrafish, tilapia and even in chicken that the ovary of *dmrt1* mutants cannot be rescued into testis by mutation of *cyp19a1a* or/and estrogen receptor *esr1*/*esr2a*/*2b* or AI treatment [14,32,39,40]. Similarly, AI treatment fails to induce female-to-male SR in XX spotted scat (*Scatophagus argus*) which lacks the functional *dmrt1* copy (*dmrt1* is located on the sex chromosome, and the X-linked *dmrt1b* is truncated) [58]. In turtle, the gonads are feminized even in male promoting temperature after *dmrt1* knockdown [33]. In line with this, in this study, we provide new data that the gonads of over half of *dmrt1*;*cyp19a1a* double mutants, all *dmrt1*;*cyp19a1a*;*cyp19a1b* triple mutants and AI treated-*dmrt1*;*cyp19a1a* double mutants develop into ovaries in tilapia. These results provide additional evidences to support the conclusion that SR induced by blockage of E2 synthesis is dependent on Dmrt1 [14]. If *dmrt1* is mutated, the gonad cannot be rescued to testis by disruption of *cyp19a1a* or AI treatment.

*foxl3* is identified as a crucial factor for feminizing germ cell in medaka, tilapia and zebrafish [14,27,28]. Loss of *dmrt1* induces *cyp19a1a* and *foxl3* expression in XY tilapia [14]. In the present study, loss of *cyp19a1a* or *cyp19a1a*;*cyp19a1b* disrupted *foxl3* expression in XX tilapia, indicating that *foxl3* expression is dependent on the presence of E2. However, *foxl3* is expressed in the ovaries of *dmrt1*;*cyp19a1a*;*cyp19a1b* triple mutants, suggesting that *foxl3* expression can be independent of E2 when *dmrt1* is absent. Loss of both *cyp19a1a* and *foxl3* in XX tilapia leads to testicular development, which agrees with our previous study [14], but different from the medaka that AI fails to disrupt oocyte formation in *foxl3* mutants [27]. However, the gonads of the *dmrt1*;*foxl3* double, *dmrt1*;*cyp19a1a*;*foxl3* triple and AI treated-*dmrt1*;*foxl3* double mutants eventually developed as ovaries in XX and XY tilapia. Therefore, up-regulation of *cyp19a1a* and *foxl3* in *dmrt1* mutants is the consequence, not the cause, for the male-to-female SR in *dmrt1* mutants. These results suggested that both *cyp19a1a* and *foxl3* are not essential for female fate in the genetic context of *dmrt1* deletion.

In mouse, somatic cells are observed to express Gata4 and Sox9 (Sertoli cell markers) when both *Dmrt1* and *Foxl2* are conditionally knockout in Sertoli cells [60], indicating the mansculization of somatic cells in the double knockout. The somatic cells are also masculinized in the gonads of XX/XY *dmrt1*;*foxl2* double mutant tilapia [14]. Consistently, the somatic cells in the gonads of less than half of XX/XY *dmrt1*;*cyp19a1a* double mutants are masculinized in the present study. These results suggest that *dmrt1* is dispensable for male somatic cells maintenance when its antagonist *foxl2* is mutated in vertebrates.

Besides its role in SD, Dmrt1 is also critical for male germ cell development and survival in vertebrates. In mouse, conditional knockout of *Dmrt1* in XY germ cells leads to apoptosis [61]. In zebrafish, some *dmrt1* single mutants develop into dysgenesis testes with no germ cells [34,40]. Loss of *rbpms2a*;*rbpms2b* or *bmp15* results in testis development [62,63], while triple mutation of *dmrt1*;*rbpms2a*;*rbpms2b* and double mutation of *dmrt1*;*bmp15* result in underdeveloped testis and sterility in adult zebrafish [39]. In tilapia, less than half of *dmrt1*;*cyp19a1a* and all the *dmrt1;foxl2* double mutants have underdeveloped testis with no germ cells [14]. These studies reveal that even though the *dmrt1* mutants are rescued to males, the germ cells cannot be maintained in male somatic environment due to lack of *dmrt1*. The molecular mechanism underlying Dmrt1 in maintaining male germ cells deserves further investigations. In contrast, *dmrt1* is dispensable for female germ cells in fish, as the ovaries of *dmrt1* mutants contain germ cells in zebrafish [34,40], XX tilapia as well as sex reversed XY medaka, XY tilapia [14,35]. The germ cells in the ovaries of *dmrt1* mutants in chickens and rabbits cannot undergo meiosis and folliculogenesis [31,32], but complete meiosis and folliculogenesis have been observed in the ovaries of *dmrt1* mutants in zebrafish, medaka and tilapia, irrespective of their genetics sex (sex reversed or not). The *dmrt1* female mutant is fertile in medaka and zebrafish [34,35,40], while its fertility in other fish remains to be investigated.

Overall, it is plausible that as long as *dmrt1* is mutated, the mutant gonads could not be rescued to functional testes by mutation of any female pathway gene identified so far.

### Once *dmrt1* was present, sex reversal caused by mutation of its upstream and downstream male pathway gene could be rescued

Mutation of *amhy* or *gsdf* resulted in up-regulation of *foxl2* and *cyp19a1a*/Cyp19a1a expressions and male-to-female SR in tilapia [9,12], which can be rescued to functional testis by AI treatment [43,64]. Consistently, we found that the gonads of *gsdf*;*cyp19a1a*, *amhy*;*cyp19a1a*, *amhy*;*foxl2*, *gsdf*;*foxl2* double and *amhy*;*cyp19a1a*;*cyp19a1b*, *gsdf*;*cyp19a1a*;*cyp19a1b* triple mutants developed into functional testes. These results further suggested that induced *cyp19a1a* and *foxl2* expression is responsible for driving the SR in *amhy* and *gsdf* mutants. The primary role of TGF-β signal (*amhy* and *gsdf*) is to repress female pathway genes, which is supported by the studies in tilapia and gibel carp that *amhy* and *gsdf* inhibited *cyp19a1a* transcription [43,44]. It has been demonstrated that expression of *dmrt1* is up-regulated in XX *cyp19a1a* and *foxl2* mutants and AI treated-XX tilapia [13,48], but is down-regulated in XY *amhy* and *gsdf* mutants [9,12]. However, *dmrt1* is expressed in the gonads of the *amhy*;*foxl2*, *gsdf*;*foxl2* double and *amhy*;*cyp19a1a*;*cyp19a1b*, *gsdf*;*cyp19a1a*;*cyp19a1b* triple mutants at 10 dah. On the other hand, the gonads of *dmrt1*;*cyp19a1a*;*cyp19a1b* triple mutants developed as ovaries due to loss of *dmrt1*. Based on these results, we speculate that up- and down-regulation of Dmrt1 is the cause, instead of the consequence, of the testicular and ovarian development in these mutants, respectively. Finally, studies have shown that *gsdf* functions downstream of *dmrt1* because the *gsdf* transcription is directly activated by Dmrt1 in fish species, including tilapia, spotted scat and gibel carp [12,44,65]. Overall, the SR caused by mutation of male pathway genes other than *dmrt1*, including its upstream MSD gene *amhy* and downstream *gsdf*, could be rescued to functional testis by either mutation of the female pathway gene or AI treatment to induce Dmrt1 expression.

Single mutation of *amhy*, *dmrt1* and *gsdf* results in ovary development in XY tilapia [9,12,14]. By contrast, the gonads of both XX/XY *dmrt1*;*foxl2*, *gsdf*;*foxl2* double mutants and XY *amhy*;*foxl2* double mutants developed as testes. These results suggest that testis development is dependent on *amhy*, *dmrt1* and *gsdf* in the presence of *foxl2*, but independent of *amhy*, *dmrt1* and *gsdf* in the absence of *foxl2*. There might be an unknown gene that triggers the testis development in these double mutants. In other words, *foxl2* is essential for female fate decision in the absence of *amhy*, *dmrt1* and *gsdf*.

Recently, we have demonstrated that mutation of *amhy* and *gsdf* results in down-regulation of *dmrt1* expression in XY tilapia [9,12], while mutation of *foxl3* leads to spermatogenesis in the ovarian environment in XX tilapia due to up-regulation of *dmrt1* in germ cells [14]. In the present study, spermatogenesis in ovarian somatic environment and up-regulation of *dmrt1* in germ cells was observed in XY *amhy*;*foxl3* and *gsdf*;*foxl3* double mutants, same as the XX *foxl3* single mutants. These results further support the notion that up-regulation of *dmrt1* is the cause, instead of the consequence, of the spermatogenesis in the ovarian environment, and reveal close crosstalk between somatic and germ cells during SD. Antagonism of Dmrt1 and Foxl3 in germ cell determines its sexual fate and their expressions rely on absence or presence of E2. If *foxl3* is absent or mutated, E2 is unable to feminize the germ cells. This is probably supported by the fact that *foxl3* gene is lost in the genome of eutherian mammals in which SD is not affected by estrogen. Importantly, sperm was produced in the XX *foxl3* mutant of medaka [27] and tilapia (S11 Fig). Therefore, even in the female somatic cell environment, up-regulation of *dmrt1* in germ cells produces functional sperm. Our data strongly suggest that germline sexual fate is acquired by *foxl3* in females and by *dmrt1* in males in tilapia.

### Indispensable role of Dmrt1 in male might be attributed to its expression in both somatic and germ cell

**Fig 7:**
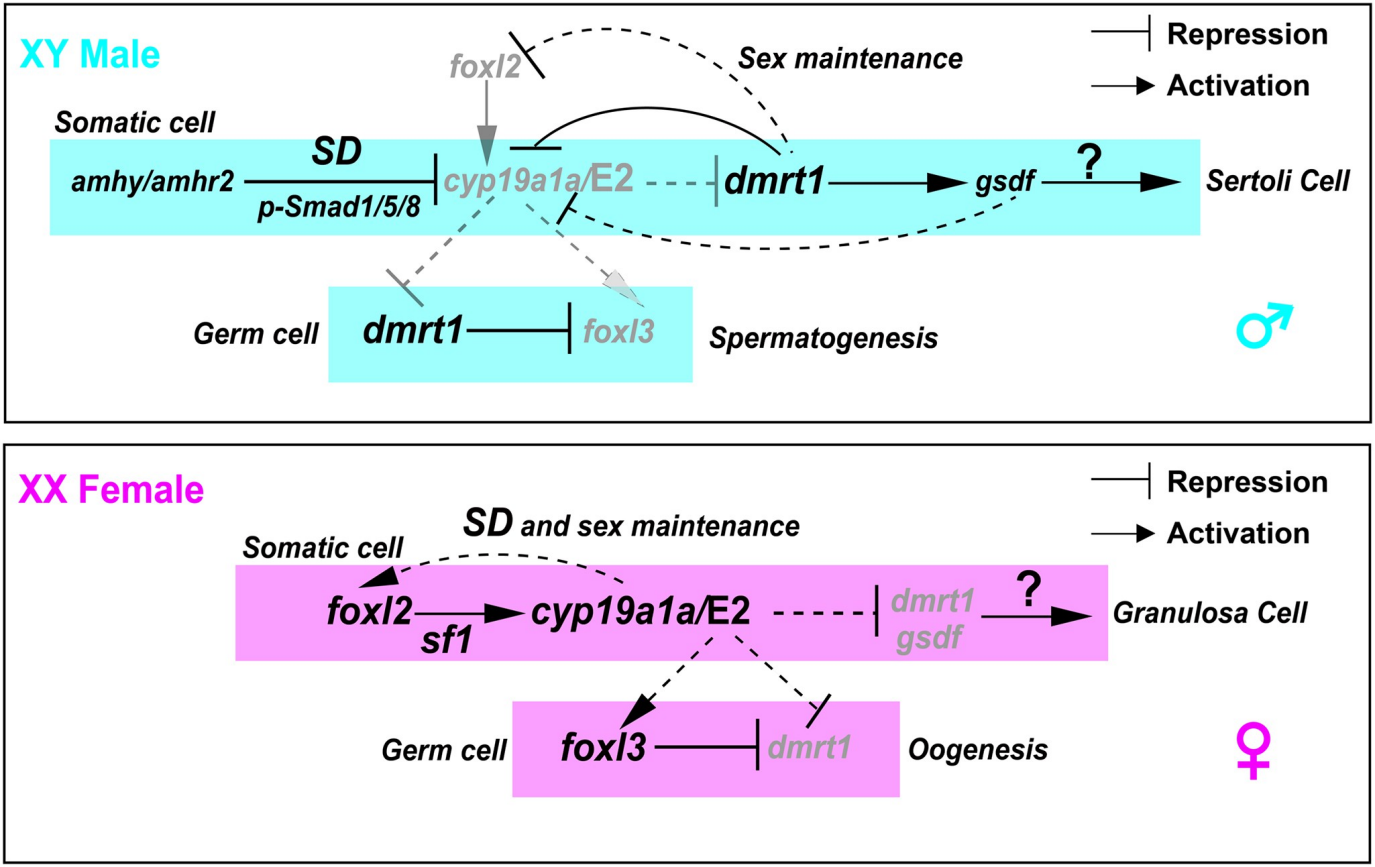
A proposed model for tilapia female and male sex determination and maintenance. In XY tilapia, repression of Cyp19a1a expression/E2 synthesis by the MSD gene *amhy* in somatic cells is the prerequisite for up-regulation of Dmrt1 expression in both somatic and germ cells to initiate testis development. Dmrt1 and its downstream target Gsdf repress *cyp19a1a* and *foxl2*/*foxl3* expression to maintain male sex. In XX tilapia, induction of Cyp19a1a expression/E2 synthesis by *foxl2* is necessary to initiate ovarian differentiation by repressing *dmrt1* expression and activating of *foxl3* expression. Continuous expression of Cyp19a1a for estrogen production is required to maintain ovarian fate by sustaining high expression of *foxl2* in female, suggesting a positive feedback loop between estrogen and *foxl2*. The signals from somatic cells (*amhy* and estrogen) determine the fate of germ cells by affecting Dmrt1 and Foxl3 expression.

A large number of studies have shown in mouse, chicken and fish that Dmrt1*/dmrt1* is expressed in both germ cells and somatic cells [29,66–68]. In tilapia, female pathway genes *foxl2* and *cyp19a1a* are expressed exclusively in somatic cells [13,69], while *foxl3* is expressed exclusively in germ cells [14]. Of the tilapia male pathway genes, *amhy* and *gsdf* are expressed exclusively in somatic cells [9, 12], while *dmrt1* is expressed in both germ cells and somatic cells [14,70]. Recently, we have demonstrated that *amhy* inhibits the transcription of *cyp19a1a* through Amhr2/Smads signaling to disrupt E2 synthesis, which in turn, induces *dmrt1* expression in somatic cells in XY tilapia during the critical period of SD [43]. In XY tilapia, up-regulation of Dmrt1 in germ cells directly inhibits *foxl3* transcription [14], and up-regulation of Dmrt1 in somatic cells directly represses *cyp19a1a* transcription and directly or indirectly inhibits *foxl2* expression to initiate and maintain the testicular fate [53]. In XX tilapia, repression of Dmrt1 expression in somatic and germline by estrogen, which is promoted by *foxl2* and *sf-1* [21], is the crucial step for initiating and maintaining ovarian fate. In this study, by generation of 9 double and 4 triple mutants, we provided strong evidences to support this proposed SD mechanism in tilapia. Based on these results, a model for the regulatory architecture in tilapia SD and sex maintenance is proposed in Fig 7. Therefore, the indispensable role of Dmrt1 in testicular differentiation and development in male might be attributed to its expression in both somatic and germ cell. Additionally, *doublesex* (*dsx*), encodes a transcription factor with DM domain, that determines sex in invertebrates, including the fruit fly (*Drosophila*), nematode (*Caenorhabditis elegans*) and silkworm (*Bombyx mori*) [71–74]. Therefore, *dmrt1* is commonly used as a key male pathway gene in vertebrates and even in some invertebrates.

## Conclusions

In this study, we analyzed 12 double and 4 triple mutants, including 3 double mutants established previously, in Nile tilapia. Based on the gonadal phenotypes and gene expressions of these mutants, we found that once *dmrt1* is mutated, the gonad cannot be rescued to a functional testis by mutation of any female pathway gene. The SR caused by mutation of male pathway genes other than *dmrt1* can be rescued by mutation of the female pathway gene or AI treatment (S12 Fig). Taken together, our results demonstrated for the first time that *dmrt1* is the only male pathway gene tested so far indispensable for SD and functional testis development. Our work may shed some light on the genetic interaction between female and male pathway genes in tilapia, which enhances our understanding of SD in vertebrates.

## Materials and methods

### Ethics

All animal experiments were conducted in accordance with the regulations of the Guide for Care and Use of Laboratory Animals and were approved by the Committee of Laboratory Animal Experimentation at Southwest University.

### Nile tilapia

The Nile tilapia, *Oreochromis niloticus*, were reared in recirculating freshwater aquarium at 26°C with a photoperiod of 14 hours of light and 10 hours of dark. The experimental fish were fed three time a day with commercial dry food (Shengsuo, China). In our cultured conditions, all-XX and all-XY tilapia develop as females and males, respectively.

### Generation of double and triple mutant lines

The XX and XY heterozygotes for *amhy*, *dmrt1*, *gsdf*, *cyp19a1a*, *cyp19a1b* and *foxl3* alleles were previously generated by CRISPR/Cas9 [9, 12–14, 42, 43]. These mutant lines were maintained in our laboratory. For generation of *dmrt1*^-/-^;*cyp19a1a*^-/-^ double mutants, firstly, the *dmrt1*^+/-^;*cyp19a1a*^+/-^ double heterozygotes were obtained by crossing *dmrt1*^+/-^ XY males with *cyp19a1a*^+/-^ XX females. Subsequently, the *dmrt1*^-/-^;*cyp19a1a*^-/-^ double mutants were obtained by crossing *dmrt1*^+/-^;*cyp19a1a*^+/-^ XY males with *dmrt1*^+/-^;*cyp19a1a*^+/-^ XX females. The *gsdf*^-/-^;*cyp19a1a*^-/-^, *gsdf*^-/-^;*foxl3*^-/-^ and *cyp19a1a*^-/-^;*cyp19a1b*^-/-^ double mutants were generated by the same strategy. In addition, the *amhy*^−^;*cyp19a1a*^+/-^ XY fish was obtained by crossing *amhy*^−^ XY neo-females (this sex reversed female is fertile that can produce functional eggs [43]) with *cyp19a1a*^-/-^ XX neo-males. Then, the *amhy*^−^;*cyp19a1a*^-/-^ double mutants were obtained by crossing *amhy*^−^;*cyp19a1a*^+/-^ XY neo-females with *cyp19a1a*^-/-^ XX neo-males. The *amhy*^−^;*foxl3*^+/-^ XY fish was obtained by crossing *amhy*^−^ XY neo-females with *foxl3*^-/-^ XY males. Then, the *amhy*^−^;*foxl3*^-/-^ XY fish was obtained by crossing *amhy*^−^;*foxl3*^+/-^ XY neo-females with *foxl3*^-/-^ XY males. For generation of *dmrt1*^-/-^;*cyp19a1a*^-/-^;*cyp19a1b*^-/-^ triple mutants, *dmrt1*^+/-^;*cyp19a1a*^+/-^;*cyp19a1b*^+/-^ triple heterozygotes were obtained by mating *dmrt1*^+/-^;*cyp19a1a*^+/-^ XY males with *cyp19a1b*^+/-^ XX females. Then, the *dmrt1*^-/-^;*cyp19a1a*^-/-^;*cyp19a1b*^-/-^ triple mutants were obtained by mating *dmrt1*^+/-^;*cyp19a1a*^+/-^;*cyp19a1b*^+/-^ XY males with *dmrt1*^+/-^;*cyp19a1a*^+/-^;*cyp19a1b*^+/-^ XX females. For generation of *amhy*^−^;*cyp19a1a*^-/-^;*cyp19a1b*^-/-^ triple mutants, *amhy*^−^;*cyp19a1a*^+/-^;*cyp19a1b*^+/-^ heterozygotes were obtained by mating *amhy*^−^;*cyp19a1a*^+/-^ XY neo-females with *cyp19a1b*^+/-^ XY males. The *amhy*^−^;*cyp19a1a*^-/-^;*cyp19a1b*^-/-^ triple mutants were further obtained by mating *amhy*^−^;*cyp19a1a*^+/-^;*cyp19a1b*^+/-^ XY neo-females with *cyp19a1a*^+/-^;*cyp19a1b*^+/-^ XY males. Both the *gsdf* and *cyp19a1b* genes were located in the chromosome 7. The *gsdf*^-/-^;*cyp19a1a*^-/-^;*cyp19a1b*^-/-^ triple mutants cannot be obtained by crossing using *gsdf*^*+*/-^;*cyp19a1a*^*+*/-^ double heterozygotes and *cyp19a1b*^*+*/-^ heterozygotes. In our previous study, the *cyp19a1b* was efficiently targeted by CRISPR/Cas9 [42]. For generation of *gsdf*^-/-^;*cyp19a1a*^-/-^;*cyp19a1b*^-/-^ triple mutants, the gRNA for *cyp19a1b* was *in vitro* transcription with T7 RNA polymerase followed by purification with QIAquick PCR Purification Kit (QIAGEN, Germany). The construct containing humanized Cas9 cDNA was kindly provided by Dr Jingwei Xiong, Peking University [75]. Cas9 mRNA was synthesized by T7 mMESSAGE mMACHINE Kit (Ambion, USA) according to the manufacturer’s instructions. The mixture of gRNA and Cas9 mRNA, at a final concentration of 250 ng/μl and 500 ng/μl, respectively, was co-injected into one-cell stage embryos from crossing *gsdf*^-/-^;*cyp19a1a*^-/-^ XY males with *gsdf*^+/-^;*cyp19a1a*^+/-^ XX females. Injected embryos were raised to adulthood, and the F0 mutants were screened by restriction enzyme digestion and sequencing. The *gsdf*^+/-^;*cyp19a1a*^+/-^;*cyp19a1b*^+/-^ triple heterozygotes were obtained by mating *gsdf*^+/-^;*cyp19a1a*^+/-^;*cyp19a1b* F0 XY males with WT XX females. The *gsdf*^-/-^;*cyp19a1a*^-/-^;*cyp19a1b*^-/-^ triple mutants were obtained by crossing *gsdf*^+/-^;*cyp19a1a*^+/-^;*cyp19a1b*^+/-^ XY males with *gsdf*^+/-^;*cyp19a1a*^+/-^;*cyp19a1b*^+/-^ XX females. For generation of *dmrt1*^-/-^;*cyp19a1a*^-/-^;*foxl3*^-/-^ triple mutants, *dmrt1*^+/-^;*cyp19a1a*^+/-^;*foxl3*^+/-^ triple heterozygotes were obtained by mating *dmrt1*^+/-^;*cyp19a1a*^+/-^ XY males with *foxl3*^+/-^ XX females. The *dmrt1*^-/-^;*cyp19a1a*^-/-^;*foxl3*^-/-^ triple mutants were obtained by mating *dmrt1*^+/-^;*cyp19a1a*^+/-^;*foxl3*^+/-^ XY males with *dmrt1*^+/-^;*cyp19a1a*^+/-^;*foxl3*^+/-^ XX females. The establishment of these mutants were shown in S13 Fig.

Previously, our studies showed that mutation of *foxl2* in F0 XX tilapia by TALEN induced female-to-male SR [14,56]. In this study, *in vitro* synthesis of mRNA for *foxl2* TALENs was carried out with Sp6 mMESSAGE mMACHINE Kit (Ambion, USA). The synthesized mRNA was purified using a MEGAclear Transcription Clean-Up Kit (Ambion, USA) according to the manufacturer’s instructions. About 500–800 pg mRNA was microinjected into embryos obtained from crossing *gsdf*^+/-^ XX females with *gsdf*^-/-^ XY males or embryos obtained from crossing *amhy*^−^ XY neo-females with XX neo-males using the WPI Micro-injector. The *gsdf*^-/-^ XY males and XX neo-males were obtained by administration of letrozole (200 μg/g diet) from 5 to 30 dah in our laboratory. The injected embryos were hatched at 26°C and collected to extract genomic DNA (gDNA) for mutation analysis at 45 and 60 dah as described previously [14].

### DNA extraction, genotyping and genetic sex identification

gDNA was extracted from a piece of caudal fin by proteinase K digestion followed by phenol/chloroform extraction. The quality and concentration of gDNA were assessed by agarose gel electrophoresis, and measured using a NanoDrop 2000 spectrophotometer (Thermo Fisher Scientific, USA). Then, the gDNA was diluted to a concentration of 100 ng/μl for use. DNA fragments spanning the target site of each gene were amplified using the primers listed in S1 Table. The amplified DNA fragments were recovered using a QIAquick PCR Purification Kit (QIAGEN, Germany) according to the manufacturer’s instructions. Restriction enzyme digestion was carried out for distinguishing different genotypes of the single, double and triple mutants established in this study, including wild-types, heterozygotes and homozygotes as described previously [9,12–14,42,43]. To determine whether *foxl2* mutations were generated in the *amhy* and *gsdf* mutants, restriction enzyme digestion was performed to evaluate mutation efficiency in each fish as described in our previous studies [14,56].

Genotypic sex of all experimental fish was determined by genomic PCR using tilapia sex specific marker-5 developed previously [76]. Genotyping PCR was performed using GoTaq PCR Master Mix (Promega, USA). Cycling parameters were as follows: 95°C for 3 min, 95°C for 30 s, 58°C for 30 s, 72°C for 40 s, 72°C for 10 min (steps 2 to 4 run for 36 cycles). Primer sequences were listed in the S1 Table.

### Drug treatment

In the treatment group, the newly hatched fry were fed with commercial diet sprayed with 95% ethanol containing letrozole (the third-generation aromatase inhibitor) (Sigma-Aldrich, USA) at a concentration of 200 μg/g feed from 5 to 30 or 60, 120 dah. The control group fish were fed with normal commercial diet sprayed with 95% ethanol only. Later on, all fish were fed with normal commercial diet. The treatment experiments were repeated three times. The gonad phenotype and the gene expressions were determined at different time points.

### Gonadal histological examination

The control and mutant fish were sampled at different time points for phenotype analysis. Firstly, the experimental fish were anesthetized by immersion in 0.16 mg/ml tricaine methanesulfonate (MS-222) (Sigma-Aldrich, USA). Secondly, the gonad samples were fixed in Bouin’s solution for 24 h at room temperature. They were then dehydrated and embedded in paraffin. Tissue blocks were sectioned at 5 μm thickness using the Leica microtome and stained with Hematoxylin and Eosin (H&E) as described previously [53]. The images were taken using the Olympus BX53F microscope.

### RNA extraction, RT-PCR and real-time PCR

Different tissues, including liver, ovary, testis and brains, were collected from different genotype individuals (usually three to five parallel samples per genotype) for gene expression analysis. Samples were immediately frozen in liquid nitrogen. Total RNAs were extracted from all samples using a column-based RNA extraction kit (Qiagen, USA) specialized for small quantities of RNA. The concentration of total RNA was quantified by using NanoDrop 2000. DNase I (RNase free) treatment and cDNA preparation were carried out using PrimeScript RT reagent Kit with gDNA Eraser (Takara, Japan) according to the manufacturer’s instructions. RT-PCR was performed to check the gene expression according to the methods previously described [69]. Real-time PCR was performed with Fast SYBR Green Master Mix (Takara, Japan) on a 7500 Fast Real-Time PCR system (Applied Biosystems, USA). Housekeeping gene *β-actin* was used as the internal control, which was well working in our previous study [13]. The expression of target genes in each genotype was normalized to that of the *β-actin*. The relative abundance of mRNA transcripts was calculated using the formula: R = 2^-ΔΔCt^ as described previously [77]. At least three samples for each genotype were analyzed. Primer sequences used for RT-PCR and real-time PCR were listed in S1 Table.

### Immunofluorescence (IF) and whole-mount IF

For immunofluorescence, the sections were permeabilized with 1% Triton X-100 in PBS for 10 min and then blocked in 5% bovine serum albumin (BSA)/PBS for 30 min at room temperature. The sections were then incubated with primary antibodies in 5% BSA/PBS overnight at 4°C. For whole-mount immunofluorescence (IF), gonads were collected and fixed in 4% PFA overnight at 4°C. The gonads were permeabilized with 100% acetone for 20 min before primary antibody incubation. The following rabbit polyclonal antibodies were prepared by our laboratory: Amh (This antibody can recognize both Amh and Amhy proteins), Gsdf, 3β-HSD-I, Vasa, 42Sp50, Zar1, Cyp19a1a, Cyp19a1b, Creb1b, Sox30 and Cyp11c1 (S2 Table). The dilution and specificity of these antibodies have been analyzed previously [9,12–14,42,43,78,79]. Goat anti-rabbit antibody Alexa Fluor 488- and 594-conjugated secondary antibodies (Thermo Fisher Scientific, USA) were diluted to 1:500 in blocking solution and incubated with samples overnight at 4°C to detect the primary antibodies. The nuclei were stained by 4’, 6-diamidino-2-phenylindole (DAPI) using VECTASHIELD Mounting Medium with DAPI (Vector, USA). At least three independent biological replicates for each genotype were analyzed. Fluorescence signals were captured by confocal microscope (Olympus FV3000, Japan).

### Fluorescence *in situ* hybridization (FISH) and whole-mount FISH

For FISH, the gonads of WT and mutant fish were collected at indicated time. The samples were fixed in 4% paraformaldehyde (Sigma-Aldrich, USA) in 0.85× PBS at 4°C overnight. Specimens were embedded in paraffin and sectioned at 5 μm. For whole-mount FISH, larvae were washed with PBS plus Triton X-100 for three times to increase tissue permeability and improve staining while preserving the antigen epitopes. FISH and whole-mount FISH were performed to examine the gene expression as described previously [80]. Probes for *foxl3* and *dmrt1* antisense or sense digoxigenin-labeled RNA strands were transcribed *in vitro* from a linearized pGEM-T easy-*foxl3* and *dmrt1* plasmid using the T7 RNA labeling kit (Roche, Swiss). The sections were deparaffinized, rehydrated, and digested with proteinase K at 37°C for 20 min. After refixation by 4% paraformaldehyde, the sections were hybridized with DIG-labeled RNA probe at 65°C overnight. For more sensitive FISH and whole-mount FISH detection, the tyramide signal amplification (TSA) Plus Fluorescence Systems (PerkinElmer Life Science, USA) were used according to the manufacturer′s instructions. After mounting with VECTASHIELD Mounting Medium with DAPI (Vector, USA), the images were recorded using confocal microscope (Olympus FV3000, Japan). Primers for preparing DIG-labeled probes are list in S1 Table. The fluorescence intensity of Cyp19a1b staining was quantified using Image J software as described previously [81]. At least 4 sections from different fish are randomly selected for fluorescence signal intensity quantification.

### Transcriptome sequencing and analysis

Total RNAs extracted from gonads of WT XX, WT XY, *dmrt1*^-/-^ XY, *dmrt1*^-/-^;*cyp19a1a*^-/-^;*cyp19a1b*^-/-^ triple mutants, AI treated-*dmrt1*^-/-^;*foxl3*^-/-^ and *dmrt1*^-/-^;*cyp19a1a*^-/-^;*foxl3*^-/-^ fish at 60 dah were 2×100-bp paired-end sequenced using the HiSeq 2000 platform (Illumina). Clean reads from each library were aligned to the reference genome (O_niloticus_UMD_NMBU (GCA_001858045.3, http://asia.ensembl.org/Oreochromis_niloticus/Info/Index) using Tophat with the default parameters. The Fragments per kilobase of exon per million fragments mapped (FPKM) method was used to calculate gene expression levels. The assembled transcripts were merged using the reference annotation (*Oreochromis niloticus*: Orenil1.0.78.gtf, downloaded from Ensembl) with Cuffmerge, while differential expression analysis was performed with Cuffdiff. The FPKM of somatic and germ cell related genes were normalized and clustered using TBtools software [82].

### Measurement of serum hormone level by EIA and UPLC-MS/MS

Blood samples were collected from the caudal veins of experimental fish, and kept at 4°C overnight. Serum was collected after centrifugation and stored at −80°C until use. Serum estradiol-17β (E2) level was measured using Estradiol ELISA Kit (Cayman, USA) according to the manufacturer′s instructions. Absorbance was measured at a wavelength of 412 nm using a Multiskan GO microplate reader (Thermo Fisher Scientific, USA). To further confirm the serum estrogen level, we adopted Ultra Performance Liquid Chromatography Tandem Mass Spectrometry (UPLC-MS/MS) to measure it as reported previously [83].

### Detection of apoptosis by TUNEL

The gonads for Terminal Deoxynucleotidyl Transferase mediated dUTP Nick-End Labeling (TUNEL) experiment were fixed with 4% paraformaldehyde (PFA) for 12 hours at room temperature. They were then dehydrated and embedded in paraffin. Tissue blocks were sectioned at 5 μm and permeabilized by proteinase K for 15 min, and subsequently fixed again with 4% PFA for 30 min at room temperature. For TUNEL assay, the sections were incubated in 1:10 dilution of 50 μl reaction mixture using *In Situ* Cell Death Detection Kit (Roche, Swiss) at 37°C for 60 min in the dark followed by washing with 1× PBS. The sections were then incubated in blocking solution for 2 hours at room temperature and stained with anti-Cyp19a1b antibody for 2 hours at 37°C. Following washes in 1× PBS, the sections were incubated with 1:500 donkey anti-rabbit Alexa Fluor 488 (Thermo Fisher Scientific, USA) for 2 hours at room temperature, followed by washes in 1× PBS. After mounting with VECTASHIELD Mounting Medium with DAPI (Vector, USA), the images were recorded using confocal microscope (Olympus FV3000, Japan).

### Fertility assay

Fertility of the adult mutant fish (*amhy*^-^;*cyp19a1a*^-/-^;*cyp19a1b*^-/-^ and *gsdf*^-/-^;*cyp19a1a*^-/-^;*cyp19a1b*^-/-^) was assessed via artificial insemination. For each mating, the number of fertilized eggs of wild-type XX females was counted.

### Sperm morphology and motility analyses

For WT XY and XX *foxl3*^-/-^ fish at 120 dah, we dissected and obtained gonads, and crushed them to obtain gonadal suspension, which were collected in a clean and sterile 1.5 mL centrifuge tube. For sperm morphology analysis, sperm of WT XY and XX *foxl3*^-/-^ fish at 120 dah were stained with the fluorescent membrane dye PKH26 (Sigma-Aldrich, USA), which incorporates aliphatic reporter molecules into the cell membrane. Approximately ten million cells were suspended in 0.4 ml of diluent C (anisoosmotic aqueous solution provided with the PKH26 dye) in which PKH26 was diluted at a ratio of 4 μl dye to 0.4 ml diluent C. The diluted dye was incubated with the cells (final concentration, 10 μmol/l) for 5 min. The cells were then centrifuged at 100×g for 5 min, washed twice in L-15 (HyClone, USA), resuspended in L-15, and then observed with confocal microscope (Olympus FV3000, Japan) [84]. For sperm motility analysis, sperm was collected from WT XY and XX *foxl3*^-/-^ fish at 120 dah. After a dilution at 1:10 with phosphatebuffered saline, 10 mL diluted semen was added to the counting chamber of the sperm count plate and captured on the stage of a Leica DM500 light microscope (Leica, Germany) [55].

### Statistical analysis

All the experiments were conducted for at least three times. Data were analyzed using GraphPad Prism 8 software and expressed as the mean ± SD. Significance of differences among more than two groups were calculated using One-way ANOVA, followed by Tukey test for multiple comparisons. For all statistical analyses, *P*<0.05 indicated a significant difference labeled by different letters in the figures, and groups labeled with the same letter were not significantly different from each other.

## Supporting information

S1 Fig Timeline of tilapia XY male and XX female gonad development. Previous studies demonstrated that a number of genes showed sexually dimorphic expression between XX and XY gonads at 5 dah including *amhy*, *dmrt1*, *gsdf*, *foxl2* and *cyp19a1a*, indicating the critical time for sex determination [50,51]. The first characteristic of gonadal differentiation occurs in tilapia larvae between 20 and 25 dah with appearance of the ovarian cavity in the XX gonad. The efferent duct is observed in the XY testis at around 40 dah. The germ cell meiosis in XX gonads initiates at 25–30 dah and oocyte can be observed thereafter, but germ cells does not initiate meiosis in XY gonads until 60 dah and spermatocyte can be observed [52].
(TIF)

S2 Fig Gonadal phenotypes and gene expressions in *dmrt1*, *cyp19a1a*, *cyp19a1b* single and *cyp19a1a*;*cyp19a1b* double mutants at early stages. **(A)** Expression of Cyp19a1a in the gonads of the WT XX, WT XY, XY *dmrt1*^-/-^ and XX *dmrt1*^-/-^ fish at 5 dah by Whole-mount IF. Scale bars = 40 μm. **(B)** Histological examination of gonads from XX *cyp19a1a*^-/-^, XX *cyp19a1b*^-/-^, XX *cyp19a1a*^-/-^;*cyp19a1b*^-/-^ and AI treated-XX *cyp19a1a*^-/-^ tilapia at 45 dah using hematoxylin and eosin (H&E) staining. Expressions of Leydig cell marker Cyp11c1 and oocyte marker 42Sp50 were analyzed by immunofluorescence (IF). AI, aromatase inhibitor, Letrozole. Scale bars = 40 μm. **(C)** Sex ratios in XX *cyp19a1a*^-/-^, XX *cyp19a1b*^-/-^, XX *cyp19a1a*^-/-^;*cyp19a1b*^-/-^ and AI treated-XX *cyp19a1a*^-/-^ tilapia at 45 dah. **(D)** Expression of *dmrt1* mRNA in the gonads of the WT XX, WT XY and XX *cyp19a1a*^-/-^;*cyp19a1b*^-/-^ fish at 5 dah by Whole-mount FISH. Nuclei were counterstained with DAPI. Scale bars = 40 μm. Oc, oocyte. Ocv, ovarian cavity. Sg, spermatogonia. Ed, efferent duct. dah, days after hatching.
(TIF)

S3 Fig Gonadal phenotype and gene expressions in *dmrt1*^-/-^;*cyp19a1a*^-/-^ double mutants at 120 dah. **(A)** Histological examination of gonads from WT XX, WT XY, XY *dmrt1*^-/-^ and XX/XY *dmrt1*^-/-^;*cyp19a1a*^-/-^ tilapia at 45 dah by H&E staining. Scale bars = 40 μm. **(B)** Histological examination of gonads from WT XX, WT XY, XY *dmrt1*^-/-^, XX *cyp19a1a*^-/-^, XX/XY *dmrt1*^-/-^;*cyp19a1a*^-/-^ tilapia at 120 dah using H&E staining. Oc, oocyte. Ocv, ovarian cavity. Sg, spermatogonia. Sc, spermatocyte. Ed, efferent duct. Scale bars = 40 μm. **(C)** Sex ratios in XY *dmrt1*^-/-^, XX *cyp19a1a*^-/-^ and XX/XY *dmrt1*^-/-^;*cyp19a1a*^-/-^ tilapia at 120 dah. **(D)** Gene expressions in the underdeveloped testis of *dmrt1*^-/-^;*cyp19a1a*^-/-^ mutants (type II) detected by IF at 120 dah. Vasa for germ cell. Amh for Sertoli cell. Cyp11c1 and 3β-HSD-I for Leydig cell. Nuclei were counterstained with DAPI. WT, wild-type. dah, days after hatching. Scale bars = 40 μm.
(TIF)

S4 Fig The follicles of *dmrt1*^-/-^;*cyp19a1a*^-/-^ double mutants developed to vitellogenic stage. **(A)** Anatomical examination of the gonads from WT XX, WT XY and XX/XY *dmrt1*^-/-^;*cyp19a1a*^-/-^ type I tilapia at 150 dah. The asterisk indicates ovum. Histological examination of gonads from WT XX, WT XY, XX/XY *dmrt1*^-/-^;*cyp19a1a*^-/-^ type I at 150 dah by H&E staining. Arrow and arrowhead indicates granulosa cell (G) and theca cell (T), respectively. Vtg, vitellogenin. Sg, spermatogonia. Sc, spermatocyte. Sz, spermatozoa. Scale bars = 40 μm. **(B)** RT-PCR analysis of three *vitellogenin* (*vtg1*, *vtg2* and *vtg3*) expressions in the livers of WT XX, WT XY, XY *dmrt1*^-/-^, XX *cyp19a1a*^-/-^ and XX/XY *dmrt1*^-/-^;*cyp19a1a*^-/-^ type I at 150 dah. *β-actin* was used as an internal control. DKO, XX/XY *dmrt1*^-/-^;*cyp19a1a*^-/-^ double mutants. **(C)** Serum E2 level in WT XX, WT XY and XX/XY *dmrt1*^-/-^;*cyp19a1a*^-/-^ type I was measured by ELISA and UPLC-MS methods. Data were expressed as the mean ± SD of triplicates. Different letters above the error bars indicate statistical differences at *P*<0.05 as determined by one-way ANOVA followed by Tukey test. **(D)** Cellular locations of Cyp19a1a and Cyp19a1b proteins in the ovaries of WT XX, XX *cyp19a1b*^-/-^, XY *dmrt1*^-/-^ and XX/XY *dmrt1*^-/-^;*cyp19a1a*^-/-^ type I double mutants analyzed by IF. Nuclei were counterstained with DAPI. Arrow and arrowhead indicates granulosa cell (G) and theca cell (T), respectively. Scale bars = 40 μm. WT, wild-type. dah, days after hatching.
(TIF)

S5 Fig Gonadal development and gene expressions in the *dmrt1*^-/-^;*cyp19a1a*^-/-^ double mutants and *dmrt1*^-/-^;*cyp19a1a*^-/-^;*cyp19a1b*^-/-^ triple mutants. **(A)** Real-time PCR analysis of genes related to gonadal steroidogenesis, oogonia and oocyte in WT XX, XY *dmrt1*^-/-^ and XX/XY *dmrt1*^-/-^;*cyp19a1a*^-/-^;*cyp19a1b*^-/-^ mutants at 60 dah. *β-actin* was used as an internal control. Data were expressed as the mean ± SD of triplicates. Different letters above the error bars indicate statistical differences at *P*<0.05 as determined by one-way ANOVA followed by Tukey test. **(B)** The germ cells of WT XX, WT XY, XX/XY *dmrt1*^-/-^, XX *cyp19a1a*^-/-^;*cyp19a1b*^-/-^ and XX/XY *dmrt1*^-/-^;*cyp19a1a*^-/-^;*cyp19a1b*^-/-^ tilapia were stained using Vasa antibody at 15 and 25 dah. The germ cell number in each genotype was counted (n = 3). Scale bars = 200 μm. **(C)** Histological examination of gonads from *dmrt1*^-/-^;*cyp19a1a*^-/-^ double mutants (type I) and *dmrt1*^-/-^;*cyp19a1a*^-/-^;*cyp19a1b*^-/-^ triple mutants at 150 dah by H&E staining. Arrow and arrowhead indicates granulosa cell (G) and theca cell (T), respectively. Oc, oocyte. Scale bars = 40 μm. **(D)** RT-PCR analysis of *vtg1*, *vtg2* and *vtg3* mRNA expressions in the livers of type I *dmrt1*^-/-^;*cyp19a1a*^-/-^ double mutants (DKO type I) and *dmrt1*^-/-^;*cyp19a1a*^-/-^;*cyp19a1b*^-/-^ triple mutants (TKO) at 150 dah. *β-actin* was used as an internal control. **(E)** Anatomical examination of ovaries from XX/XY DKO type I, XX/XY TKO and WT XX tilapia at 240 dah. Histological examination of the gonads from DKO type I, TKO and WT XX tilapia by H&E staining at 240 and 360 dah. Expression of Cyp19a1b in the gonads of WT XX, DKO type I and TKO tilapia was analyzed by IF. TUNEL analysis showed the apoptosis of granulosa cells and theca cells in the ovaries of DKO type I and TKO mutants, but not in the WT XX at 240 dah. DKO, XX/XY *dmrt1*^-/-^;*cyp19a1a*^-/-^ double mutants; TKO, XX/XY *dmrt1*^-/-^;*cyp19a1a*^-/-^;*cyp19a1b*^-/-^ triple mutants. Nuclei were counterstained with DAPI. Scale bars = 40 μm. WT, wild-type. dah, days after hatching.
(TIF)

S6 Fig Double mutation of *foxl3* and *cyp19a1a* resulted in testis development in XX tilapia. Expression of somatic cell and germ cell markers in the gonads of WT XX, WT XY, XX *cyp19a1a*^-/-^, XX *foxl3*^-/-^ and XX *cyp19a1a*^-/-^;*foxl3*^-/-^ tilapia were analyzed by IF at 120 dah. Cyp19a1a, a female somatic specific marker. 42Sp50, an oocyte marker. Cyp11c1, a Leydig cell marker. Gsdf, a somatic cell marker expressed highly in males compared with females. Creb1b, a spermatocyte marker. Nuclei were counterstained with DAPI. WT, wild-type. dah, days after hatching. Scale bars = 40 μm.
(TIF)

S7 Fig The gonads of *dmrt1*^-/-^;*foxl3*^-/-^ double mutants developed as ovaries after withdrawal of AI treatment. **(A)** The WT XX, *dmrt1*^-/-^ single, *dmrt1*^-/-^;*foxl3*^-/-^ double mutants were treated by AI from 5 to 60 dah. The gonadal samples were collected at 60 and 90 dah for histological examination. At 60 dah, the gonads of AI treated-WT XX tilapia displayed testicular development, while the gonads of AI treated-XX/XY *dmrt1*^-/-^ tilapia still developed as ovaries with oocyte development. The gonads of AI treated-*dmrt1*^-/-^;*foxl3*^-/-^ double mutants exhibited testicular morphology with spermatocyte-like cells at 60 dah. However, at 90 dah, the gonads of AI treated-*dmrt1*^-/-^;*foxl3*^-/-^ double mutants developed as ovotestis with both previtellogenic follicles and spermatocyte-like cells. Scale bars = 50 μm. **(B)** The *dmrt1*^-/-^;*foxl3*^-/-^ double mutants were treated by AI from 5 to 120 dah. The gonadal samples were collected at 120, 150 and 240 dah for histological examination. At 120 dah, the gonads of AI treated-*dmrt1*^-/-^;*foxl3*^-/-^ double mutants displayed testis morphology with spermatocyte-like cells. However, the gonads of AI treated-*dmrt1*^-/-^;*foxl3*^-/-^ double mutants developed as ovotestis with many previtellogenic follicles at 150 dah. Subsequently, the gonads of AI treated-*dmrt1*^-/-^;*foxl3*^-/-^ double mutants developed as ovaries with a large number of vitellogenic follicles at 240 dah. Scale bars = 50 μm. AI, aromatase inhibitor, Letrozole. Vtg, vitellogenin. Oc, oocyte. Ocv, ovarian cavity. Sg, spermatogonia. Sc, spermatocyte. Sz, spermatozoa. Ed, efferent duct. WT, wild-type. dah, days after hatching.
(TIF)

S8 Fig Somatic cells but not germ cells were feminized in *amhy*^-^;*foxl3*^-/-^ and *gsdf*^-/-^;*foxl3*^-/-^ double mutants at 120 dah. **(A)** Histological examination of gonads from WT XX, WT XY, XY *amhy*^−^, XX/XY *gsdf*^-/-^, XX *foxl3*^-/-^, XY *amhy*^−^;*foxl3*^-/-^ and XX/XY *gsdf*^-/-^;*foxl3*^-/-^ tilapia at 120 dah using H&E staining. Oc, oocyte. Sg, spermatogonia. Sc, spermatocyte. Expression of somatic cell and germ cell markers in the gonads of these mutants were analyzed by IF. Cyp19a1a, a female somatic specific marker. 42Sp50, an oocyte marker. Cyp11c1, a Leydig cell marker. Gsdf, a somatic cell marker. Creb1b, a spermatocyte marker. Sox30, a male germ cell marker expressed highly in spermatozoa. Nuclei were counterstained with DAPI. Scale bars = 40 μm. **(B)** The germ cells in WT XX, WT XY, XY *amhy*^−^, XX/XY *gsdf*^-/-^, XX *foxl3*^-/-^, XY *amhy*^−^;*foxl3*^-/-^ and XX/XY *gsdf*^-/-^;*foxl3*^-/-^ mutants were shown by whole-mount IF with Vasa antibody at 15 dah. The germ cell number in each genotype was counted (n = 4). WT, wild-type. dah, days after hatching. Scale bars = 200 μm.
(TIF)

S9 Fig AI treatment and mutation of *cyp19a1b* resulted testis development in the *amhy*^−^;*cyp19a1a*^-/-^ and *gsdf*^-/-^;*cyp19a1a*^-/-^ double mutants. **(A)** Histological examination of gonads from XX *cyp19a1a*^-/-^, XY *amhy*^−^, XY *amhy*^−^;*cyp19a1a*^-/-^, XY *gsdf*^-/-^ and XX/XY *gsdf*^-/-^;*cyp19a1a*^-/-^ mutants at 45 dah by H&E staining. Scale bars = 40 μm. **(B)** AI treatment resulted in testis development in *amhy*^−^;*cyp19a1a*^-/-^ and *gsdf*^-/-^;*cyp19a1a*^-/-^ double mutants as revealed by 42Sp50 and Cyp11c1 staining at 45 dah. These two double mutants were treated with AI from 5 to 30 dah. AI, aromatase inhibitor, letrozole. Scale bars = 40 μm. **(C)** Expression of Cyp19a1b protein in the brains and ovaries of *amhy*^−^, *gsdf*^-/-^ single mutants, *amhy*^−^;*cyp19a1a*^-/-^ and *gsdf*^-/-^;*cyp19a1a*^-/-^ double mutants at 45 dah detected by IF. Nuclei were counterstained with DAPI. Scale bars = 20 μm. **(D)** Real-time PCR analysis of *cyp19a1b* mRNA expression level in the brains and gonads of XY *amhy*^−^, XY *gsdf*^-/-^ single mutants and XY *amhy*^−^;*cyp19a1a*^-/-^ and XX/XY *gsdf*^-/-^;*cyp19a1a*^-/-^ double mutants. Expression was normalized to *β-actin*. Data were expressed as the mean ± SD. Different letters above the error bars indicate statistical differences at *P*<0.05 as determined by one-way ANOVA followed by Tukey test. **(E)** Histological examination of gonads from WT XY, XY *amhy*^−^;*cyp19a1a*^-/-^;*cyp19a1b*^-/-^ and XX/XY *gsdf*^-/-^;*cyp19a1a*^-/-^;*cyp19a1b*^-/-^ triple mutants at 120 dah by H&E staining. Scale bars = 50 μm. **(F)** Fertilization rate of sperm from WT XY, XY *amhy*^-^;*cyp19a1a*^-/-^;*cyp19a1b*^-/-^ and XX/XY *gsdf*^-/-^;*cyp19a1a*^-/-^;*cyp19a1b*^-/-^ males at 180 dah (n = 3 for each genotype). Sg, spermatogonia. Sc, spermatocyte. Sz, spermatozoa. Oc, oocyte. Ocv, ovarian cavity. dah, days after hatching.
(TIF)

S10 Fig Knockdown of *foxl2* by TALENs in the *amhy* and *gsdf* single mutants. **(A)** The mixture of *foxl2* left and right TALENs mRNA was injected into fertilized eggs from *amhy*^−^ XY neo-females crossed with XX neo-males or from *gsdf*^+/-^ XX females crossed with *gsdf*^-/-^ XY males. DNA fragments spanning the target were amplified for mutation analysis. The mutations induced in *foxl2* by TALENs were assayed by restriction enzyme assay as reported previously [56]. The undigested band indicated by arrow head suggested mutations in the target. WT, wild-type. **(B)** Histological examination of gonad sections from WT XX and XX *foxl2*^KD^ mutants at 60 dah by H&E staining. The XX F0 *foxl2* mutant fish showed testicular development, while the WT XX fish showed ovarian development with follicles at 60 dah. Scale bars = 40 μm. **(C)** Detection of *foxl2* mutations in F0 fish by restriction enzyme assay. The XY *amhy*^-^ or XX/XY *gsdf*^-/-^ mutants were selected for *foxl2* mutation analysis. The arrow head indicated the undigested bands. The numbers above the gel lanes indicate the fish with high mutation rate of *foxl2*. M, DNA marker. **(D)** Statistical analysis of the gonadal phenotypes of *amhy* and *gsdf* mutants with different *foxl2* mutation rates. The *foxl2* mutation rate in S10C Fig was calculated by quantifying intensity of uncleaved band. The number in the circle is corresponding to the number in S10C Fig. **(E)** Histological examination of gonads from XY *amhy*^−^, XY *amhy*^−^;*foxl2*^KD^, XY *gsdf*^-/-^, XX/XY *gsdf*^-/-^;*foxl2*^KD^ tilapia at 120 dah by H&E staining. Scale bars = 40 μm. Oc, oocyte. Sg, spermatogonia. Sc, spermatocyte. Sz, spermatozoa. Ed, efferent duct. KD, knockdown. dah, days after hatching.
(TIF)

S11 Fig Sperm morphology and motility analyses of XX *foxl3*^-/-^ mutants. Sperm of WT XY and XX *foxl3*^-/-^ fish were labeled by PKH26 in red. Dynamic trajectory curve of the sperm of WT XY and XX *foxl3*^-/-^ fish. The pink line represents the dynamic trajectory curve of normal sperm. The blue line and green line represent the dynamic trajectory curve of abnormal sperm. Scale bars = 10 μm. dah, days after hatching. WT, wild-type.
(TIF)

S12 Fig The indispensable role of Dmrt1 in tilapia sex determination and testicular development. (A) Once *dmrt1* was mutated, the gonad could not be rescued to a functional testis by mutation of female pathway gene identified so far. In male pathway genes *amhy* and *gsdf* mutants, *cyp19a1a*/estrogen was up-regulated to repress *dmrt1* expression and promote female fate. In *dmrt1* mutants, *cyp19a1a*/estrogen was up-regulated to induce ovary development. In *dmrt1*;*cyp19a1a* double mutants, the gonad developed either as ovary with vitellogenesis or underdeveloped testis with no germ cell. In *dmrt1*;*cyp19a1a*;*cyp19a1b* triple mutants, the gonad developed as ovary with previtellogenic follicles. In *dmrt1*;*cyp19a1a*;*foxl3* triple and AI treated-*dmrt1*;*foxl3* double mutants, the gonad developed eventually as ovary. In the *dmrt1*;*foxl2* double mutants, the gonad developed as underdeveloped testis with no germ cell. (B) Once *dmrt1* was present, sex reversal caused by mutation of other male pathway genes *amhy* and *gsdf* could be rescued. In female pathway genes *foxl2* and *cyp19a1a/cyp19a1b* mutants, estrogen was down-regulated and *dmrt1* was up-regulated to promote male fate. In *amhy*;*cyp19a1a*, *gsdf*;*cyp19a1a*, *amhy*;*cyp19a1a*;*cyp19a1b*, *gsdf*;*cyp19a1a*;*cyp19a1b* mutants, the gonad developed as functional testis due to up-regulation of *dmrt1* in somatic and germ cell. In *foxl3* single, *amhy*;*foxl3* and *gsdf*;*foxl3* double mutants, spermatogenesis occurs in ovarian environment due to up-regulation of *dmrt1* in germ cell. KO, knockout. DKO, double knockout. TKO, triple knockout. E2, 17β-estradiol. AI, aromatase inhibitor. dah, days after hatching.
(TIF)

S13 Fig Establishment of double and triple mutants used in this study. (TIF)

S1 Table Primers used in this study. (DOC)

S2 Table Antibodies used in this study. (DOC)

S1 Data Source data. (XLSX)

## References

[1] Capel B. Vertebrate sex determination: evolutionary plasticity of a fundamental switch. Nat Rev Genet. 2017; 18(11):675–689. doi: 10.1038/nrg.2017.60 . 28804140

[2] Nagahama Y, Chakraborty T, Paul-Prasanth B, Ohta K, Nakamura M. Sex determination, gonadal sex differentiation, and plasticity in vertebrate species. Physiol Rev. 2021; 101(3):1237–1308. doi: 10.1152/physrev.00044.2019 . 33180655

[3] Matsuda M, Nagahama Y, Shinomiya A, Sato T, Matsuda C, Kobayashi T, Morrey CE, Shibata N, Asakawa S, Shimizu N, Hori H, Hamaguchi S, Sakaizumi M. DMY is a Y-specific DM-domain gene required for male development in the medaka fish. Nature. 2002; 417(6888):559–63. doi: 10.1038/nature751 . 12037570

[4] Nanda I, Kondo M, Hornung U, Asakawa S, Winkler C, Shimizu A, Shan Z, Haaf T, Shimizu N, Shima A, Schmid M, Schartl M. A duplicated copy of DMRT1 in the sex-determining region of the Y chromosome of the medaka, *Oryzias latipes*. Proc Natl Acad Sci U S A. 2002; 99(18):11778–83. doi: 10.1073/pnas.182314699 . 12193652 PMC129345

[5] Kitano J, Ansai S, Takehana Y, Yamamoto Y. Diversity and convergence of sex determination mechanisms in teleost fish. Annu Rev Anim Biosci. 2024; 15(12):233–59. doi: 10.1146/annurev-animal-021122-113935 . 37863090

[6] Sinclair AH, Berta P, Palmer MS, Hawkins JR, Griffiths BL, Smith MJ, Foster JW, Frischauf AM, Lovell-Badge R, Goodfellow PN. A gene from the human sex-determining region encodes a protein with homology to a conserved DNA-binding motif. Nature. 1990; 346(6281):240–4. doi: 10.1038/346240a0 . 1695712

[7] Koopman P, Münsterberg A, Capel B, Vivian N, Lovell-Badge R. Expression of a candidate sex-determining gene during mouse testis differentiation. Nature. 1990; 348(6300):450–2. doi: 10.1038/348450a0 . 2247150

[8] Pan Q, Kay T, Depincé A, Adolfi M, Schartl M, Guiguen Y, Herpin A. Evolution of master sex determiners: TGF-β signaling pathways at regulatory crossroads. Philos Trans R Soc Lond B Biol Sci. 2021; 376(1832):20200091. doi: 10.1098/rstb.2020.0091 . 34247498 PMC8273507

[9] Li M, Sun Y, Zhao J, Shi H, Zeng S, Ye K, Jiang D, Zhou L, Sun L, Tao W, Nagahama Y, Kocher TD, Wang D. A tandem duplicate of anti-Müllerian hormone with a missense SNP on the Y chromosome is essential for male sex determination in Nile tilapia, *Oreochromis niloticus*. PLoS Genet. 2015; 11(11):e1005678. doi: 10.1371/journal.pgen.1005678 . 26588702 PMC4654491

[10] Herpin A, Schartl M. Plasticity of gene-regulatory networks controlling sex determination: of masters, slaves, usual suspects, newcomers, and usurpators. EMBO Rep. 2015; 16(10):1260–74. doi: 10.15252/embr.201540667 . 26358957 PMC4766460

[11] Curzon AY, Shirak A, Ron M, Seroussi E. Master-key regulators of sex determination in fish and other vertebrates-a review. Int J Mol Sci. 2023; 24(3):2468. doi: 10.3390/ijms24032468 . 36768795 PMC9917144

[12] Jiang D, Yang H, Li M, Shi H, Zhang X, Wang D. *gsdf* is a downstream gene of *dmrt1* that functions in the male sex determination pathway of the Nile tilapia. Mol Reprod Dev. 2016; 83(6):497–508. doi: 10.1002/mrd.22642 . 27027772

[13] Zhang X, Li M, Ma H, Liu X, Shi H, Li M, Wang D. Mutation of *foxl2* or *cyp19a1a* results in female to male sex reversal in XX Nile tilapia. Endocrinology. 2017; 158(8):2634–2647. doi: 10.1210/en.2017-00127 . 28838139

[14] Dai S, Qi S, Wei X, Liu X, Li Y, Zhou X, Xiao H, Lu B, Wang D, Li M. Germline sexual fate is determined by the antagonistic action of *dmrt1* and *foxl3/foxl2* in tilapia. Development. 2021; 148(8):dev199380. doi: 10.1242/dev.199380 . 33741713

[15] Zhou L, Li M, Wang D. Role of sex steroids in fish sex determination and differentiation as revealed by gene editing. Gen Comp Endocrinol. 2021; 313:113893. doi: 10.1016/j.ygcen.2021.113893 . 34454946

[16] Simpson ER, Mahendroo MS, Means GD, Kilgore MW, Hinshelwood MM, Graham-Lorence S, Amarneh B, Ito Y, Fisher CR, Michael MD. Aromatase cytochrome P450, the enzyme responsible for estrogen biosynthesis. Endocr Rev. 1994; 15(3):342–55. doi: 10.1210/edrv-15-3-342 . 8076586

[17] Guiguen Y, Fostier A, Piferrer F, Chang CF. Ovarian aromatase and estrogens: a pivotal role for gonadal sex differentiation and sex change in fish. Gen Comp Endocrinol. 2010; 165(3):352–66. doi: 10.1016/j.ygcen.2009.03.002 . 19289125

[18] Li M, Sun L, Wang D. Roles of estrogens in fish sexual plasticity and sex differentiation. Gen Comp Endocrinol. 2019; 277:9–16. doi: 10.1016/j.ygcen.2018.11.015 . 30500373

[19] Lau ES, Zhang Z, Qin M, Ge W. Knockout of zebrafish ovarian aromatase gene (*cyp19a1a*) by TALEN and CRISPR/Cas9 leads to all-male offspring due to failed ovarian differentiation. Sci Rep. 2016; 6:37357. doi: 10.1038/srep37357 . 27876832 PMC5120357

[20] Nakamoto M, Shibata Y, Ohno K, Usami T, Kamei Y, Taniguchi Y, Todo T, Sakamoto T, Young G, Swanson P, Naruse K, Nagahama Y. Ovarian aromatase loss-of-function mutant medaka undergo ovary degeneration and partial female-to-male sex reversal after puberty. Mol Cell Endocrinol. 2018; 460:104–122. doi: 10.1016/j.mce.2017.07.013 . 28711606

[21] Wang D, Kobayashi T, Zhou L, Paul-Prasanth B, Ijiri S, Sakai F, Okubo K, Morohashi K, Nagahama Y. Foxl2 up-regulates aromatase gene transcription in a female-specific manner by binding to the promoter as well as interacting with ad4 binding protein/steroidogenic factor 1. Mol Endocrinol. 2007; 21(3):712–25. doi: 10.1210/me.2006-0248 . 17192407

[22] Yang Y, Wang Y, Li Z, Zhou L, Gui J. Sequential, divergent, and cooperative requirements of Foxl2a and Foxl2b in ovary development and maintenance of zebrafish. Genetics. 2017; 205(4):1551–1572. doi: 10.1534/genetics.116.199133 . 28193729 PMC5378113

[23] Gan R, Wang Y, Li Z, Yu Z, Li X, Tong J, Wang Z, Zhang X, Zhou L, Gui J. Functional divergence of multiple duplicated Foxl2 homeologs and alleles in a recurrent polyploid fish. Mol Biol Evol. 2021; 38(5):1995–2013. doi: 10.1093/molbev/msab002 . 33432361 PMC8097289

[24] Bertho S, Pasquier J, Pan Q, Le Trionnaire G, Bobe J, Postlethwait JH, Pailhoux E, Schartl M, Herpin A, Guiguen Y. Foxl2 and its relatives are evolutionary conserved players in gonadal sex differentiation. Sex Dev. 2016; 10(3):111–29. doi: 10.1159/000447611 . 27441599

[25] Geraldo MT, Valente GT, Braz ASK, Martins C. The discovery of Foxl2 paralogs in chondrichthyan, coelacanth and tetrapod genomes reveals an ancient duplication in vertebrates. Heredity. 2013; 111(1):57–65. doi: 10.1038/hdy.2013.19 . 23549337 PMC3692314

[26] Crespo B, Lan-Chow-Wing O, Rocha A, Zanuy S, Gomez A. *foxl2* and *foxl3* are two ancient paralogs that remain fully functional in teleosts. Gen Comp Endocrinol. 2013; 194:81–93. doi: 10.1016/j.ygcen.2013.08.016 . 24045113

[27] Nishimura T, Sato T, Yamamoto Y, Watakabe I, Ohkawa Y, Suyama M, Kobayashi S, Tanaka M. Sex determination. *foxl3* is a germ cell-intrinsic factor involved in sperm-egg fate decision in medaka. Science. 2015; 349(6245):328–31. doi: 10.1126/science.aaa2657 . 26067255

[28] Liu Y, Kossack ME, McFaul ME, Christensen LN, Siebert S, Wyatt SR, Kamei CN, Horst S, Arroyo N, Drummond IA, Juliano CE, Draper BW. Single-cell transcriptome reveals insights into the development and function of the zebrafish ovary. Elife. 2022; 11:e76014. doi: 10.7554/eLife.76014 . 35588359 PMC9191896

[29] Raymond CS, Murphy MW, O’Sullivan MG, Bardwell VJ, Zarkower D. *Dmrt1*, a gene related to worm and fly sexual regulators, is required for mammalian testis differentiation. Genes Dev. 2000; 14(20):2587–95. doi: 10.1101/gad.834100 . 11040213 PMC316999

[30] Zhao L, Svingen T, Ng ET, Koopman P. Female-to-male sex reversal in mice caused by transgenic overexpression of Dmrt1. Development. 2015; 142(6):1083–8. doi: 10.1242/dev.122184 . 25725066

[31] Dujardin E, André M, Dewaele A, Mandon-Pépin B, Poulat F, Frambourg A, Thépot D, Jouneau L, Jolivet G, Pailhoux E, Pannetier M. *DMRT1* is a testis-determining gene in rabbits and is also essential for female fertility. Elife. 2023; 12:RP89284. doi: 10.7554/eLife.89284 . 37847154 PMC10581690

[32] Ioannidis J, Taylor G, Zhao D, Liu L, Idoko-Akoh A, Gong D, Lovell-Badge R, Guioli S, McGrew MJ, Clinton M. Primary sex determination in birds depends on DMRT1 dosage, but gonadal sex does not determine adult secondary sex characteristics. Proc Natl Acad Sci U S A. 2021; 118(10):e2020909118. doi: 10.1073/pnas.2020909118 . 33658372 PMC7958228

[33] Ge C, Ye J, Zhang H, Zhang Y, Sun W, Sang Y, Capel B, Qian G. Dmrt1 induces the male pathway in a turtle species with temperature-dependent sex determination. Development. 2017; 144(12):2222–2233. doi: 10.1242/dev.152033 . 28506988

[34] Webster KA, Schach U, Ordaz A, Steinfeld JS, Draper BW, Siegfried KR. Dmrt1 is necessary for male sexual development in zebrafish. Dev Biol. 2017; 422(1):33–46. doi: 10.1016/j.ydbio.2016.12.008 . 27940159 PMC5777149

[35] Masuyama H, Yamada M, Kamei Y, Fujiwara-Ishikawa T, Todo T, Nagahama Y, Matsuda M. *Dmrt1* mutation causes a male-to-female sex reversal after the sex determination by Dmy in the medaka. Chromosome Res. 2012; 20(1):163–76. doi: 10.1007/s10577-011-9264-x . 22187367

[36] Smith CA, Roeszler KN, Ohnesorg T, Cummins DM, Farlie PG, Doran TJ, Sinclair AH. The avian Z-linked gene DMRT1 is required for male sex determination in the chicken. Nature. 2009; 461(7261):267–71. doi: 10.1038/nature08298 . 19710650

[37] Cui Z, Liu Y, Wang W, Wang Q, Zhang N, Lin F, Wang N, Shao C, Dong Z, Li Y, Yang Y, Hu M, Li H, Gao F, Wei Z, Meng L, Liu Y, Wei M, Zhu Y, Guo H, Cheng CH, Schartl M, Chen S. Genome editing reveals dmrt1 as an essential male sex-determining gene in Chinese tongue sole (*Cynoglossus semilaevis*). Sci Rep. 2017; 7:42213. doi: 10.1038/srep42213 . 28205594 PMC5311979

[38] Wang L, Sun F, Wan ZY, Yang Z, Tay YX, Lee M, Ye B, Wen Y, Meng Z, Fan B, Alfiko Y, Shen Y, Piferrer F, Meyer A, Schartl M, Yue GH. Transposon-induced epigenetic silencing in the X chromosome as a novel form of *dmrt1* expression regulation during sex determination in the fighting fish. BMC Biol. 2022; 20(1):5. doi: 10.1186/s12915-021-01205-y . 34996452 PMC8742447

[39] Romano S, Kaufman OH, Marlow FL. Loss of *dmrt1* restores zebrafish female fates in the absence of *cyp19a1a* but not *rbpms2a/b*. Development. 2020; 147(18):dev190942. doi: 10.1242/dev.190942 . 32895289 PMC7541348

[40] Wu K, Song W, Zhang Z, Ge W. Disruption of *dmrt1* rescues the all-male phenotype of the *cyp19a1a* mutant in zebrafish—a novel insight into the roles of aromatase/estrogens in gonadal differentiation and early folliculogenesis. Development. 2020; 147(4):dev182758. doi: 10.1242/dev.182758 . 32001440

[41] Chang X, Kobayashi T, Senthilkumaran B, Kobayashi-Kajura H, Sudhakumari CC, Nagahama Y. Two types of aromatase with different encoding genes, tissue distribution and developmental expression in Nile tilapia (*Oreochromis niloticus*). Gen Comp Endocrinol. 2005; 141(2):101–15. doi: 10.1016/j.ygcen.2004.11.020 . 15748711

[42] Zhang X, Min Q, Li M, Liu X, Li M, Wang D. Mutation of cyp19a1b results in sterile males due to efferent duct obstruction in Nile tilapia. Mol Reprod Dev. 2019; 86(9):1224–1235. doi: 10.1002/mrd.23237 . 31339195

[43] Liu X, Dai S, Wu J, Wei X, Zhou X, Chen M, Tan D, Pu D, Li M, Wang D. Roles of anti-Müllerian hormone and its duplicates in sex determination and germ cell proliferation of Nile tilapia. Genetics. 2022; 220(3):iyab237. doi: 10.1093/genetics/iyab237 . 35100374 PMC9208641

[44] Wang M, Li Z, Ding M, Yao T, Yang S, Zhang X, Miao C, Du W, Shi Q, Li S, Mei J, Wang Y, Wang Z, Zhou L, Li X, Gui J. Two duplicated gsdf homeologs cooperatively regulate male differentiation by inhibiting cyp19a1a transcription in a hexaploid fish. PLoS Genet. 2022; 18(6):e1010288. doi: 10.1371/journal.pgen.1010288 . 35767574 PMC9275722

[45] Yamaguchi T, Kitano T. Amh/Amhr2 signaling causes masculinization by inhibiting estrogen synthesis during gonadal sex differentiation in Japanese Flounder (*Paralichthys olivaceus*). Int J Mol Sci. 2023; 24(3):2480. doi: 10.3390/ijms24032480 . 36768803 PMC9917198

[46] Paul-Prasanth B, Bhandari RK, Kobayashi T, Horiguchi R, Kobayashi Y, Nakamoto M, Shibata Y, Sakai F, Nakamura M, Nagahama Y. Estrogen oversees the maintenance of the female genetic program in terminally differentiated gonochorists. Sci Rep. 2013; 3:2862. doi: 10.1038/srep02862 . 24096556 PMC3791451

[47] Takatsu K, Miyaoku K, Roy SR, Murono Y, Sago T, Itagaki H, Nakamura M, Tokumoto T. Induction of female-to-male sex change in adult zebrafish by aromatase inhibitor treatment. Sci Rep. 2013; 3:3400. doi: 10.1038/srep03400 . 24292399 PMC3844967

[48] Sun L, Jiang X, Xie Q, Yuan J, Huang B, Tao W, Zhou L, Nagahama Y, Wang D. Transdifferentiation of differentiated ovary into functional testis by long-term treatment of aromatase inhibitor in Nile tilapia. Endocrinology. 2014; 155(4):1476–88. doi: 10.1210/en.2013-1959 . 24437491

[49] Li M, Sun L, Zhou L, Wang D. Tilapia, a good model for studying reproductive endocrinology. Gen Comp Endocrinol. 2024; 345:114395. doi: 10.1016/j.ygcen.2023.114395 . 37879418

[50] Ijiri S, Kaneko H, Kobayashi T, Wang DS, Sakai F, Paul-Prasanth B, Nakamura M, Nagahama Y. Sexual dimorphic expression of genes in gonads during early differentiation of a teleost fish, the Nile tilapia *Oreochromis niloticus*. Biol Reprod. 2008; 78(2):333–41. doi: 10.1095/biolreprod.107.064246 . 17942796

[51] Tao W, Yuan J, Zhou L, Sun L, Sun Y, Yang S, Li M, Zeng S, Huang B, Wang D. Characterization of gonadal transcriptomes from Nile tilapia (Oreochromis niloticus) reveals differentially expressed genes. PLoS One. 2013; 8(5):e63604. doi: 10.1371/journal.pone.0063604 . 23658843 PMC3643912

[52] Kobayashi T, Kajiura-Kobayashi H, Nagahama Y. Differential expression of vasa homologue gene in the germ cells during oogenesis and spermatogenesis in a teleost fish, tilapia, *Oreochromis niloticus*. Mech Dev. 2000; 99(1–2):139–42. doi: 10.1016/s0925-4773(00)00464-0 . 11091081

[53] Wang D, Zhou L, Kobayashi T, Matsuda M, Shibata Y, Sakai F, Nagahama Y. Doublesex- and Mab-3-related transcription factor-1 repression of aromatase transcription, a possible mechanism favoring the male pathway in tilapia. Endocrinology. 2010; 151(3):1331–40. doi: 10.1210/en.2009-0999 . 20056824

[54] Suzuki A, Tanaka M, Shibata N, Nagahama Y. Expression of aromatase mRNA and effects of aromatase inhibitor during ovarian development in the medaka, *Oryzias latipes*. J Exp Zool A Comp Exp Biol. 2004; 301(3):266–73. doi: 10.1002/jez.a.20027 . 14981786

[55] Wei L, Tang Y, Zeng X, Li Y, Zhang S, Deng L, Wang L, Wang D. The transcription factor Sox30 is involved in Nile tilapia spermatogenesis. J Genet Genomics. 2022; 49(7):666–676. doi: 10.1016/j.jgg.2021.11.003 . 34801758

[56] Li M, Yang H, Li M, Sun Y, Jiang X, Xie Q, Wang T, Shi H, Sun L, Zhou L, Wang D. Antagonistic roles of Dmrt1 and Foxl2 in sex differentiation via estrogen production in tilapia as demonstrated by TALENs. Endocrinology. 2013; 154(12):4814–25. doi: 10.1210/en.2013-1451 . 24105480

[57] Li X, Mei J, Ge C, Liu X, Gui J. Sex determination mechanisms and sex control approaches in aquaculture animals. Sci China Life Sci. 2022; 65(6):1091–1122. doi: 10.1007/s11427-021-2075-x . 35583710

[58] Mustapha UF, Huang Y, Huang Y, Assan D, Shi H, Jiang M, Deng S, Li G, Jiang D. Gonadal development and molecular analysis revealed the critical window for sex differentiation, and E-2 reversibility of XY-male spotted scat, *Scatophagus argus*. Aquaculture. 2021; 544:737147. doi: 10.1016/j.aquaculture.2021.737147

[59] Bhandari RK, Nakamura M, Kobayashi T, Nagahama Y. Suppression of steroidogenic enzyme expression during androgen-induced sex reversal in Nile tilapia (*Oreochromis niloticus*). Gen Comp Endocrinol. 2006; 145(1):20–4. doi: 10.1016/j.ygcen.2005.06.014 . 16115634

[60] Minkina A, Matson CK, Lindeman RE, Ghyselinck NB, Bardwell VJ, Zarkower D. DMRT1 protects male gonadal cells from retinoid-dependent sexual transdifferentiation. Dev Cell. 2014; 29(5):511–520. doi: 10.1016/j.devcel.2014.04.017 . 24856513 PMC4105363

[61] Zhang T, Oatley J, Bardwell VJ, Zarkower D. DMRT1 is required for mouse spermatogonial stem cell maintenance and replenishment. PLoS Genet. 2016; 12(9):e1006293. doi: 10.1371/journal.pgen.1006293 . 27583450 PMC5008761

[62] Dranow DB, Hu K, Bird AM, Lawry ST, Adams MT, Sanchez A, Amatruda JF, Draper BW. Bmp15 is an oocyte-produced signal required for maintenance of the adult female sexual phenotype in zebrafish. PLoS Genet. 2016; 12(9):e1006323. doi: 10.1371/journal.pgen.1006323 . 27642754 PMC5028036

[63] Kaufman OH, Lee K, Martin M, Rothhämel S, Marlow FL. rbpms2 functions in Balbiani body architecture and ovary fate. PLoS Genet. 2018; 14(7):e1007489. doi: 10.1371/journal.pgen.1007489 . 29975683 PMC6049948

[64] Fan S, Shi H, Peng Y, Huang Y, Jiang M, Li G, Wang D, Jiang D. Dietary aromatase inhibitor treatment converts XY gsdf homozygous mutants to sub-fertile male in Nile tilapia (Oreochromis niloticus). Aquaculture. 2023; 569: 739381. doi: 10.1016/j.aquaculture.2023.739381

[65] Jiang D, Mustapha UF, Shi H, Huang Y, Si-Tu JX, Wang M, Deng S, Chen H, Tian C, Zhu C, Li M, Li G. Expression and transcriptional regulation of gsdf in spotted scat (*Scatophagus argus*). Comp Biochem Physiol B Biochem Mol Biol. 2019; 233:35–45. doi: 10.1016/j.cbpb.2019.04.002 . 30980893

[66] Krentz AD, Murphy MW, Kim S, Cook MS, Capel B, Zhu R, Matin A, Sarver AL, Parker KL, Griswold MD, Looijenga LH, Bardwell VJ, Zarkower D. The DM domain protein DMRT1 is a dose-sensitive regulator of fetal germ cell proliferation and pluripotency. Proc Natl Acad Sci U S A. 2009; 106(52):22323–8. doi: 10.1073/pnas.0905431106 . 20007774 PMC2799724

[67] Raghuveer K, Senthilkumaran B. Identification of multiple dmrt1s in catfish: localization, dimorphic expression pattern, changes during testicular cycle and after methyltestosterone treatment. J Mol Endocrinol. 2009; 42(5):437–48. doi: 10.1677/JME-09-0011 . 19293249

[68] Jeng SR, Wu GC, Yueh WS, Kuo SF, Dufour S, Chang CF. Dmrt1 (doublesex and mab-3-related transcription factor 1) expression during gonadal development and spermatogenesis in the Japanese eel. Gen Comp Endocrinol. 2019; 279:154–163. doi: 10.1016/j.ygcen.2019.03.012 . 30902612

[69] Wang D, Kobayashi T, Zhou L, Nagahama Y. Molecular cloning and gene expression of Foxl2 in the Nile tilapia, *Oreochromis niloticus*. Biochem Biophys Res Commun. 2004; 320(1):83–9. doi: 10.1016/j.bbrc.2004.05.133 . 15207705

[70] Cao J, Cao Z, Wu T. Generation of antibodies against DMRT1 and DMRT4 of *Oreochromis aurea* and analysis of their expression profile in *Oreochromis aurea* tissues. J Genet Genomics. 2007; 34(6):497–509. doi: 10.1016/S1673-8527(07)60055-1 . 17601609

[71] Burtis KC, Baker BS. Drosophila doublesex gene controls somatic sexual differentiation by producing alternatively spliced mRNAs encoding related sex-specific polypeptides. Cell. 1989; 56(6):997–1010. doi: 10.1016/0092-8674(89)90633-8 . 2493994

[72] Yi W, Ross JM, Zarkower D. Mab-3 is a direct tra-1 target gene regulating diverse aspects of C. elegans male sexual development and behavior. Development. 2000; 127(20):4469–80. doi: 10.1242/dev.127.20.4469 . 11003845

[73] Shen MM, Hodgkin J. mab-3, a gene required for sex-specific yolk protein expression and a male-specific lineage in C. elegans. Cell. 1988; 54(7):1019–31. doi: 10.1016/0092-8674(88)90117-1 . 3046751

[74] Ohbayashi F, Suzuki MG, Mita K, Okano K, Shimada T. A homologue of the *Drosophila* doublesex gene is transcribed into sex-specific mRNA isoforms in the silkworm, *Bombyx mori*. Comp Biochem Physiol B Biochem Mol Biol. 2001; 128(1):145–58. doi: 10.1016/s1096-4959(00)00304-3 . 11163313

[75] Chang N, Sun C, Gao L, Zhu D, Xu X, Zhu X, Xiong JW, Xi JJ. Genome editing with RNA-guided Cas9 nuclease in zebrafish embryos. Cell Res. 2013; 23(4):465–72. doi: 10.1038/cr.2013.45 . 23528705 PMC3616424

[76] Sun Y, Jiang D, Zeng S, Hu C, Ye K, Yang C, Yang S, Li M, Wang D. Screening and characterization of sex-linked DNA markers and marker-assisted selection in the Nile tilapia (*Oreochromis niloticus*). Aquaculture. 2014; 433: 19–27. doi: 10.1016/j.aquaculture.2014.05.035

[77] Livak KJ, Schmittgen TD. Analysis of relative gene expression data using real-time quantitative PCR and the 2(-Delta Delta C(T)) Method. Methods. 2001; 25(4):402–8. doi: 10.1006/meth.2001.1262 . 11846609

[78] Zheng Q, Xiao H, Shi H, Wang T, Sun L, Tao W, Kocher TD, Li M, Wang D. Loss of Cyp11c1 causes delayed spermatogenesis due to the absence of 11-ketotestosterone. J Endocrinol. 2020; 244(3):487–499. doi: 10.1530/JOE-19-0438 . 31910154

[79] Yu M, Zhang S, Ma Z, Qiang J, Wei J, Sun L, Kocher TD, Wang D, Tao W. Disruption of Zar1 leads to arrested oogenesis by regulating polyadenylation via Cpeb1 in tilapia (*Oreochromis niloticus*). Int J Biol Macromol. 2024; 260(Pt 2):129632. doi: 10.1016/j.ijbiomac.2024.129632 . 38253139

[80] He J, Mo D, Chen J, Luo L. Combined whole-mount fluorescence in situ hybridization and antibody staining in zebrafish embryos and larvae. Nat Protoc. 2020; 15(10):3361–3379. doi: 10.1038/s41596-020-0376-7 . 32908315

[81] Shihan MH, Novo SG, Le Marchand SJ, Wang Y, Duncan MK. A simple method for quantitating confocal fluorescent images. Biochem Biophys Rep. 2021; 25:100916. doi: 10.1016/j.bbrep.2021.100916 . 33553685 PMC7856428

[82] Chen C, Chen H, Zhang Y, Thomas HR, Frank MH, He Y, Xia R. TBtools: An Integrative Toolkit Developed for Interactive Analyses of Big Biological Data. Mol Plant. 2020; 13(8):1194–1202. doi: 10.1016/j.molp.2020.06.009 . 32585190

[83] Zhai G, Shu T, Yu G, Tang H, Shi C, Jia J, Lou Q, Dai X, Jin X, He J, Xiao W, Liu X, Yin Z. Augmentation of progestin signaling rescues testis organization and spermatogenesis in zebrafish with the depletion of androgen signaling. Elife. 2022; 11:e66118. doi: 10.7554/eLife.66118 . 35225789 PMC8912926

[84] Farlora R, Hattori-Ihara S, Takeuchi Y, Hayashi M, Octavera A, Alimuddin, Yoshizaki G. Intraperitoneal germ cell transplantation in the Nile tilapia *Oreochromis niloticus*. Mar Biotechnol (NY). 2014; 16(3):309–20. doi: 10.1007/s10126-013-9551-y . 24096828

