# Supplementary figures and images for: Dmrt1 is the only male pathway gene tested indispensable for sex determination and functional testis development in tilapia
Source: PLoS Genet. 2024 Mar 27;20(3):e1011210. doi: 10.1371/journal.pgen.1011210 (PMC10971778; doi:10.1371/journal.pgen.1011210)

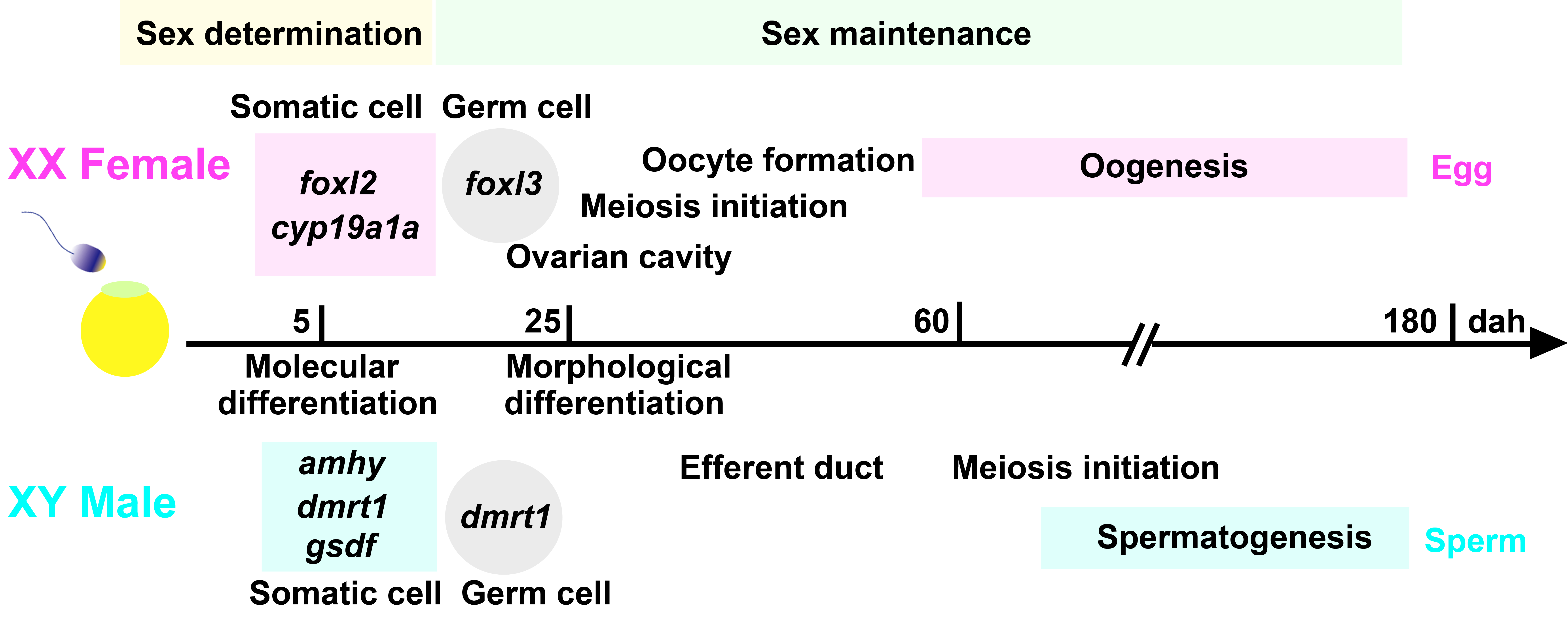

Supplement: S1 Fig — Previous studies demonstrated that a number of genes showed sexually dimorphic expression between XX and XY gonads at 5 dah including amhy, dmrt1, gsdf, foxl2 and cyp19a1a, indicating the critical time for sex determination [50,51]. The first characteristic of gonadal differentiation occurs in tilapia larvae between 20 and 25 dah with appearance of the ovarian cavity in the XX gonad. The efferent duct is observed in the XY testis at around 40 dah. The germ cell meiosis in XX gonads initiates at 25–30 dah and oocyte can be observed thereafter, but germ cells does not initiate meiosis in XY gonads until 60 dah and spermatocyte can be observed [52]. (TIF) [file pgen.1011210.s001.tif]

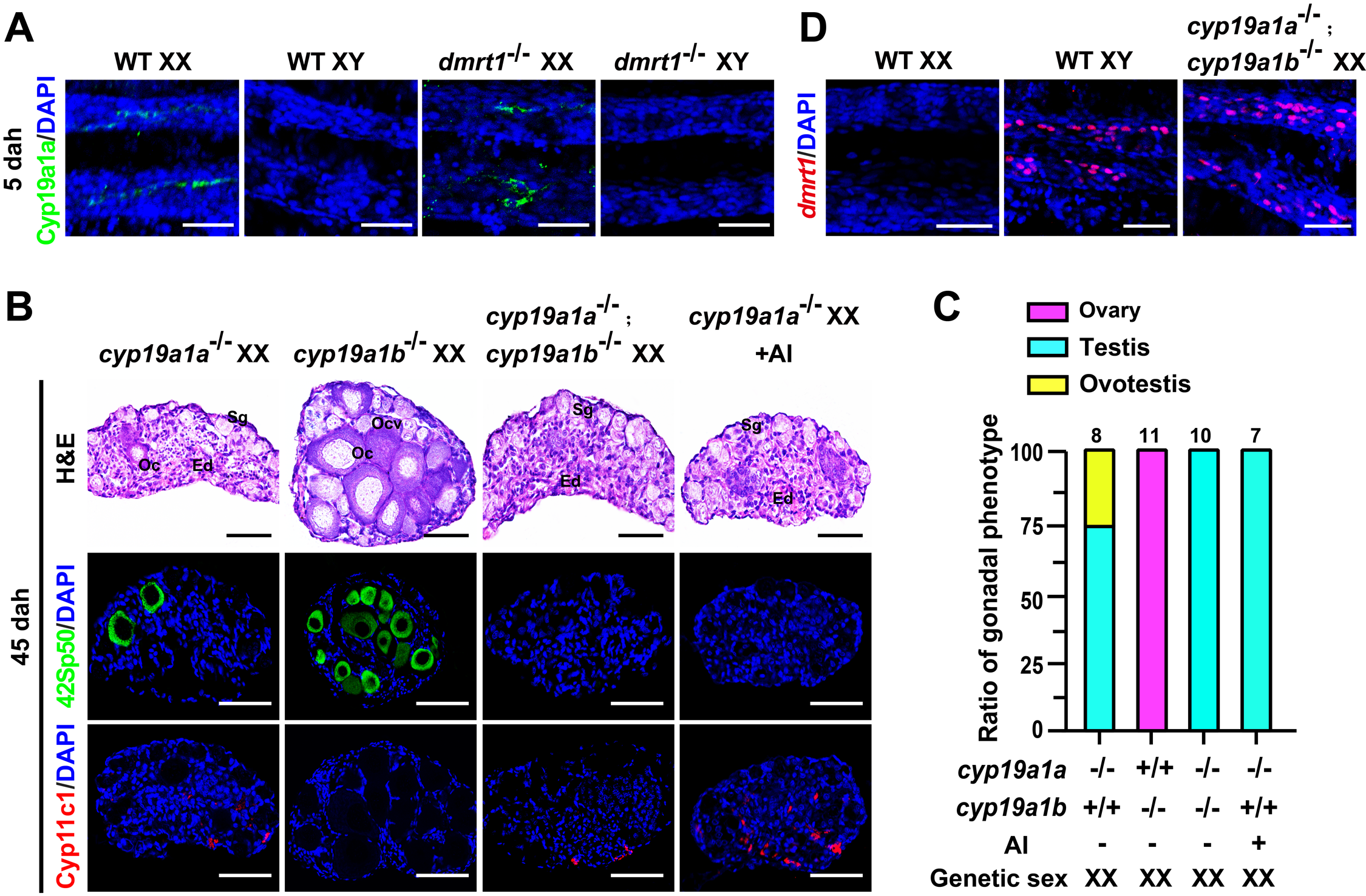

Supplement: S2 Fig — (A) Expression of Cyp19a1a in the gonads of the WT XX, WT XY, XY dmrt1-/- and XX dmrt1-/- fish at 5 dah by Whole-mount IF. Scale bars = 40 μm. (B) Histological examination of gonads from XX cyp19a1a-/-, XX cyp19a1b-/-, XX cyp19a1a-/-;cyp19a1b-/- and AI treated-XX cyp19a1a-/- tilapia at 45 dah using hematoxylin and eosin (H&E) staining. Expressions of Leydig cell marker Cyp11c1 and oocyte marker 42Sp50 were analyzed by immunofluorescence (IF). AI, aromatase inhibitor, Letrozole. Scale bars = 40 μm. (C) Sex ratios in XX cyp19a1a-/-, XX cyp19a1b-/-, XX cyp19a1a-/-;cyp19a1b-/- and AI treated-XX cyp19a1a-/- tilapia at 45 dah. (D) Expression of dmrt1 mRNA in the gonads of the WT XX, WT XY and XX cyp19a1a-/-;cyp19a1b-/- fish at 5 dah by Whole-mount FISH. Nuclei were counterstained with DAPI. Scale bars = 40 μm. Oc, oocyte. Ocv, ovarian cavity. Sg, spermatogonia. Ed, efferent duct. dah, days after hatching. (TIF) [file pgen.1011210.s002.tif]

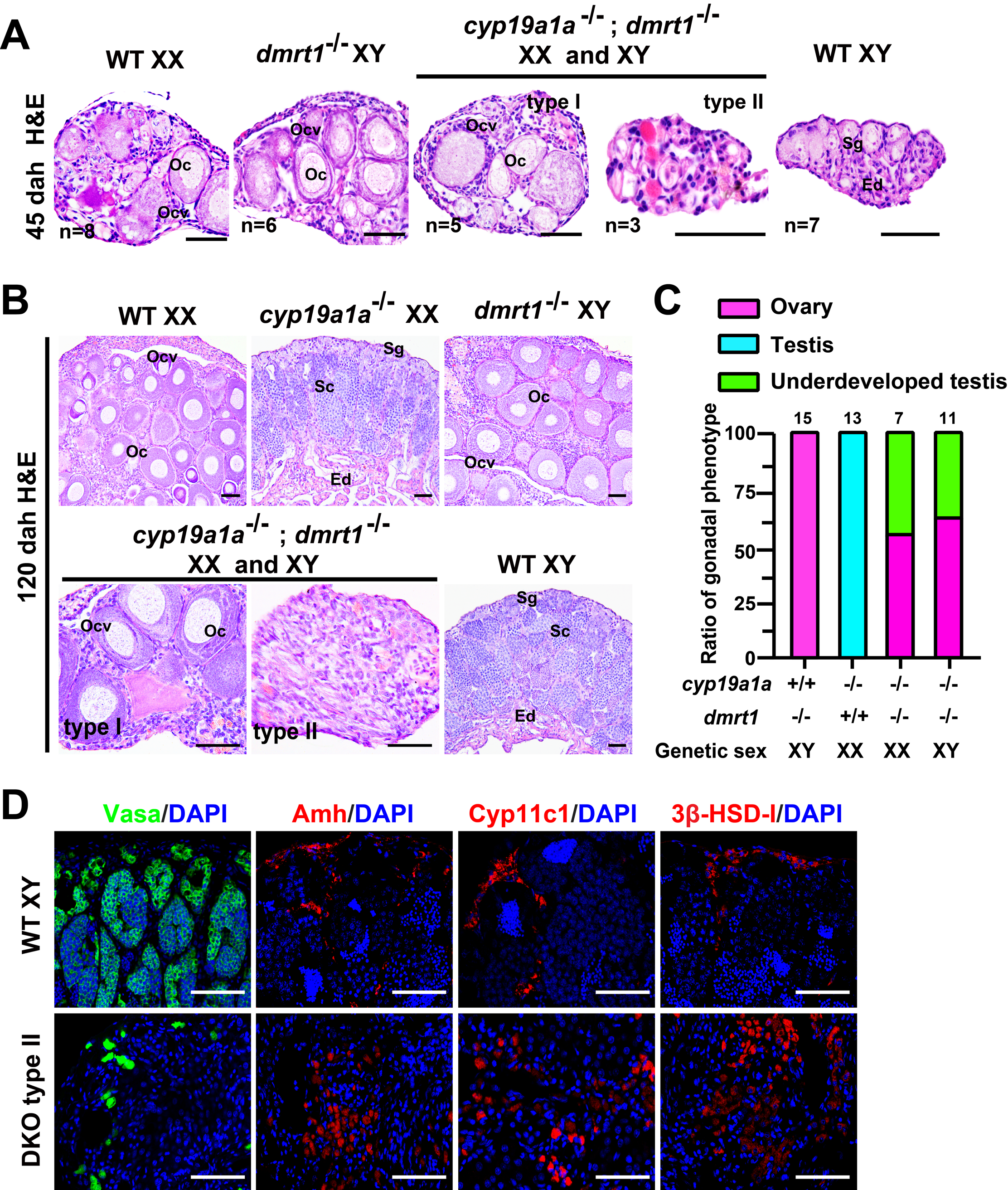

Supplement: S3 Fig — (A) Histological examination of gonads from WT XX, WT XY, XY dmrt1-/- and XX/XY dmrt1-/-;cyp19a1a-/- tilapia at 45 dah by H&E staining. Scale bars = 40 μm. (B) Histological examination of gonads from WT XX, WT XY, XY dmrt1-/-, XX cyp19a1a-/-, XX/XY dmrt1-/-;cyp19a1a-/- tilapia at 120 dah using H&E staining. Oc, oocyte. Ocv, ovarian cavity. Sg, spermatogonia. Sc, spermatocyte. Ed, efferent duct. Scale bars = 40 μm. (C) Sex ratios in XY dmrt1-/-, XX cyp19a1a-/- and XX/XY dmrt1-/-;cyp19a1a-/- tilapia at 120 dah. (D) Gene expressions in the underdeveloped testis of dmrt1-/-;cyp19a1a-/- mutants (type II) detected by IF at 120 dah. Vasa for germ cell. Amh for Sertoli cell. Cyp11c1 and 3β-HSD-I for Leydig cell. Nuclei were counterstained with DAPI. WT, wild-type. dah, days after hatching. Scale bars = 40 μm. (TIF) [file pgen.1011210.s003.tif]

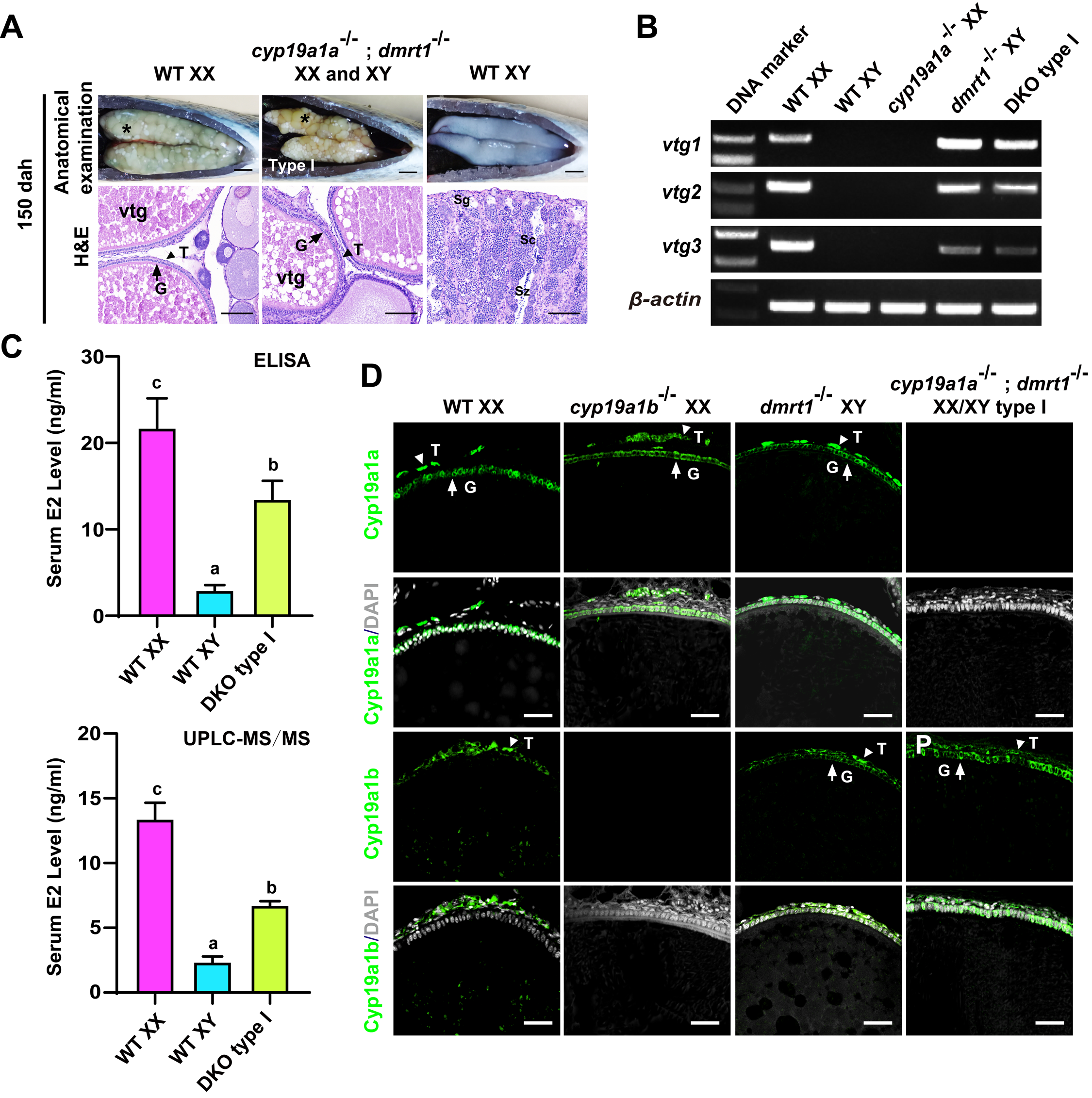

Supplement: S4 Fig — (A) Anatomical examination of the gonads from WT XX, WT XY and XX/XY dmrt1-/-;cyp19a1a-/- type I tilapia at 150 dah. The asterisk indicates ovum. Histological examination of gonads from WT XX, WT XY, XX/XY dmrt1-/-;cyp19a1a-/- type I at 150 dah by H&E staining. Arrow and arrowhead indicates granulosa cell (G) and theca cell (T), respectively. Vtg, vitellogenin. Sg, spermatogonia. Sc, spermatocyte. Sz, spermatozoa. Scale bars = 40 μm. (B) RT-PCR analysis of three vitellogenin (vtg1, vtg2 and vtg3) expressions in the livers of WT XX, WT XY, XY dmrt1-/-, XX cyp19a1a-/- and XX/XY dmrt1-/-;cyp19a1a-/- type I at 150 dah. β-actin was used as an internal control. DKO, XX/XY dmrt1-/-;cyp19a1a-/- double mutants. (C) Serum E2 level in WT XX, WT XY and XX/XY dmrt1-/-;cyp19a1a-/- type I was measured by ELISA and UPLC-MS methods. Data were expressed as the mean ± SD of triplicates. Different letters above the error bars indicate statistical differences at P<0.05 as determined by one-way ANOVA followed by Tukey test. (D) Cellular locations of Cyp19a1a and Cyp19a1b proteins in the ovaries of WT XX, XX cyp19a1b-/-, XY dmrt1-/- and XX/XY dmrt1-/-;cyp19a1a-/- type I double mutants analyzed by IF. Nuclei were counterstained with DAPI. Arrow and arrowhead indicates granulosa cell (G) and theca cell (T), respectively. Scale bars = 40 μm. WT, wild-type. dah, days after hatching. (TIF) [file pgen.1011210.s004.tif]

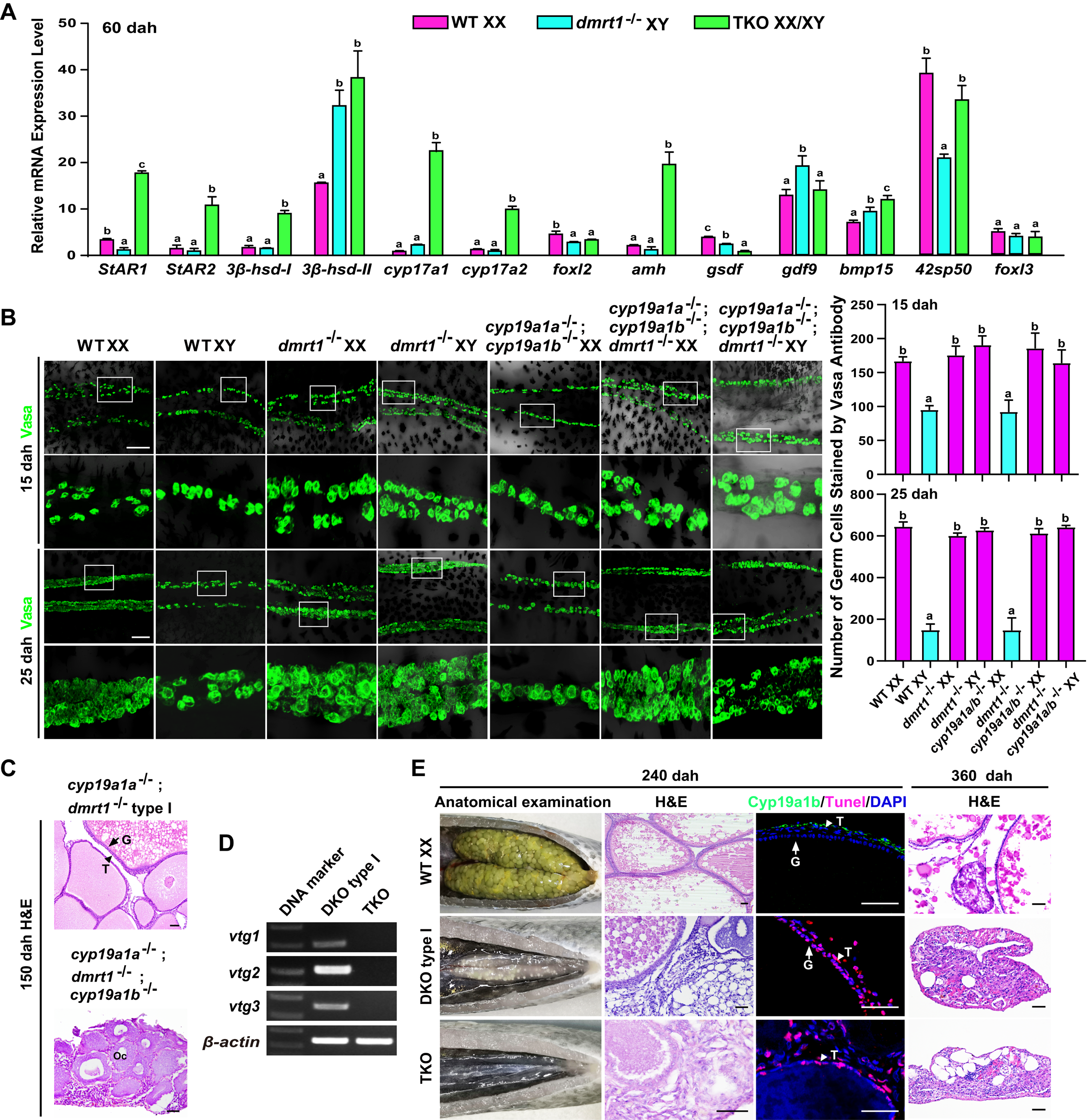

Supplement: S5 Fig — (A) Real-time PCR analysis of genes related to gonadal steroidogenesis, oogonia and oocyte in WT XX, XY dmrt1-/- and XX/XY dmrt1-/-;cyp19a1a-/-;cyp19a1b-/- mutants at 60 dah. β-actin was used as an internal control. Data were expressed as the mean ± SD of triplicates. Different letters above the error bars indicate statistical differences at P<0.05 as determined by one-way ANOVA followed by Tukey test. (B) The germ cells of WT XX, WT XY, XX/XY dmrt1-/-, XX cyp19a1a-/-;cyp19a1b-/- and XX/XY dmrt1-/-;cyp19a1a-/-;cyp19a1b-/- tilapia were stained using Vasa antibody at 15 and 25 dah. The germ cell number in each genotype was counted (n = 3). Scale bars = 200 μm. (C) Histological examination of gonads from dmrt1-/-;cyp19a1a-/- double mutants (type I) and dmrt1-/-;cyp19a1a-/-;cyp19a1b-/- triple mutants at 150 dah by H&E staining. Arrow and arrowhead indicates granulosa cell (G) and theca cell (T), respectively. Oc, oocyte. Scale bars = 40 μm. (D) RT-PCR analysis of vtg1, vtg2 and vtg3 mRNA expressions in the livers of type I dmrt1-/-;cyp19a1a-/- double mutants (DKO type I) and dmrt1-/-;cyp19a1a-/-;cyp19a1b-/- triple mutants (TKO) at 150 dah. β-actin was used as an internal control. (E) Anatomical examination of ovaries from XX/XY DKO type I, XX/XY TKO and WT XX tilapia at 240 dah. Histological examination of the gonads from DKO type I, TKO and WT XX tilapia by H&E staining at 240 and 360 dah. Expression of Cyp19a1b in the gonads of WT XX, DKO type I and TKO tilapia was analyzed by IF. TUNEL analysis showed the apoptosis of granulosa cells and theca cells in the ovaries of DKO type I and TKO mutants, but not in the WT XX at 240 dah. DKO, XX/XY dmrt1-/-;cyp19a1a-/- double mutants; TKO, XX/XY dmrt1-/-;cyp19a1a-/-;cyp19a1b-/- triple mutants. Nuclei were counterstained with DAPI. Scale bars = 40 μm. WT, wild-type. dah, days after hatching. (TIF) [file pgen.1011210.s005.tif]

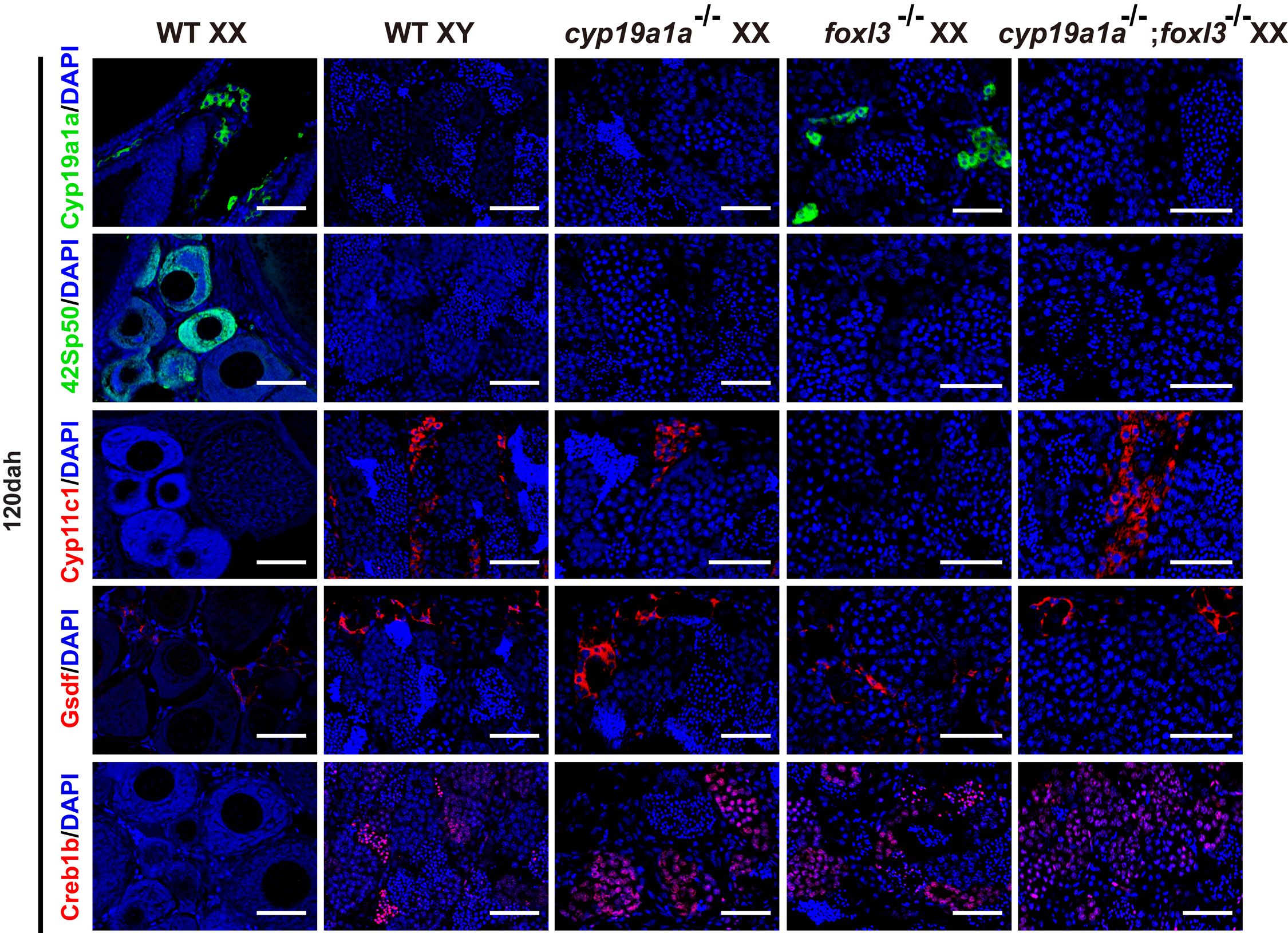

Supplement: S6 Fig — Expression of somatic cell and germ cell markers in the gonads of WT XX, WT XY, XX cyp19a1a-/-, XX foxl3-/- and XX cyp19a1a-/-;foxl3-/- tilapia were analyzed by IF at 120 dah. Cyp19a1a, a female somatic specific marker. 42Sp50, an oocyte marker. Cyp11c1, a Leydig cell marker. Gsdf, a somatic cell marker expressed highly in males compared with females. Creb1b, a spermatocyte marker. Nuclei were counterstained with DAPI. WT, wild-type. dah, days after hatching. Scale bars = 40 μm. (TIF) [file pgen.1011210.s006.tif]

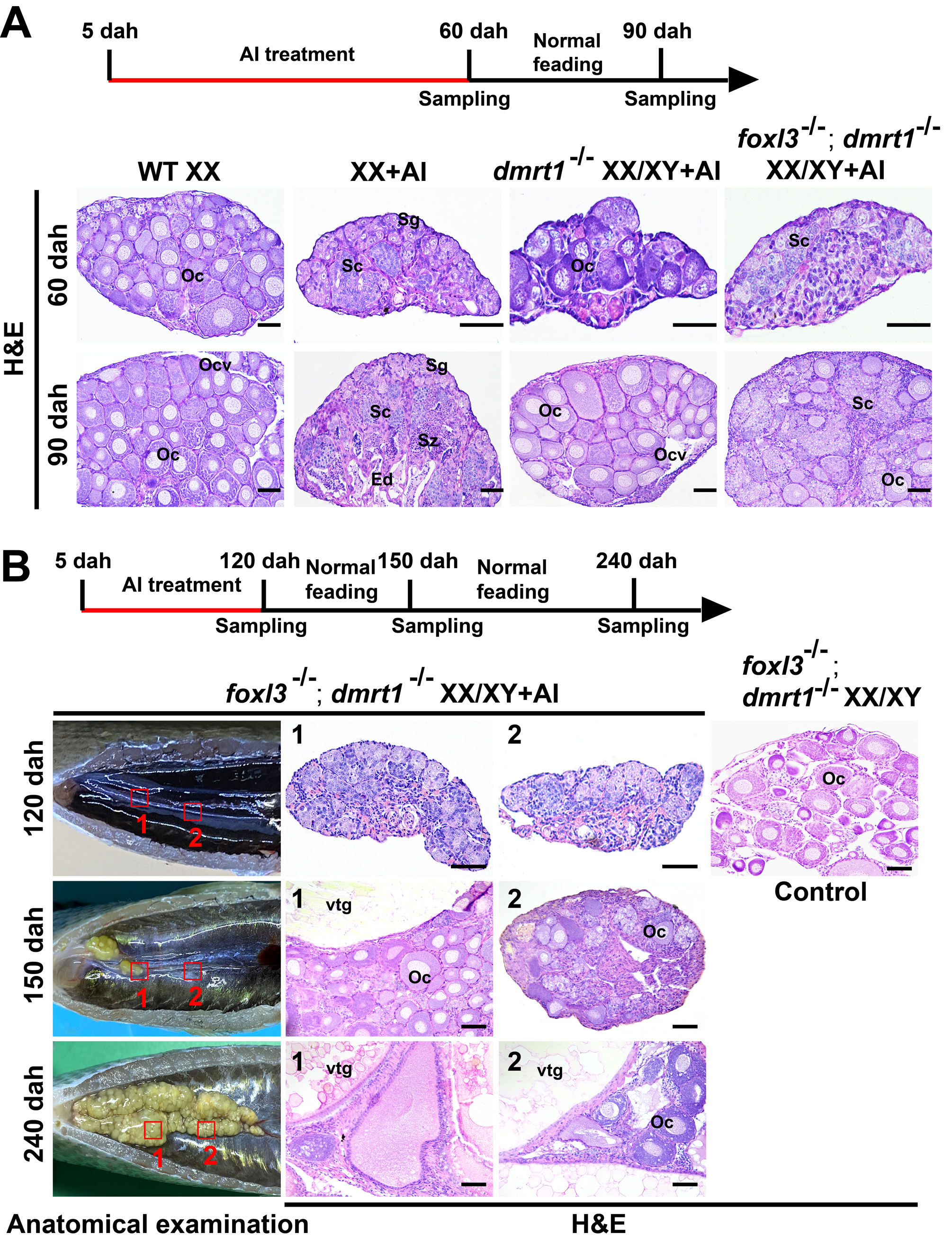

Supplement: S7 Fig — (A) The WT XX, dmrt1-/- single, dmrt1-/-;foxl3-/- double mutants were treated by AI from 5 to 60 dah. The gonadal samples were collected at 60 and 90 dah for histological examination. At 60 dah, the gonads of AI treated-WT XX tilapia displayed testicular development, while the gonads of AI treated-XX/XY dmrt1-/- tilapia still developed as ovaries with oocyte development. The gonads of AI treated-dmrt1-/-;foxl3-/- double mutants exhibited testicular morphology with spermatocyte-like cells at 60 dah. However, at 90 dah, the gonads of AI treated-dmrt1-/-;foxl3-/- double mutants developed as ovotestis with both previtellogenic follicles and spermatocyte-like cells. Scale bars = 50 μm. (B) The dmrt1-/-;foxl3-/- double mutants were treated by AI from 5 to 120 dah. The gonadal samples were collected at 120, 150 and 240 dah for histological examination. At 120 dah, the gonads of AI treated-dmrt1-/-;foxl3-/- double mutants displayed testis morphology with spermatocyte-like cells. However, the gonads of AI treated-dmrt1-/-;foxl3-/- double mutants developed as ovotestis with many previtellogenic follicles at 150 dah. Subsequently, the gonads of AI treated-dmrt1-/-;foxl3-/- double mutants developed as ovaries with a large number of vitellogenic follicles at 240 dah. Scale bars = 50 μm. AI, aromatase inhibitor, Letrozole. Vtg, vitellogenin. Oc, oocyte. Ocv, ovarian cavity. Sg, spermatogonia. Sc, spermatocyte. Sz, spermatozoa. Ed, efferent duct. WT, wild-type. dah, days after hatching. (TIF) [file pgen.1011210.s007.tif]

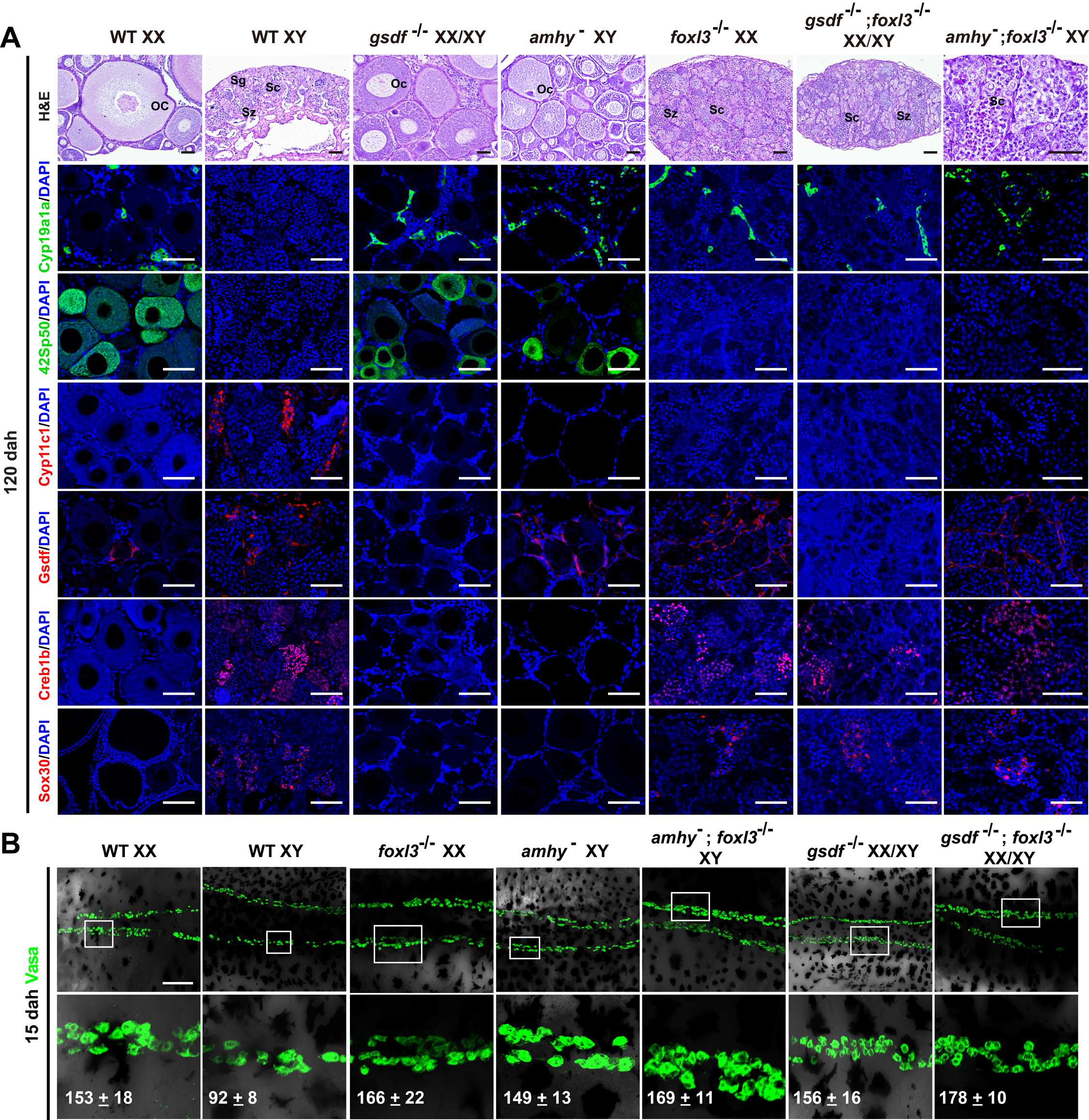

Supplement: S8 Fig — (A) Histological examination of gonads from WT XX, WT XY, XY amhy−, XX/XY gsdf-/-, XX foxl3-/-, XY amhy−;foxl3-/- and XX/XY gsdf-/-;foxl3-/- tilapia at 120 dah using H&E staining. Oc, oocyte. Sg, spermatogonia. Sc, spermatocyte. Expression of somatic cell and germ cell markers in the gonads of these mutants were analyzed by IF. Cyp19a1a, a female somatic specific marker. 42Sp50, an oocyte marker. Cyp11c1, a Leydig cell marker. Gsdf, a somatic cell marker. Creb1b, a spermatocyte marker. Sox30, a male germ cell marker expressed highly in spermatozoa. Nuclei were counterstained with DAPI. Scale bars = 40 μm. (B) The germ cells in WT XX, WT XY, XY amhy−, XX/XY gsdf-/-, XX foxl3-/-, XY amhy−;foxl3-/- and XX/XY gsdf-/-;foxl3-/- mutants were shown by whole-mount IF with Vasa antibody at 15 dah. The germ cell number in each genotype was counted (n = 4). WT, wild-type. dah, days after hatching. Scale bars = 200 μm. (TIF) [file pgen.1011210.s008.tif]

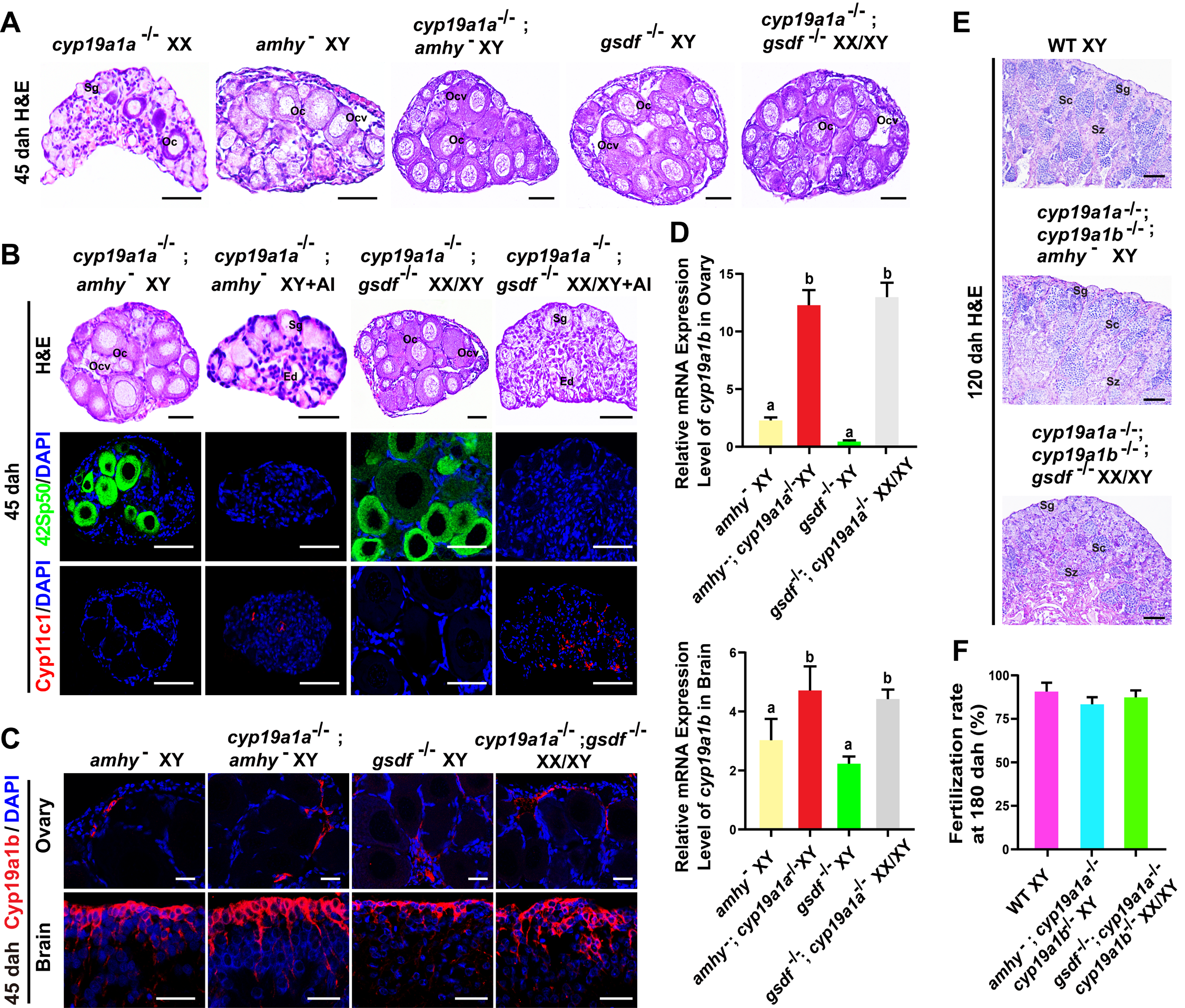

Supplement: S9 Fig — (A) Histological examination of gonads from XX cyp19a1a-/-, XY amhy−, XY amhy−;cyp19a1a-/-, XY gsdf-/- and XX/XY gsdf-/-;cyp19a1a-/- mutants at 45 dah by H&E staining. Scale bars = 40 μm. (B) AI treatment resulted in testis development in amhy−;cyp19a1a-/- and gsdf-/-;cyp19a1a-/- double mutants as revealed by 42Sp50 and Cyp11c1 staining at 45 dah. These two double mutants were treated with AI from 5 to 30 dah. AI, aromatase inhibitor, letrozole. Scale bars = 40 μm. (C) Expression of Cyp19a1b protein in the brains and ovaries of amhy−, gsdf-/- single mutants, amhy−;cyp19a1a-/- and gsdf-/-;cyp19a1a-/- double mutants at 45 dah detected by IF. Nuclei were counterstained with DAPI. Scale bars = 20 μm. (D) Real-time PCR analysis of cyp19a1b mRNA expression level in the brains and gonads of XY amhy−, XY gsdf-/- single mutants and XY amhy−;cyp19a1a-/- and XX/XY gsdf-/-;cyp19a1a-/- double mutants. Expression was normalized to β-actin. Data were expressed as the mean ± SD. Different letters above the error bars indicate statistical differences at P<0.05 as determined by one-way ANOVA followed by Tukey test. (E) Histological examination of gonads from WT XY, XY amhy−;cyp19a1a-/-;cyp19a1b-/- and XX/XY gsdf-/-;cyp19a1a-/-;cyp19a1b-/- triple mutants at 120 dah by H&E staining. Scale bars = 50 μm. (F) Fertilization rate of sperm from WT XY, XY amhy-;cyp19a1a-/-;cyp19a1b-/- and XX/XY gsdf-/-;cyp19a1a-/-;cyp19a1b-/- males at 180 dah (n = 3 for each genotype). Sg, spermatogonia. Sc, spermatocyte. Sz, spermatozoa. Oc, oocyte. Ocv, ovarian cavity. dah, days after hatching. (TIF) [file pgen.1011210.s009.tif]

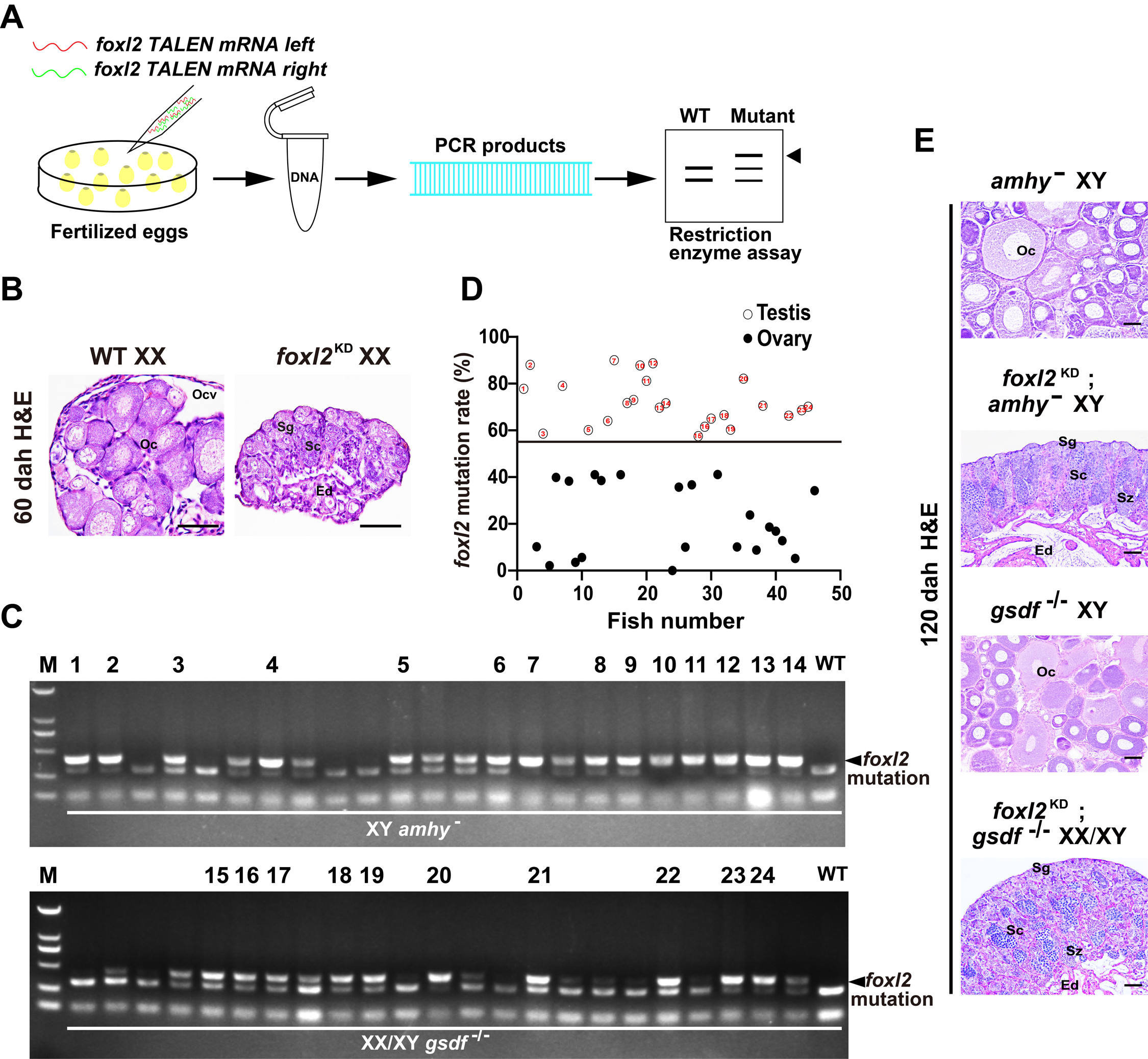

Supplement: S10 Fig — (A) The mixture of foxl2 left and right TALENs mRNA was injected into fertilized eggs from amhy− XY neo-females crossed with XX neo-males or from gsdf+/- XX females crossed with gsdf-/- XY males. DNA fragments spanning the target were amplified for mutation analysis. The mutations induced in foxl2 by TALENs were assayed by restriction enzyme assay as reported previously [56]. The undigested band indicated by arrow head suggested mutations in the target. WT, wild-type. (B) Histological examination of gonad sections from WT XX and XX foxl2KD mutants at 60 dah by H&E staining. The XX F0 foxl2 mutant fish showed testicular development, while the WT XX fish showed ovarian development with follicles at 60 dah. Scale bars = 40 μm. (C) Detection of foxl2 mutations in F0 fish by restriction enzyme assay. The XY amhy- or XX/XY gsdf-/- mutants were selected for foxl2 mutation analysis. The arrow head indicated the undigested bands. The numbers above the gel lanes indicate the fish with high mutation rate of foxl2. M, DNA marker. (D) Statistical analysis of the gonadal phenotypes of amhy and gsdf mutants with different foxl2 mutation rates. The foxl2 mutation rate in S10C Fig was calculated by quantifying intensity of uncleaved band. The number in the circle is corresponding to the number in S10C Fig. (E) Histological examination of gonads from XY amhy−, XY amhy−;foxl2KD, XY gsdf-/-, XX/XY gsdf-/-;foxl2KD tilapia at 120 dah by H&E staining. Scale bars = 40 μm. Oc, oocyte. Sg, spermatogonia. Sc, spermatocyte. Sz, spermatozoa. Ed, efferent duct. KD, knockdown. dah, days after hatching. (TIF) [file pgen.1011210.s010.tif]

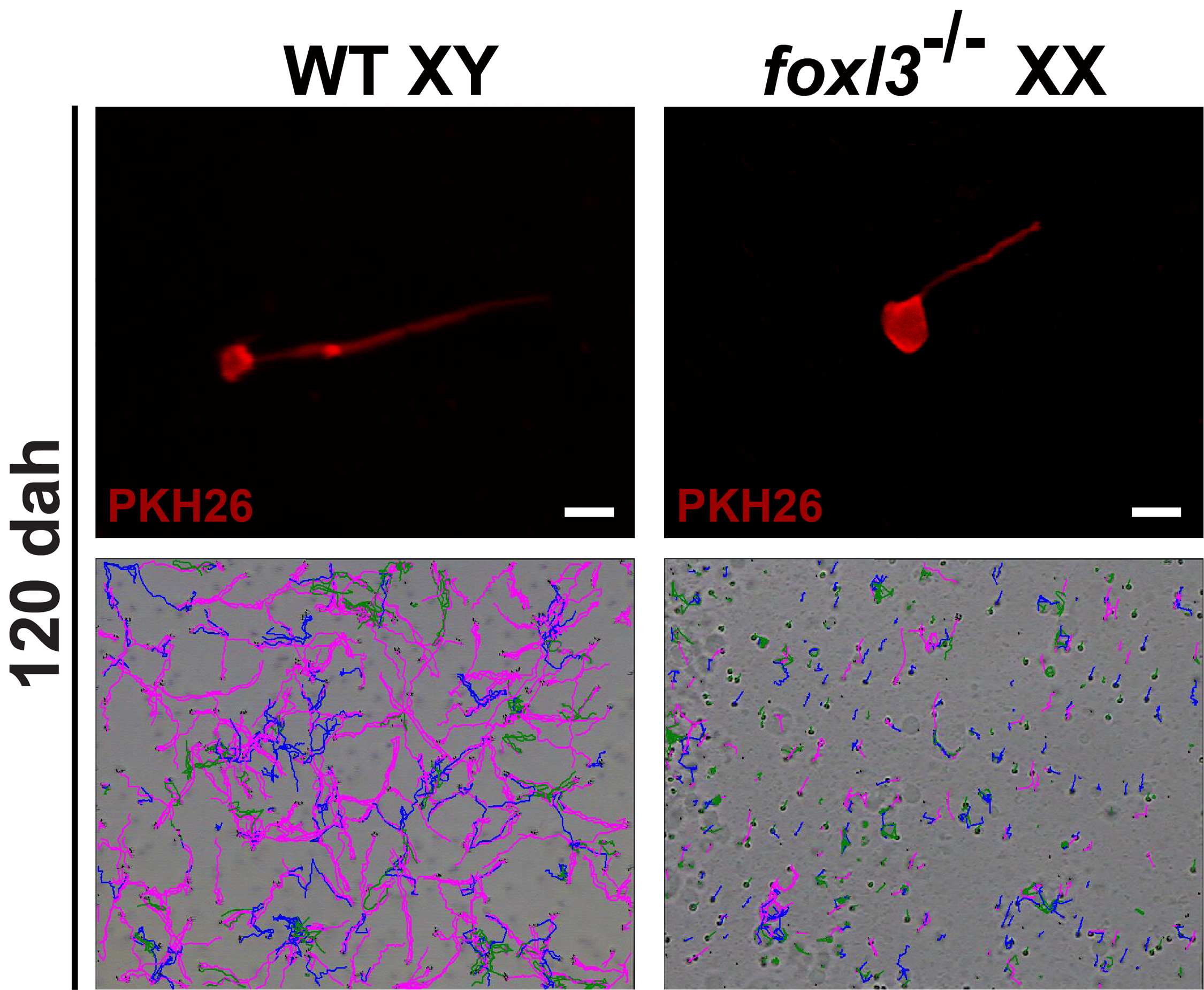

Supplement: S11 Fig — Sperm of WT XY and XX foxl3-/- fish were labeled by PKH26 in red. Dynamic trajectory curve of the sperm of WT XY and XX foxl3-/- fish. The pink line represents the dynamic trajectory curve of normal sperm. The blue line and green line represent the dynamic trajectory curve of abnormal sperm. Scale bars = 10 μm. dah, days after hatching. WT, wild-type. (TIF) [file pgen.1011210.s011.tif]

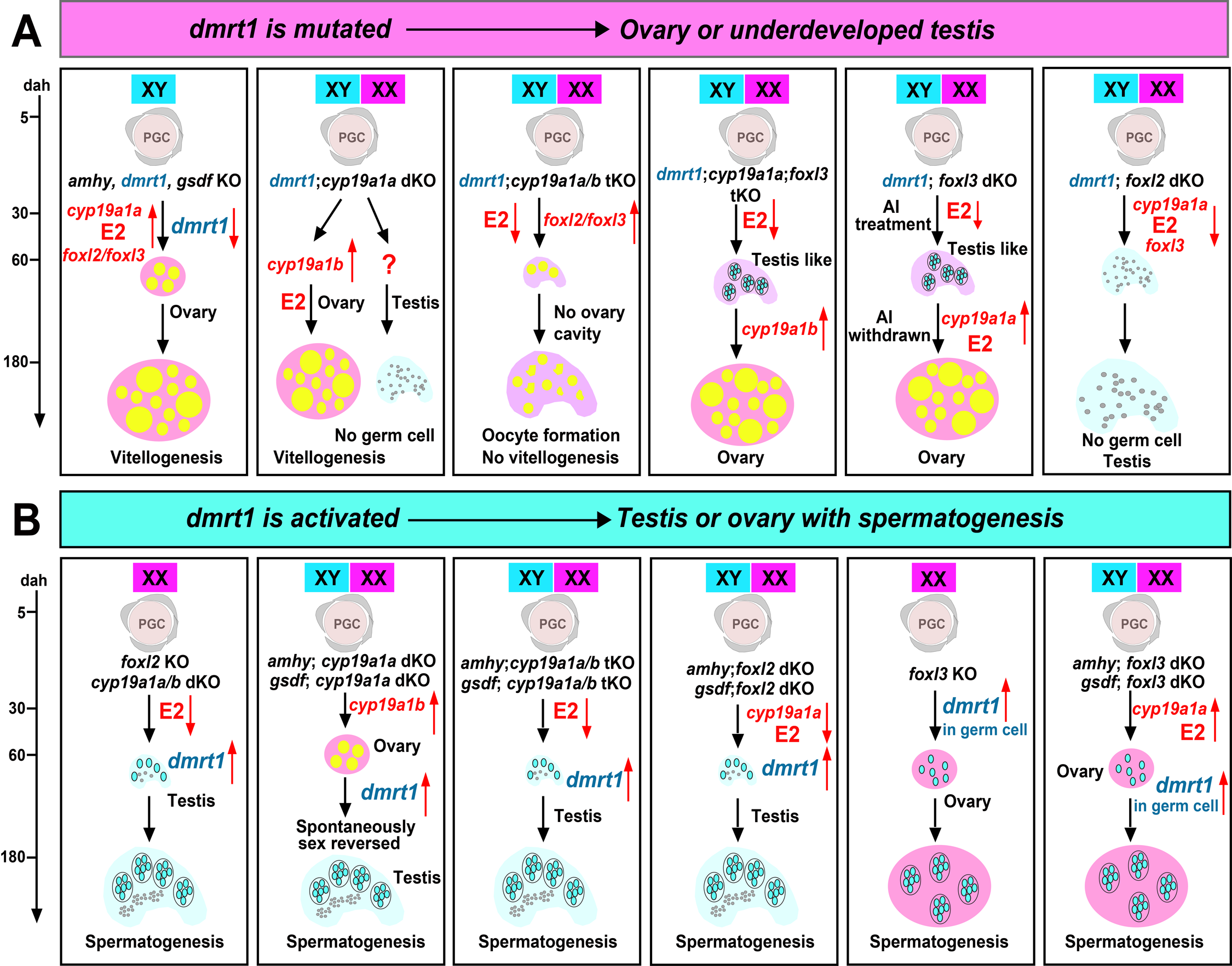

Supplement: S12 Fig — (A) Once dmrt1 was mutated, the gonad could not be rescued to a functional testis by mutation of female pathway gene identified so far. In male pathway genes amhy and gsdf mutants, cyp19a1a/estrogen was up-regulated to repress dmrt1 expression and promote female fate. In dmrt1 mutants, cyp19a1a/estrogen was up-regulated to induce ovary development. In dmrt1;cyp19a1a double mutants, the gonad developed either as ovary with vitellogenesis or underdeveloped testis with no germ cell. In dmrt1;cyp19a1a;cyp19a1b triple mutants, the gonad developed as ovary with previtellogenic follicles. In dmrt1;cyp19a1a;foxl3 triple and AI treated-dmrt1;foxl3 double mutants, the gonad developed eventually as ovary. In the dmrt1;foxl2 double mutants, the gonad developed as underdeveloped testis with no germ cell. (B) Once dmrt1 was present, sex reversal caused by mutation of other male pathway genes amhy and gsdf could be rescued. In female pathway genes foxl2 and cyp19a1a/cyp19a1b mutants, estrogen was down-regulated and dmrt1 was up-regulated to promote male fate. In amhy;cyp19a1a, gsdf;cyp19a1a, amhy;cyp19a1a;cyp19a1b, gsdf;cyp19a1a;cyp19a1b mutants, the gonad developed as functional testis due to up-regulation of dmrt1 in somatic and germ cell. In foxl3 single, amhy;foxl3 and gsdf;foxl3 double mutants, spermatogenesis occurs in ovarian environment due to up-regulation of dmrt1 in germ cell. KO, knockout. DKO, double knockout. TKO, triple knockout. E2, 17β-estradiol. AI, aromatase inhibitor. dah, days after hatching. (TIF) [file pgen.1011210.s012.tif]

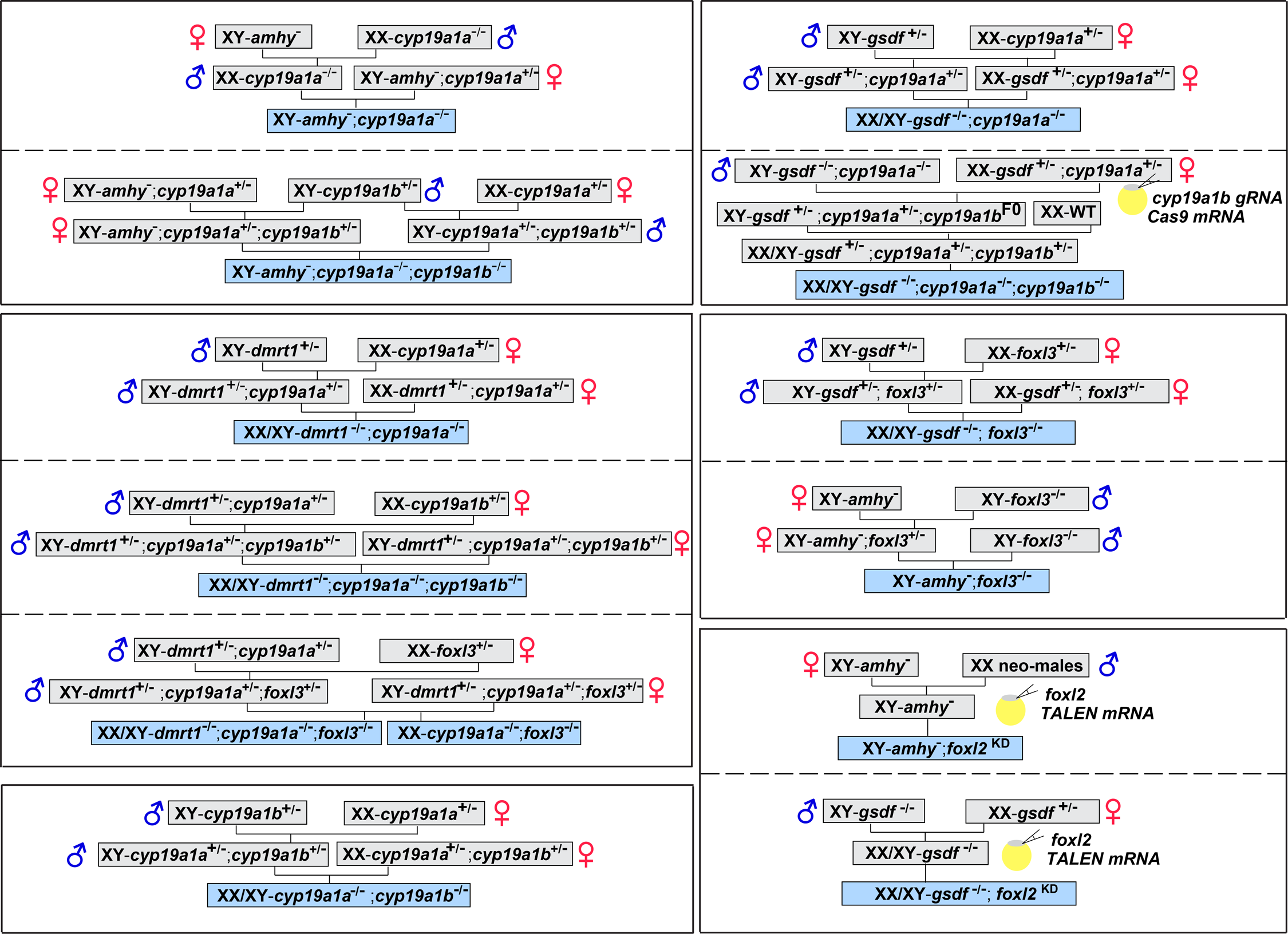

Supplement: S13 Fig — (TIF) [file pgen.1011210.s013.tif]
