# Supplementary material for: Dmrt1 is the only male pathway gene tested indispensable for sex determination and functional testis development in tilapia
Source: PLoS Genet. 2024 Mar 27;20(3):e1011210. doi: 10.1371/journal.pgen.1011210 (PMC10971778; doi:10.1371/journal.pgen.1011210)
Supplement: S1 Table — (DOC) [file pgen.1011210.s014.doc]

**S1 Table. Primers used in this study**

| **Primer name** | **Sequence (5'-3')** | **Purpose** |
| --- | --- | --- |
| gRNA-cyp19a1b-F | TAATACGACTCACTATAGGATACGTGTCTCCACTGAAGTTTTAGAGCTAGAAATAGC | CRISPR/Cas9 |
| gRNA-R | AGCACCGACTCGGTGCCAC |  |
| amhy-test-F | GTGAGAAGGGGAGGTCTGAGGATG | Mutant screening |
| amhy-test-R | GCTGCTGAGGATGATGATGGTG |  |
| dmrt1-test-F | GCCAAAAAGCAGGGTGTGGAGG |  |
| dmrt1-test-R | CCTGTGCGGCTGTGTGTGGTC |  |
| gsdf-test-F | CGTGGTTCTCAGACCCTGAC |  |
| gsdf-test-R | GAATCACACACTGAATTTAC |  |
| cyp19a1a-test-F | TGTAGGCTTAGACGCCGTGGTG |  |
| cyp19a1a-test-R | TTGTAGTAGTTGCTGGCTGTGC |  |
| cyp19a1b-test-F | ATGTGGCTCAGTGCTATGTTAC |  |
| cyp19a1b-test-R | TATTTGTTAGTGCCTCACCT |  |
| foxl3-test-F | CCGTGTGCACCTGCAGACAC |  |
| foxl3-test-R | CCTTAAATGCGGGGTCCAGC |  |
| foxl2-TALEN-F1 | CGACCAAGGAGAAAGAGCGCC |  |
| foxl2-TALEN-R1 | CTGTCCCCTCCGAACAAGGCC |  |
| cyp19a1b-qPCR-F | CATGGTGGCTGATAGAGCGT | Real-time PCR |
| cyp19a1b-qPCR-R | TGGACCGAGTCCTGCTAAGA |  |
| bmp15-qPCR-F1 | ACGAGCAGAAAGCGGACCAGA |  |
| bmp15-qPCR-R1 | GCGGAGAAGAGCGAAGGTGAAC |  |
| gdf9-qPCR-F1 | CATACGACGAGGACCTGGAA |  |
| gdf9-qPCR-R1 | GAGGCATTTCTGGAACCGAC |  |
| 42sp50-qPCR-F1 | CCGCAGAGAACTGTAACGCAAACC |  |
| 42sp50-qPCR-R1 | GTGAATCACTAGCCGACGGCAGC |  |
| foxl3-qPCR-F1 | CGGCCAAGGTGCGCAGATTCT |  |
| foxl3-qPCR-R1 | GTTCTCGCTGTCCTTGATGGC |  |
| gsdf-qPCR-F1 | GCTGCCGTCTTAGGTGACAG |  |
| gsdf-qPCR-R1 | GCAGTATGCTTGGCTGTGTG |  |
| amh-qPCR-F1 | CACCCAGCTGCAGTACACGTAT |  |
| amh-qPCR-R1 | TCAAAGGTCAACGTGATTGTTCC |  |
| foxl2-qPCR-F1 | AAGAGGAGCCGGTTCAGGACAA |  |
| foxl2-qPCR-R1 | GCTCTCCCGGATAGCCATGG |  |
| stAR1-qPCR-F | CTGAAACTGTTGCTGCGAATGGA |  |
| stAR1-qPCR-R | GGTCTCTGCGGATACCTCGTG |  |
| stAR2-qPCR-F | AGCAGGACTTCAACGCACTG |  |
| stAR2-qPCR-R | CTGGCTTGGCCTCGTTATGA |  |
| 3β-HSD-I-qPCR-F | TGGAGGACTGCAAAGGTG |  |
| 3β-HSD-I-qPCR-R | TCAATGATGGATGCGATG |  |
| 3β-HSD-II-qPCR-F | ATCTCAGCACAGAGCGGACAAG |  |
| 3β-HSD-II-qPCR-R | CACTCCACACAGGCTTTGATGAC |  |
| cyp17a1-qPCR-F | TGTCATCAACCAGCATGTGCAC |  |
| cyp17a1-qPCR-R | ACTTCCACGTAGCACTGTAGTC |  |
| cyp17a2-qPCR-F | TGAGGATCCGGCCAGTGA |  |
| cyp17a2-qPCR-R | GCGTGGCCTCCAATGC |  |
| β-actin-qPCR-F | CATCCCGTCCTGCTCACA |  |
| β-actin-qPCR-R | AGGCGTACAGGGACAGCA |  |
| bmp15-RT-PCR-F1 | ACGAGCAGAAAGCGGACCAGA | RT-PCR |
| bmp15-RT-PCR-R1 | GCGGAGAAGAGCGAAGGTGAAC |  |
| foxl3-RT-PCR-F1 | ATGGATGCAGAGGAGAAGTC |  |
| foxl3-RT-PCR-R1 | GCAGTAGGAGGTCATGGAGGTG |  |
| creb1b-RT-PCR-F | GGTGGAGCCATTCAGCTGGC |  |
| creb1b-RT-PCR-R | ATTTATGGCAGTAAAGGTC |  |
| dmrt6-RT-PCR-F | ACCCGGAGCCAGAAGGGT |  |
| dmrt6-RT-PCR-R | CATATTTTTTATGATTCATTCCC |  |
| foxl2-RT-PCR-F | AGAAAGAGCGCCCAAAAGAG |  |
| foxl2-RT-PCR-R | CATTCGTTAAGACTGAGGTTGT |  |
| dmrt1-RT-PCR-F | AGTTGGAGCAGACTGCCTGT |  |
| dmrt1-RT-PCR-R | CGTCGACGGTGAAGCCGGCTGC |  |
| cyp11c1-RT-PCR-F | GAGATACGCAACCACAGCAAGG |  |
| cyp11c1-RT-PCR-R | GCAGGTAGAGGAGAGGAGGAGT |  |
| vtg1-RT-PCR-F | CCTTTCCATCCAGCCACC |  |
| vtg1-RT-PCR-R | AGATGAATGCGTTCGGCC |  |
| vtg2-RT-PCR-F | GGAGGCTGGAACTCAGGGTG |  |
| vtg2-RT-PCR-R | TGGACCGATGCCTCCTTG |  |
| vtg3-RT-PCR-F | CAGAAGAGGGCGGCATTG |  |
| vtg3-RT-PCR-R | TTCCACCCACATCTCATCCA |  |
| β-actin-RT-PCR-F | GGCATCACACCTTCTACAACGA |  |
| β-actin-RT-PCR-R | ACGCTCTGTCAGGATCTTCA |  |
| foxl3-ISH-F1 | ATGGATGCAGAGGAGAAGTC | *In situ* hybridization |
| foxl3-ISH-R1 | GCAGTAGGAGGTCATGGAGGTG |  |
| dmrt1-ISH-F1 | AGGACAAACAGAGTAAGCAGG |  |
| dmrt1-ISH-R1 | GCTGCCATGGTCTCAGAGC |  |
| AMH-F5 | ATGGCTCCGAGACCTTGACTG | Genetic sex identification |
| AMH-R3 | CAGAAATGTAGACGCCCAGGTAT |  |

F, forward primer; R, reverse primer
