# Supplementary material for: Dmrt1 is the only male pathway gene tested indispensable for sex determination and functional testis development in tilapia
Source: PLoS Genet. 2024 Mar 27;20(3):e1011210. doi: 10.1371/journal.pgen.1011210 (PMC10971778; doi:10.1371/journal.pgen.1011210)
Supplement: S2 Table — (DOC) [file pgen.1011210.s015.doc]

**S2 Table. Antibodies used in this study**

| Antibodies | Source | Catalog | Dilution ratio |
| --- | --- | --- | --- |
| Rabbit-anti-Cyp19a1a | Our lab | N/A | 1:2000 |
| Rabbit-anti-Cyp19a1b | Our lab | N/A | 1:1000 |
| Rabbit-anti-Amh | Our lab | N/A | 1:1000 |
| Rabbit-anti-Gsdf | Our lab | N/A | 1:2000 |
| Rabbit-anti-3β-HSD-I | Our lab, unpublished | N/A | 1:2000 |
| Rabbit-anti-Sox30 | Our lab | N/A | 1:500 |
| Rabbit-anti-Vasa | Our lab | N/A | 1:2000 |
| Rabbit-anti-Cyp11c1 | Our lab | N/A | 1:1000 |
| Rabbit-anti-42Sp50 | Our lab, unpublished | N/A | 1:1000 |
| Rabbit-anti-Zar1 | Our lab, unpublished | N/A | 1:500 |
| Rabbit-anti-Creb1b | Our lab unpublished | N/A | 1:1000 |
| Donkey anti-Rabbit IgG (H+L) ReadyProbes Secondary Antibody, Alexa Fluor 594 | Thermo Fisher scientific | R37119 | 1:500 |
| Donkey anti-Rabbit IgG (H+L) ReadyProbes Secondary Antibody, Alexa Fluor 488 | Thermo Fisher scientific | R37118 | 1:500 |
